# Novel *BEST1* Variant Characterization in a Large French Cohort in Light of Updated Bestrophin-1 Structure–Function Correlation

**DOI:** 10.1167/iovs.66.12.4

**Published:** 2025-09-02

**Authors:** Joan Bitan, Anaïs F. Poncet, Claire Lecigne, Aurore Devos, Isabelle Meunier, Xavier Zanlonghi, Olivier Grunewald, Vasily Smirnov, Claire-Marie Dhaenens

**Affiliations:** 1 University of Lille, INSERM, CHU-Lille, U1172 - Lille Neuroscience & Cognition Research Center (LilNCog), Lille, France; 2 Institute for Neurosciences of Montpellier, University of Montpellier, INSERM, Montpellier, France; 3 National Reference Center for Inherited Sensory Diseases, University of Montpellier, CHU, Montpellier, France; 4 Service d'Ophtalmologie, Centre de compétence maladie rare, Centre Hospitalier Universitaire de Rennes, Rennes, Bretagne, France; 5 Exploration de la Vision et Neuro-Ophtalmologie, CHU de Lille, Lille, France

**Keywords:** *BEST1*, variants, pathogenicity assessment, structure, function

## Abstract

**Purpose:**

To update knowledge on bestrophin-1 structure and function with the aim of assessing the pathogenicity of variants reported in the Leiden Open Variation Database (LOVD) and in a large French cohort of bestrophinopathies.

**Methods:**

All unique variants reported in the latest version (October 2024) of the BEST1-LOVD database were uploaded and curated. We described all *BEST1* variants identified in French patients analyzed at Lille University Hospital, between 2008 and 2024. A comprehensive analysis of each variant was performed based on in silico tools (at DNA, RNA, and protein levels), as well as a literature review providing clinical data and functional assays. All of these data were used to classify the variant pathogenicity according to the American College of Medical Genetics and Genomics (ACMG) criteria.

**Results:**

We detailed 488 variants from the LOVD. Among 450 French patients, we identified 150 different variants, 40 of which were novel. We classified only eight variants as variants of unknown significance, four of which were already in the LOVD. We identified specific recurrent variants in the French population: p.(Gly26Asp), p.(Val90Met), p.(Val137Met), and p.(Ile230del), the last of which was present in 17 patients (3.8%). All new variants cause changes in chemical interactions within the protein and are associated with clinical pictures of bestrophinopathy.

**Conclusions:**

The study and comparison of these two large cohorts highlight variants specific to the French population, as well as differences in protein distribution, which are undoubtedly influenced by several population-specific factors. Through multiple in silico analyses, we were able to reclassify 93.3% of variants as likely pathogenic or pathogenic, thereby strengthening clinical diagnoses.

The *BEST1* gene (OMIM *607854), also referred to as *VMD2*, is located on chromosome 11q13^1^^,^^2^ and is expressed in the retinal pigment epithelium (RPE).^2^^,^^3^ *BEST1* has eight transcripts (UCSC Genome Browser on Human GRCh38/hg38). The Matched Annotation from NCBI and EBI (MANE) transcript NM_004183.4 includes 11 exons, with the first being non-coding. In humans, it encodes the bestrophin-1 (BEST1) protein, composed of 585 amino acids. It is a calcium-activated chloride channel.^4^^–^^11^ The channel consists of five BEST1 protein subunits, each containing four transmembrane domains and extra- and intracellular loops forming a channel that facilitates ion passage from the extracellular to the intracellular space, passing through an internal cavity bordered by two constriction zones, the neck and the aperture opening into the cytosol.

*BEST1* is a major gene involved in inherited macular dystrophies, with variants frequently identified in diagnostic laboratories. Some variants are novel with uncertain pathogenicity. The complexity of *BEST1* arises from the heterogeneity of associated phenotypes and inheritance patterns. *BEST1* variants are inherited in both autosomal dominant and recessive manners and are linked to five distinct phenotypes referred to as “bestrophinopathies”: Best vitelliform macular dystrophy (BVMD) (OMIM #153700),^1^^,^^2^ adult-onset vitelliform macular dystrophy (AVMD) (OMIM #153700),^12^^,^^13^ autosomal recessive bestrophinopathy (ARB) (OMIM #611809),^9^ autosomal dominant vitreoretinochoroidopathy (ADVIRC) (OMIM #193220),^14^ and retinitis pigmentosa (RP) (OMIM #613194).^15^ Understanding the gene and protein structure is crucial for analyzing variants of uncertain significance (VUSs) and reclassifying them as likely pathogenic or benign. This study aims to (1) curate the international Leiden Open Variation Database (LOVD), (2) report new variants in a large cohort of French patients, and (3) classify these variants using in silico, structural, and functional data.

## Materials and Methods

More details are provided in the Supplementary Materials and Methods file.

### Literature Search and Data Collection

All publications reporting *BEST1* variants in patients with inherited retinal disease (IRD) were uploaded to the LOVD (https://databases.lovd.nl/shared/genes/BEST1) through December 2024. The last LOVD version used was dated October 29, 2024. Duplicates were removed and nomenclature errors corrected.

### Patients

We examined *BEST1* variants identified in the Lille University Hospital Laboratory. These variants were identified in a French cohort of 15,000 patients with IRDs analyzed between 2008 and 2024. They were referred to our laboratory with diagnoses of “Best macular dystrophy,” “macular dystrophy,” “bestrophinopathy,” “retinitis pigmentosa,” or “retinal dystrophy.” They included only affected probands. All patients carried one or two *BEST1* variants. Written informed consent was obtained from all participants before enrollment following the tenets of the Declaration of Helsinki. DNA collection from the Lille ophthalmology database received the agreement number DEC22-323 and the use of the Lille data was approved under authorization code CNIL DR-2023-061.

### Variant Identification in Our Laboratory

Genetic diagnosis was performed using multiple techniques: HaloPlex-based next-generation sequencing of 150, 208, or 230 IRD-associated genes on the Ion Torrent/NextSeq and Nova platforms, and Sanger sequencing of the 11 *BEST1* gene coding exons. Copy number variants (CNVs) were detected by multiplex ligation-dependent probe amplification (MLPA); (SALSA MLPA Probemix P367; MRC Holland, Amsterdam, The Netherlands), or based on next-generation sequencing (NGS) data. Segregation analyses or confirmations were conducted using direct Sanger sequencing.

### Variant Analysis

LOVD and French variants were collected and analyzed exhaustively. A large amount of information is provided in a table in the Supplementary Materials and Methods.

### Protein Organization, In Silico Predictions, and Variant Pathogenicity Classification

Protein organization was defined based on the latest Uniprot version (https://www.uniprot.org/uniprotkb/O76090/feature-viewer). The three-dimensional (3D) structure of the BEST1 channel and changes in chemical interactions due to amino acid substitutions were assessed using AlphaFold (https://alphafold.ebi.ac.uk/entry/O76090), UCSF ChimeraX 1.7.1, and DynaMut2 (http://biosig.unimelb.edu.au/dynamut2) with the Protein Data Bank (PDB) file 8d1k. Pathogenicity classification was performed according to American College of Medical Genetics and Genomics (ACMG) criteria^16^ (see Supplementary Materials and Methods).

### Review of Functionally Tested *BEST1* Variants From Literature

All variants reported in human disorders and that affect trafficking to the RPE plasma membrane, oligomerization, channel activity, and intracellular calcium signaling were collected from the literature.

### Statistical Analysis

Statistical analyses were performed using Prism 10.4.1 for macOS (GraphPad, Boston, MA, USA). More details are provided in the Supplementary Materials and Methods.

## Results

### Update on the General Organization and Function of the BEST1 Channel

**Figure 1.**
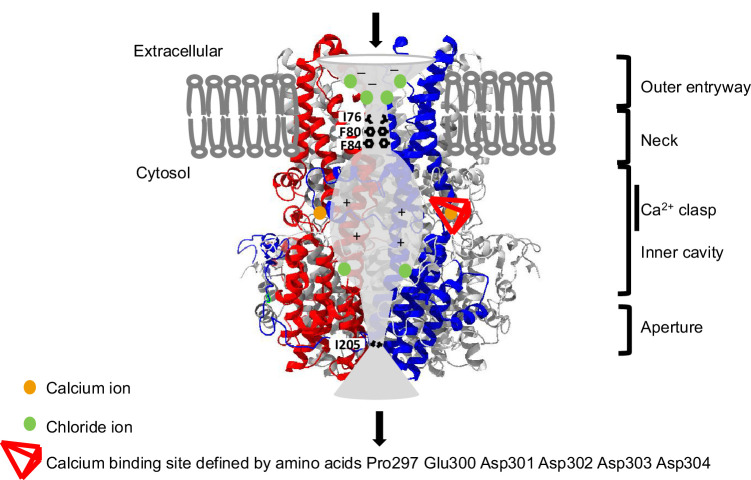
Schematic representation of two of the five subunits of the BEST1 channel. A sagittal section of the channel is shown, using a 3D structure obtained from UCSF ChimeraX software as a background and a superimposed drawing. Two of five monomers are shown (in *red* and *blue*). Each BEST1 monomer is composed of 17 helices (based on Uniprot and AlphaFold). The pore presents two narrow hydrophobic constrictions, the “neck,” located at the inner leaflet of the membrane, and the “aperture,” located at the cytosolic end of the channel, both of which could serve as gates that open and close in response to Ca^2+^ (*orange dots*). Each is wide enough to allow the passage of a dehydrated chloride ion (ionic flux depicted as a *black arrow*). The “inner cavity” is highly positively charged (+) to attract anions from the interior of the cell. The calcium clasps are located on the cytosolic side of the channel, beneath the neck. Three chloride ion binding sites are shown by protomers (*green dots*). A minus sign (–) indicates a negative charge.

A comprehensive overview of updated BEST1 channel organization and function is detailed in Figure 1, Supplementary Results, and Supplementary Table S1.

### Variation Spectrum

#### LOVD Cohort

We curated the LOVD to remove redundancies and ensure the correct nomenclature. As a result of this update, 488 different variants were listed in the LOVD in 2205 patients (https://databases.lovd.nl/shared/genes/BEST1, December 2024) (see Supplementary Table S2). Missense variants were predominant (353/488, 72.3%), followed by frameshift 9.8% (48/488) and nonsense 4.1% (20/488) (Supplementary Table S3A, Supplementary Fig. S1A).

Variant distribution across the *BEST1* gene revealed significant clustering in specific protein regions (Supplementary Table S4A). Notably, 36.1% of variants (176/488) are located in the transmembrane TM2–TM3 cytosolic loop, a 137-amino-acid region. Additional enrichment was observed in the C-terminal domain (31.4%, 153/488) and N-terminal domain (12.5%, 61/488), which span 296 and 31 amino acids, respectively. In contrast, transmembrane domains TM1 to TM4 showed limited variant accumulation (ranging from 2.5% to 6.4%), each covering approximately 15 to 20 amino acids (Supplementary Table S1).

Structural mapping of these variants using software such as UCSF ChimeraX and DynaMut2 indicated that those in the cytosolic TM2–TM3 loop are located around the internal cavity and near the second restriction site (Supplementary Fig. S2, Supplementary Table S1). C-terminal variants are cytosolic and are located at the periphery of the channel, rather than at the edge of the internal cavity. Some are connected to other channel subunits. N-terminal variants are localized within the calcium-binding site or channel neck. We then evaluated whether the type of variant (missense vs. truncating) was related to its localization in specific main domains. A χ^2^ test showed a statistically significant association (*P* < 0.05) supporting the hypothesis of domain-specific functional constraints (Supplementary Table S5A). Regarding phenotype, BVMD cases were most frequently reported in the LOVD cohort (41.8% and 204 different variants), followed by ARB with 162 variants (33.2%). A subset of 6.6% variants was associated with multiple phenotypes (e.g., AVMD and BVMD, BVMD, ARB); 62 variants lacked phenotypic annotation or it was difficult to establish a correlation between phenotype and genotype (Supplementary Tables S2, S6A).

#### French Cohort

In the French cohort, we identified 150 distinct *BEST1* variants in 450 patients (Supplementary Tables S3B, S7). Missense variants predominated (132/150, 88%), as they were found in 401 patients, and frameshift variants represented 4.7% (7/150). Similar to the LOVD cohort, variants were overrepresented in the TM2–TM3 cytosolic loop (57/150, 38%), the C-terminal domain (41/150, 27.3%), and the N-terminal domain (25/150, 16.7%) (Supplementary Tables S4B, S4C; Supplementary Figs. S1B, S3). No significant association was observed between variant type and domain localization (*P* = 0.4286) (Supplementary Table S5B). Furthermore, Table 1 presents 40 novel variants not previously reported in the literature. The phenotypic distribution in the French cohort mirrored that of the LOVD cohort (*P* = 0.2901): 58.7% of BVMD cases (88/150 variants), 27.3% of ARB cases (41/150 variants), and around 8.7% with multiple phenotypes (13/150 variants) (Supplementary Tables S6B, S6C). Based on our national registry, *BEST1-*related diseases accounted for 0.21% of IRD patients, 6.8% of macular dystrophy patients, and 40.9% of bestrophinopathy patients (with *PRPH2* representing 7.1% of these patients).

**Table 1.** Novel *BEST1* Variants in the French Cohort (Not Described in the Literature or in the LOVD)

Variation in cDNA	Variation in Protein	Type of Variation	Inheritance	If AR, What Is the Second Allele?	Number of Patients	Diagnosis	Exon	Physicochemical Deviation	Conservation in Evolution	Position From Splice Site	Protein Domain	ACMG Classification
c.43G>A	p.(Gly15Ser)	Missense	AD	—	1	BVMD	2	Medium	Moderately	79 bp from acceptor	Cytosolic N-termini	SNV4
c.56G>A	p.(Arg19His)	Missense	AD	—	1	MD	2	Not very important	Moderately	92 bp from acceptor	Cytosolic N-termini	SNV4
c.77G>A	p.(Gly26Asp)	Missense	AD	—	8	BVMD	2	Important	Strongly	76 bp from donor	Cytosolic N-termini	SNV4
c.92T>C	p.(Leu31Pro)	Missense	AD	—	1	BVMD	2	Important	Strongly	61 bp from donor	Cytosolic N-termini	SNV4
c.99T>G	p.(Tyr33*)	Nonsense	AR	c.103G>A, p.(Glu35Lys)	1	ARB	2	NA	NA	54 bp from donor	TM1	SNV5
c.104A>C	p.(Glu35Ala)	Missense	AD	—	1	RP	2	Important	Strongly	49 bp from donor	TM1	SNV4
c.184T>G	p.(Phe62Val)	Missense	AR	c.223C>T, p.(Leu75Phe)	1	ARB	3	Medium	Strongly	32 bp from acceptor	TM2	SNV4
c.197C>T	p.(Thr66Ile)	Missense	AR	c.1632G>A, p.(Met544Ile)	1	ARB	3	Medium	Weakly	45 bp from acceptor	TM2	SNV4
c.209_247+65del	p.(Asp70_Leu82del)	Deletion	AD	—	2	BVMD	3 and intron 3	NA	NA	41 bp from donor	TM2	SNV4
c.218T>C	p.(Ile73Thr)	Missense	AD	—	2	BVMD	3	Medium	Moderately	30 bp from donor	TM2	SNV4
c.248-2A>G	p.(?)	Splice	AR	c.584C>T, p.(Ala195Val)	1	ARB	Intron 3 splice acceptor site	NA	NA	No results	Cytosolic loop TM2–TM3	SNV5
c.268G>A	p.(Val90Met)	Missense	AD	—	6	BVMD	4	Not very important	Strongly	21 bp from acceptor	Cytosolic loop TM2–TM3	SNV4
c.275G>T	p.(Arg92Leu)	Missense	AD	—	2	BVMD	4	Important	Strongly	28 bp from acceptor	Cytosolic loop TM2–TM3	SNV4
c.282G>C	p.(Trp94Cys)	Missense	AR	c.637_639del, p.(Gl213del)	2	ARB	4	Very important	Strongly	35 bp from acceptor	Cytosolic loop TM2–TM3	SNV4
c.305G>T	p.(Trp102Leu)	Missense	AD	—	1	BVMD	4	Not very important	Strongly	58 bp from acceptor	Cytosolic loop TM2–TM3	SNV4
c.324C>A	p.(Ser108Arg)	Missense	AD	—	2	BVMD	4	Important	Weakly	77 bp from acceptor	Cytosolic loop TM2–TM3	VUS
c.382C>A	p.(Leu128Ile)	Missense	AR	c.422G>A, p.(Arg141His)	1	ARB	4	Not very important	Weakly	100 bp from donor	Cytosolic loop TM2–TM3	SNV4
c.409G>A	p.(Val137Met)	Missense	AD	—	4	BVMD	4	Not very important	Weakly	73 bp from donor	Cytosolic loop TM2–TM3	SNV4
c.448C>T	p.(Arg150Cys)	Missense	AD	In *cis* with c.934G>A p.(Asp312Asn)	1	AVMD	4	Very important	Moderately	34 bp from donor	Cytosolic loop TM2–TM3	SNV4
c.570C>G	p.(Asn190Lys)	Missense	AR	In *trans* with c.621T>C, p.(Ile201Thr)	2	ARB	5	Medium	Weakly	66 bp from donor	Cytosolic loop TM2–TM3	SNV4
c.676G>A	p.(Ala226Thr)	Missense	AD	—	1	BVMD	6	Medium	Moderately	40 bp from acceptor	Cytosolic loop TM2–TM3	SNV4
c.680A>C	p.(Tyr227Ser)	Missense	AD	—	1	BVMD	6	Very important	Strongly	35 bp from donor	Cytosolic loop TM2–TM3	SNV4
c.689_691del	p.(Ile230del)	Deletion	AD	—	17	BVMD	6	NA	NA	28 bp from donor	Cytosolic loop TM2–TM3	SNV5
c.691A>C	p.(Ser231Arg)	Missense	AD	—	1	BVMD	6	Important	Strongly	24 bp from donor	Cytosolic loop TM2–TM3	SNV4
c.692G>A	p.(Ser231Asn)	Missense	AD	—	1	BVMD	6	Medium	Moderately	23 bp from donor	Cytosolic loop TM2–TM3	SNV4
c.700C>G	p.(Leu234Val)	Missense	AD	—	3	BVMD	6	Not very important	Weakly	15 bp from donor	Cytosolic loop TM2–TM3	SNV4
c.718G>A	p.(Val240Met)	Missense	AR	c.1067G>T, p.(Arg356Leu)	2	ARB	7	Not very important	Strongly	4 bp from acceptor	TM3	SNV4
c.722C>T	p.(Thr241Ile)	Missense	AD	—	1	BVMD	7	Medium	Strongly	8 bp from acceptor	TM3	SNV4
c.731T>G	p.(Val244Gly)	Missense	AR	In complex allele with c.605G>A, p.(Arg202Gln); in *trans* with c.815T>C, p.(Val272Ala)	1	ARB	7	Important	Strongly	17 bp from acceptor	TM3	SNV4
c.815T>C	p.(Val272Ala)	Missense	AR	c.[605G>A; 731T>G], p[(Arg202Gln); (Val244Gly)]	1	ARB	7	Medium	Weakly	53 bp from donor	Extracellular loop TM3-TM4	SNV4
c.924C>G	p.(Asn308Lys)	Missense	AD	—	1	BVMD	8	Important	Moderately	25 bp from donor	Cytosolic C-termini	SNV4
c.934G>T	p.(Asp312Tyr)	Missense	AD	—	2	BVMD	8	Very important	Strongly	15 bp from donor	Cytosolic C-termini	SNV4
c.938G>A	p.(Arg313Lys)	Missense	AD	—	2	BVMD	8	Not very important	Strongly	8 bp from donor	Cytosolic C-termini	VUS
c.977A>C	p.(His326Pro)	Missense	AR	c.37C>T, p.(Arg13Cys)	1	ARB	9	Medium	Strongly	29 bp from acceptor	Cytosolic C-termini	SNV4
c.1037C>G	p.(Pro346Arg)	Missense	AD	—	1	BVMD	9	Medium	Strongly	64 bp from donor	Cytosolic C-termini	SNV4
c.1042A>G	p.(Thr348Ala)	Missense	AR	c.1470_1471del, p.(His490Glnfs*24)	1	ARB	9	Medium	Strongly	59 bp from donor	Cytosolic C-termini	SNV4
c.1087A>C	p.(Thr363Pro)	Missense	AR	c.1087A>C, p.(Thr363Pro)	2	ARB	9	Not very important	Weakly	14 bp from donor	Cytosolic C-termini	VUS
c.1101-1G>T	p.(?)	Splice	AD	—	3	BVMD	Intron 9 splice acceptor site	NA	NA	No results	Cytosolic C-termini	SNV5
c.1514_1515del	p.(Val505Glufs*9)	Deletion	AD en cis	c.903T>G p.(Asp301Glu)	1	AVMD	10	NA	NA	229 bp from donor	Cytosolic C-termini	SNV5
c.1632G>A	p.(Met544Ile)	Missense	AR	c.197C>T, p.(Thr66Ile)	1	ARB	10	NA	NA	108 bp from donor	Cytosolic C-termini	VUS

NA, not applicable; SNV, single nucleotide variant.

### Pathogenicity Assessment

We reclassified all LOVD variants using ACMG criteria.^16^ Among the 488 variants reported, we identified 305 variants (62.5%) as pathogenic (class 5), 93 variants (19.1%) as probably pathogenic (class 4), and 36 variants (7.4%) as VUSs (class 3). Fifty-four variants (11.1%) reported in the database were benign or likely benign, based on their high frequency or poor in silico predictions. Most of them (35, 7.2%) were added to the database but not described in the literature (Supplementary Fig. S1A, Supplementary Table S2). In the French cohort, we identified 84 pathogenic variants (56%), 56 likely pathogenic variants (37.3%), and eight VUSs (5.3%), four of which were already reported in the LOVD. Pathogenic and likely pathogenic variants therefore represented 93.3% of the total number. Two variants, p.(Leu207Ile) and p.(Val492Ile), were reclassified as benign due to high population frequency (Supplementary Fig. S1B, Supplementary Table S7). We report the changes in the 3D protein structure of these VUSs in Supplementary Figure S4. Three of them—p.(Arg355Cys), p.(Thr363Pro), and p.(Glu557Lys)—showed no destabilizing effect on protein (positive ΔΔG), in line with other predictions, and may subsequently be reclassified as likely benign. All phenotypes are represented in association with these 140 pathogenic or likely pathogenic variants (Supplementary Table S7).

### Recurrent Variants

#### Leiden Open Variation Database

Some of the causal variants identified in the LOVD are frequent and have been reported in more than 40 patients: c.73C>T, p.(Arg25Trp); c.422G>A, p.(Arg141His); c.584C>T, p.(Ala195Val); c.652C>T, p.(Arg218Cys); c.653G>A, p.(Arg218His); c.728C>T, p.(Ala243Val); c.763C>T, p.(Arg255Trp); and c.884_886del. Of these, p.(Arg25Trp), p.(Arg218Cys), and p.(Ala243Val) are transmitted in a dominant mode and are associated with BVMD or AVMD, and the other four variants exhibit autosomal recessive inheritance and are linked to ARB. The p.(Arg218His) variant is observed in both BVMD and ARB (Supplementary Table S2).

#### French Cohort

In the French cohort, the most frequent variant was p.(Arg218His), found in 37 patients (8.3%) (Table 2). The recurrent variants in the French cohort differed from those reported in the LOVD. Two of these frequent variants—p.(Gly26Asp) (*n* = 8, 1.8%) and p.(Ile230del) (*n* = 17, 3.8%)—are novel and are described below. Most of all these recurrent variants are associated with BVMD, except for p.(Val86Met), which was identified in patients with ADVIRC^14^ (Supplementary Table S7).

**Table 2.** Recurrent *BEST1* Variants in the French Cohort (Variants Found in Strictly More Than Five Patients in the French Cohort; *N* = 450)

Variation in cDNA	Variation in Protein	Number of Cases	Rate of Patients (*N* = 450)	Diagnosis	Protein Domain	Published?	ACMG Class
c.10A>G	p.(Thr4Ala)	6	1.3%	BVMD	Cytosolic N-termini	Yes	SNV5
c.44G>A	p.(Gly15Asp)	16	3.6%	BVMD/ARB	Cytosolic N-termini	Yes	SNV5
c.73C>T	p.(Arg25Trp)	7	1.6%	BVMD	Cytosolic N-termini	Yes	SNV5
c.77G>A	p.(Gly26Asp)	8	1.8%	BVMD	Cytosolic N-termini	No	SNV4
c.103G>A	p.(Glu35Lys)	6	1.3%	ARB	TM1	Yes	SNV5
c.238T>G	p.(Phe80Val)	12	2.7%	BVMD	TM2	Yes	SNV5
c.256G>A	p.(Val86Met)	11	2.5%	ADVIRC	Cytosolic loop TM2–TM3	Yes	SNV5
c.268G>A	p.(Val90Met)	6	1.3%	BVMD	Cytosolic loop TM2–TM3	No	SNV4
c.272C>T	p.(Thr91Ile)	6	1.3%	BVMD	Cytosolic loop TM2–TM3	Yes	SNV4
c.422G>A	p.(Arg141His)	8	1.8%	ARB/AVMD/BVMD	Cytosolic loop TM2–TM3	Yes	SNV5
c.652C>A	p.(Arg218Ser)	6	1.3%	BVMD	Cytosolic loop TM2–TM3	Yes	SNV5
c.652C>T	p.(Arg218Cys)	10	2.2%	ARB/BVMD	Cytosolic loop TM2–TM3	Yes	SNV5
c.653G>A	p.(Arg218His)	37	8.3%	ARB/BVMD	Cytosolic loop TM2–TM3	Yes	SNV5
c.689_691del	p.(Ile230del)	17	3.8%	BVMD	Cytosolic loop TM2–TM3	No	SNV5
c.692G>C	p.(Ser231Thr)	10	2.2%	BVMD	Cytosolic loop TM2–TM3	Yes	SNV5
c.903T>G	p.(Asp301Glu)	14	3.1%	AVMD/BVMD	Cytosolic C-termini	Yes	SNV5
c.905A>C	p.(Asp302Ala)	9	2.0%	BVMD	Cytosolic C-termini	Yes	SNV5
c.1669G>A	p.(Glu557Lys)	6	1.3%	ARB/BVMD/RP	Cytosolic C-termini	Yes	VUS

### Predicted Effect of the Most Frequent Novel French Variants

Among the 40 new *BEST1* variants in the French cohort, four are overrepresented: p.(Gly26Asp), p.(Val90Met), p.(Val137Met), and p.(Ile230del) (Tables 1, 2). They affect conserved regions in paralogs and orthologs of bestrophin (Supplementary Table S8). To support our in silico predictions and assess the structure–function relationship of our key variants, we examined functional assays reported in the literature investigating the impact of BEST1 variants on membrane trafficking, oligomerization, ion conductance activity, and intracellular calcium signaling (Supplementary Table S9).

#### p.(Gly26Asp)

**Figure 2.**
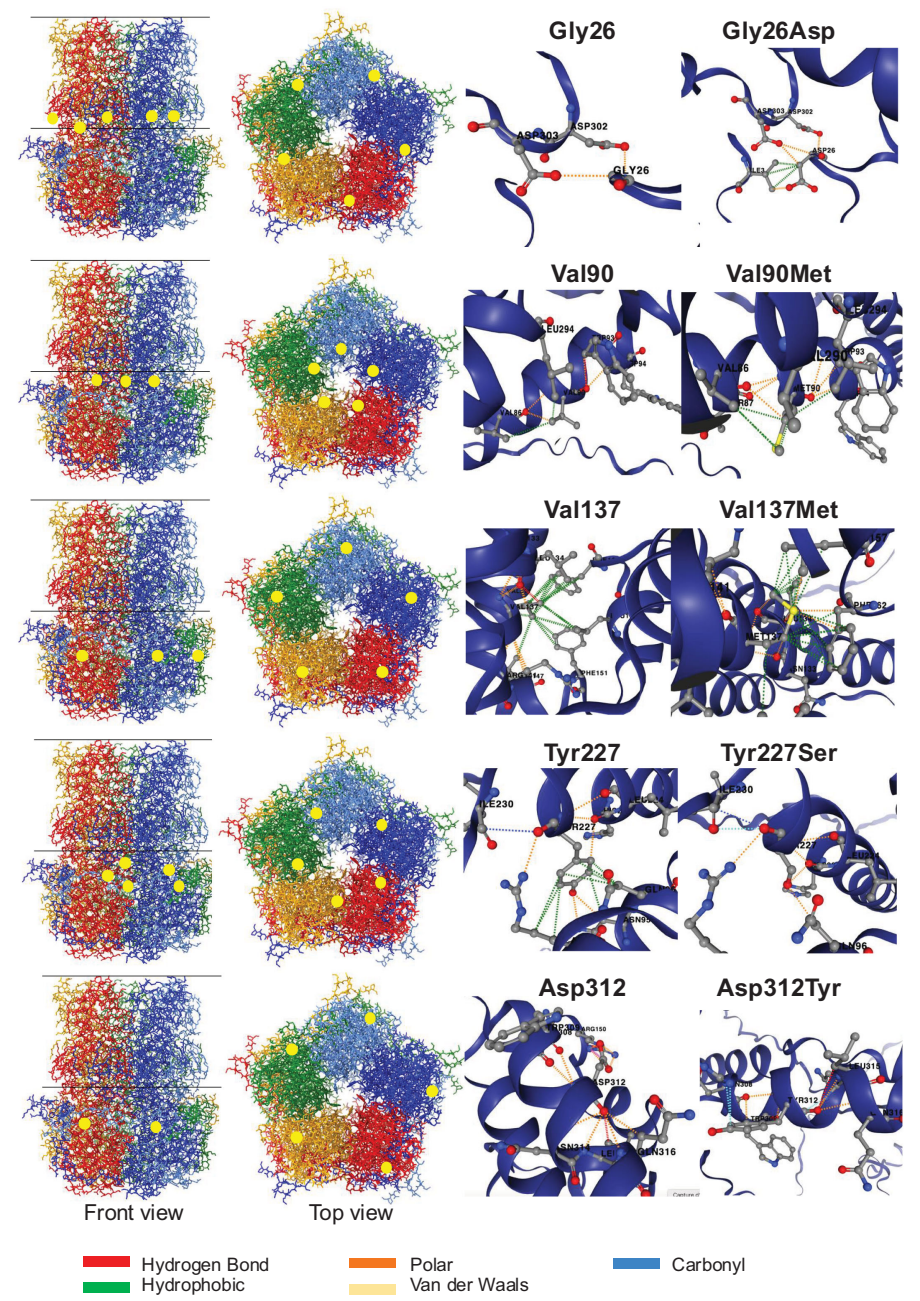
Novel French variants and 3D structure. The left shows the location of the variant in the protein, with a front view and top view, and amino acid interactions in normal and mutant versions (based on DynaMut2 software). *Yellow dots* represent the amino acid location in the 3D structure. The p.(Ile230del) variant could not be modeled by DynaMut2.

Gly26, in the cytosolic N-termini domain, forms a hydrogen bond with Asp302, which is a highly conserved residue in the Ca^2+^ clasping site and implicated in Ca^2+^ binding^17^ (Fig. 2; Supplementary Tables S8, S9). Despite maintaining the link between amino acids 26 and 302, the Gly-to-Asp substitution at position 26 may disrupt Ca^2+^ binding and clasping by replacing a small conserved amino acid with a larger, negatively charged one. The Ca^2+^ binding sites are highly conserved within the bestrophin family (Supplementary Table S8). Ca^2+^ coordination has pentagonal bipyramidal geometry, with an average Ca^2+^-oxygen distance of 2.5 Å.^18^ Changes in this area may modify this distance and alter the local concentration of Ca^2+^. The clinical examination of a patient harboring this variant is presented in Supplementary Figure S5.

#### p.(Val90Met)

Val90, situated in the TM2–TM3 loop near the channel neck or Ca^2+^ clasp site (Fig. 2), is affected by substitution with a larger residue (methionine), which may destabilize the Ca^2+^ clasp. Nearby variants, p.(Arg92Ser) and p.(Trp93Cys), showed reduced anion conductance and Ca^2+^-dependent current^19^^–^^22^ (Supplementary Table S9).

#### p.(Val137Met)

Val137 is located in the TM2–TM3 cytosolic loop and appears in the cytosolic inner cavity based on the 3D structure. This variant leads to significant steric hindrance (Fig. 2), in a region hosting many pathogenic variants (Supplementary Tables S2, S7). Given in cellulo tests, three nearby mutants, p.(Leu140Val), p.(Arg141His), and p.(Arg141Ser), caused intracellular mislocalization in Madin–Darby canine kidney type II (MDCK-II) cells; however, p.(Arg141His) remained basolateral in induced pluripotent stem cells (iPSCs) and RPE cells.^21^ All three variants affected Cl⁻ current in human embryonic kidney 293 (HEK293) cells without altering pentamer oligomerization (Supplementary Table S9).^15^^,^^21^^–^^24^ The p.(Leu140Val) variant is inherited dominantly like the p.(Val137Met) in our cohort, whereas p.(Arg141His) and p.(Arg141Ser) are recessive variants.

#### p.(Ile230del)

This variant is one of the most frequently identified in our cohort (17 patients). Ile230 is located in the TM2–TM3 cytosolic loop, near the cytosolic side. Although this amino acid is not highly conserved (Supplementary Table S8), its proximity to the Ca^2+^ clasp site cannot be ruled out. Ile230 is engaged in many hydrogen bonds, such as with Ala226 and Arg92, the latter being also linked to Tyr227 (Supplementary Fig. S6). As discussed above, Arg92 reduces anion conductance. The effect of Tyr227 is described below. One might hypothesize that deleting Ile230 would destabilizes the channel structure in this region, affecting anion conductance and possibly Ca^2+^ binding. The clinical examination of a representative patient harboring this variant is presented in Supplementary Figure S5.

#### p.(Tyr227Ser) and p.(Asp312Tyr)

Interestingly, we identified two variants altering the same highly conserved residue as previously functionally assessed mutants: p.(Tyr227Ser) and p.(Asp312Tyr) (Fig. 2, Supplementary Table S8). Tyr227 has been frequently studied in the literature, including the following variants: p.(Tyr227Asn), p.(Tyr227Cys), p.(Tyr227Phe), and p.(Tyr227Glu)^15^^,^^22^^,^^24^^–^^27^ (Supplementary Table S9). Different effects on basolateral membrane trafficking were observed according to the substitution, but the most frequently tested mutant, p.(Tyr227Asn), exhibited a significant effect on anion conductance. The Tyr227 residue seems to be of main interest as it forms a putative sorting motif with DWI and the phosphorylation of Tyr227 in p.(Tyr227Glu), altered basolateral localization compared to the nonphosphorylated form p.(Tyr227Phe).^27^ Due to the large distance between Tyr and Ser, we propose that our new variant is pathogenic and impacts Cl^−^ current.

Asp312 is well documented for its negative impact on Cl⁻ current and its association with dominant or recessive vitelliform macular dystrophies (Supplementary Table S9). The p.(Asp312Tyr) variant was found heterozygous in two patients with BVMD and considered pathogenic (Supplementary Fig. S5).

### Functional Consequences of Variants and Phenotypes

In the LOVD, dominant vitelliform macular dystrophies (AVMD and BVMD) are predominantly associated with missense variants (66.6% and 96.1%, respectively) (Supplementary Table S6A), and loss-of-function variants account for 33.3% in AVMD and 3.9% in BVMD. Conversely, recessive forms show a higher prevalence of loss-of-function variants (43.8%; *P* < 0.0001). Based on a review of the different functional studies performed for *BEST1* variants (Supplementary Table S9), we established a correlation between the phenotype and the protein function and identified associated clinical features (Supplementary Fig. S7).

#### Best Vitelliform Macular Dystrophy

Forty-two percent of *BEST1* variations in the LOVD were reported in BVMDs (Supplementary Table S6A). These variants are mainly located in the TM2–TM3 loop and the C- and N-terminal domains in the LOVD and French cohorts (Supplementary Table S5). The underlying mechanism of BVMD may vary between mutants; however, the common feature is the loss of ion channel activity. Many variants induce reduced or abolished membrane currents when co-expressed with wild-type BEST1 protein in HEK293 cells.^20^^,^^23^^,^^28^ Nachtigal et al.^29^ proposed two molecular pathways: In the first, the variant leads to an unstable protein, able to oligomerize but, in unstable channel complexes, mislocalizes to intracellular compartments and is degraded by lysosomes. The second mechanism involves mutants that are correctly localized to the plasma membrane but negatively affect the closed conformation of a channel, impairing anion permeability (Supplementary Table S9). In summary, BVMD is due to the dominant negative effect of *BEST1* variants, leading to a reduction in the anion transport function.

#### Adult-Onset Vitelliform Macular Dystrophy

Most of the AVMD variants overlap with BVMD or ARB. AVMD is typically known to present with a smaller vitelliform lesion and subnormal to normal electrooculography.^30^ The age of AVMD onset is highly variable, but patients tend to remain asymptomatic until their fifth decade.^31^ As shown in BVMD, variants can be either correctly or incorrectly localized to the plasma membrane^20^^,^^24^ and alter ionic conductance. Functional analyses tend to support the hypothesis that AVMD may represent a milder BVMD form. For example, studies have performed surface biotinylation assays and patch clamp experiments in transfected HEK293T cells. The tested *BEST1* variants, p.(Ile38Ser)^32^ and p.(Ala243Val),^33^ did not affect the membrane expression but showed significantly smaller currents than the wild type and larger currents than other *BEST1* variants, causing autosomal dominant BVMD or ARB. Moreover, unlike other *BEST1* variations with dominant-negative effects on wild-type channels, p.(Ala243Val) did not influence the wild-type current when co-transfected with the wild type, which is consistent with the milder symptoms associated with this variant.^33^

#### Autosomal Recessive Bestrophinopathy

*BEST1* variants associated with autosomal recessive forms were predominantly located in the TM2–TM3 cytosolic loop in both cohorts (Supplementary Table S5A). ARB was initially considered as rare, but its frequency is now estimated at between 2.5% and 3.4%.^34^ To date, in the LOVD, 162 different variations have been reported in ARB and 32 have been described in multiple phenotypes (Supplementary Tables S2, S6A). The variants are mainly missense (56.2%), although a significant proportion is truncating (43.8%), which contrasts with other phenotypes associated with *BEST1*. This reflects a different pathogenic mechanism than that described above for dominant forms. Indeed, ARB either is the “null phenotype” with no functional protein in the RPE^9^ or is due to synthesis of unstable proteins, degraded by the endoplasmic reticulum (ER)-associated proteasomal machinery, contributing to ER stress.^29^

Whole-cell patch-clamp methods have shown that certain ARB mutants, when expressed alone or co-expressed with another pathogenic variant, strongly reduce Cl^−^ channel activity in HEK293 cells, whereas activity remains unaltered when the mutants are co-expressed with the wild-type protein.^9^^,^^23^ A threshold effect has been suggested to explain the recessive phenotype: The disease is only expressed when functional protein activity is below a certain level.^9^ ARB variants alone cannot cause structural changes affecting protein activity. This hypothesis has been confirmed by studies on two truncating variants with premature stop codon and for which activation of the nonsense-mediated decay (NMD) mechanism was suspected: c.521_522del and c.1100+1G>A.^35^ RNA transcript quantification revealed low *BEST1* expression (13% and 22%, respectively) for the two variants. This is consistent with clinical severity and inversely correlates with residual transcript quantity. In the simple heterozygous state, the residual activity provided by the normal allele is sufficient to ensure a functional protein despite the expression defect linked to these ARB variants. This is unlike variants with a dominant-negative effect which alone impair protein function. To conclude, *BEST1* variants lead to either a lack of protein synthesis or to an unstable protein degraded by the proteasome machinery. This results in a decrease in anion conductance. Haploinsufficiency is therefore the mechanism involved in ARB. The reason why certain variations are associated with both dominant and recessive phenotypes remains unknown. The existence of modifying factors should be considered. Indeed, a sex imbalance in favor of men (a proportion of 38% women; 95% confidence interval, 29%–48%; *P* = 0.015) was demonstrated specifically in autosomal dominant *BEST1* disease but not in the autosomal recessive form.^36^ Additionally, variable expressivity and incomplete penetrance are observed in bestrophinopathies, although they are poorly reported.^37^^–^^40^ However, no clear genetic or environmental factors have been identified to date.

#### Autosomal Dominant Vitreoretinochoroidopathy

There are few reported cases of ADVIRC (24 in the LOVD, 14 in the French cohort) (Supplementary Tables S2, S7). Most of the associated *BEST1* variants are missense—p.(Gly83Asp), p.(Val86Met), p.(Val235Ala), p.(Tyr236Cys), p.(Val239Met), and p.(Met571Thr)—and located predominantly in the TM2–TM3 loop (Supplementary Table S5). In vitro functional assays have shown altered pre-mRNA splicing, causing exon skipping. These variants affect splicing by altering the binding of spliceosomal serine–arginine (SR) proteins to exonic splicing enhancer (ESE) or exonic splicing silencer (ESS) sites. This results in the skipping of exon 4, 6, or 7 or the duplication of exon 6.^9^^,^^14^ However, two human-induced pluripotent stem cell (hiPSC)-based studies invalidated these results for p.(Val86Met) and p.(Val235Ala).^29^^,^^41^ Instead, a splice defect was confirmed in vivo for the c.1101-1G>T variant affecting the canonical splice acceptor site of intron 9. The patient's leukocyte RNA study showed exon 10 skipping, causing the loss of 213 amino acids, p.(Ser367_Asn579del).^42^ The proposed mechanism is that the mutants are correctly localized on the plasma membrane and negatively affect the closed state of the channel, significantly increasing the anion permeability.^17^^,^^29^ In summary, ADVIRC variants are responsible for increased anion permeability, unlike previous phenotypes.

#### Retinitis Pigmentosa

RP-associated *BEST1* variants, including p.(Ile205Thr), p.(Tyr227Cys), and p.(Asp228Asn),^43^ are thought to induce splicing defects and are all localized in the TM2–TM3 cytosolic loop. RP and ADVIRC share common features (localization, peripheral retinal involvement, and retinal pigment abnormalities). RP variants may represent true ADVIRCs if they exhibit similar mechanisms.

## Discussion

The LOVD includes 488 different *BEST1* variants identified in 2205 patients, and the French cohort has 150 variants in 450 patients. Both populations are homogeneous regarding variant type (mostly missense), distribution of variants in the gene (mainly located in the TM2–TM3 loop corresponding to the large cytosolic part of the channel, including the internal cavity, Ca^2+^-binding sites, and the second restriction site), and associated phenotypes. Thus, BVMDs are the most frequent (41.8% in the LOVD and 58.7% in the French cohort), followed by ARBs (33.2% and 27.3% respectively).

However, some differences are observed between the French and LOVD cohorts, in the protein distribution of the variant types and in the frequency of certain. Such points may be influenced by several population-specific factors. The LOVD cohort is comprised of data from multiple countries and ethnic backgrounds, making it genetically more heterogeneous. In contrast, the French cohort reflects a more homogeneous population in terms of geography and genetics, in which regional founder effects may exist. This local enrichment is highlighted by the near absence of recurrent French variants in the LOVD. Furthermore, the higher proportion of novel variants in the French dataset (26.7%) suggests that certain populations may be underrepresented in public databases. Indeed, disparities in variant documentation reflect differences in access to genetic testing, clinical diagnostic criteria, and variant reporting practices across countries. This may explain why the LOVD cohort is comprised of data predominantly from European, Middle Eastern (particularly Israeli), North American, and East Asian (Chinese, South Korean, and Japanese) populations. Variants from other regions may be underrepresented, particularly if they are only reported at a national level or published in languages other than English, which limits their inclusion in international databases. These observations emphasize the importance of developing and maintaining population-specific variant registries, as well as the need for broader international data sharing, to achieve a more comprehensive understanding of the *BEST1* mutational landscape and its phenotypic consequences.

Reclassification according to ACMG pathogenicity criteria identified a majority of variants as pathogenic or probably pathogenic, with a small proportion remaining as VUSs (7.4% in the LOVD and 5.3% in the French cohort). Particular attention was given to VUSs based on their location in the channel (according to UCSF ChimeraX and DynaMut2), their involvement in a major functional site, and their proximity to functionally tested variants, in addition to in silico prediction scores. Some variants with low prediction scores, located after amino acid 377 in the unstructured region of the protein or variants with insufficient clinically descriptions, remained as VUSs. Two variants described in the LOVD, p.(Ser7Asn) and p.(Ser209Asn), classified as VUSs deserve attention. Both are predicted by in silico software to be “low missense.” The p.(Ser209Asn) is localized at the extracellular pore entrance, near p.(Ile205Thr), which has been linked to reduced Cl^−^ channel activity.^15^^,^^17^^,^^24^ Similarly, p.(Ser7Asn) is located intracellularly, below the neck, alongside p.(Thr6Pro), a variant known to alter channel permeability.^22^^,^^24^^,^^26^ These controversial variants require further identification in additional patients for reclassification. Our study aimed to perform an in silico analysis of the possible effect of VUS on ionic conductance. However, only functional studies such as patch clamp experiments on transfected HEK293T cells could determine their possible deleterious effects.

Structural analysis suggests that missense variants located downstream of amino acid 377 are likely not pathogenic, as this region of the cytosolic C-terminal domain is predicted to be unstructured (Supplementary Tables S2, S7). Although it may be involved in BEST1 channel regulation,^44^ no functional data currently support the pathogenicity of variants in this region. This is demonstrated by the lack of effect observed for three functionally tested variants: p.(Leu472Profs*10), p.(His490Glnfs*24), and p.(Leu567Phe)^24^^,^^28^ (Supplementary Table S9). However, the effect could be indirect, as the proline-rich motif between amino acids 468 and 486 has been shown to interact with the β-subunits that regulate the voltage-dependent L-type Ca^2+^ channels.^26^ Several truncating variants in this region possibly activate NMD, resulting in the degradation of *BEST1* transcripts and the absence of protein synthesis. Some have been reported in ARB cases, supporting the loss-of-function mechanism.

Only a few large *BEST1* gene deletions have been reported in both cohorts, with no large duplications observed. These large deletions involve either exons 1 and 2 or exon 2 alone, as observed in the French cohort. Analysis using the UCSC Genome browser (https://genome.ucsc.edu/) showed that introns 1 and 2 of *BEST1* are particularly rich in short interspersed nuclear element (SINE) repeats (Alu or mammalian interspersed repeat [MIR]). This could explain homologous recombination and the generation of these large deletions. However, these repeated sequences are also present in other introns, suggesting that additional deletions are possible and warrant investigation. The frequency of these large rearrangements may have been underestimated by sequencing methods. In our laboratory, the search for CNVs was systematically performed in our screening process, initially by MLPA and then by NGS data, and our results were then confirmed with quantitative PCR. We therefore believe that large *BEST1* rearrangements are rare and not a common pathological mechanism.

## Conclusions

Our study updated the curation of the LOVD *BEST1* database and described the largest French *BEST1* cohort. It also classified all reported variants using the ACMG guidelines, 40 of which were novel. This study employed in silico data and tools, including predictive software, frequency data, and the localization and functional impact of tested variants on channel conductance. Studying the 3D conformation of the channel is a valuable visual tool for understanding the changes in chemical interactions caused by variants. These in silico approaches are useful given that functional studies on patient-derived cells, such as induced pluripotent stem cells and organoids, remain costly, time consuming, and tedious. However, they are still the only way to confirm a deleterious effect. Many variants remain to be studied. Our findings provide a practical framework for correlating genotype, structure, function, and phenotype, enabling appropriate genetic counseling for patients and their families.

## Supplementary Material

Supplement 1

Supplement 2

Supplement 3

Supplement 4

Supplement 5

Supplement 6

Supplement 7

Supplement 8

Supplement 9

Supplement 10

Supplement 11

Supplement 12

Supplement 13

Supplement 14

Supplement 15

Supplement 16

Supplement 17

Supplement 18
