# Supplementary material for: Novel BEST1 Variant Characterization in a Large French Cohort in Light of Updated Bestrophin-1 Structure–Function Correlation
Source: Invest Ophthalmol Vis Sci. 2025 Sep 2;66(12):4. doi: 10.1167/iovs.66.12.4 (PMC12410269; doi:10.1167/iovs.66.12.4)
Supplement: Supplement 1 [file iovs-66-12-4_s001.pdf]

Supplementary Figure S1: Pie-charts of variants distribution by ACMG value or by variant type. A, for LOVD; B, for the French cohort

A.

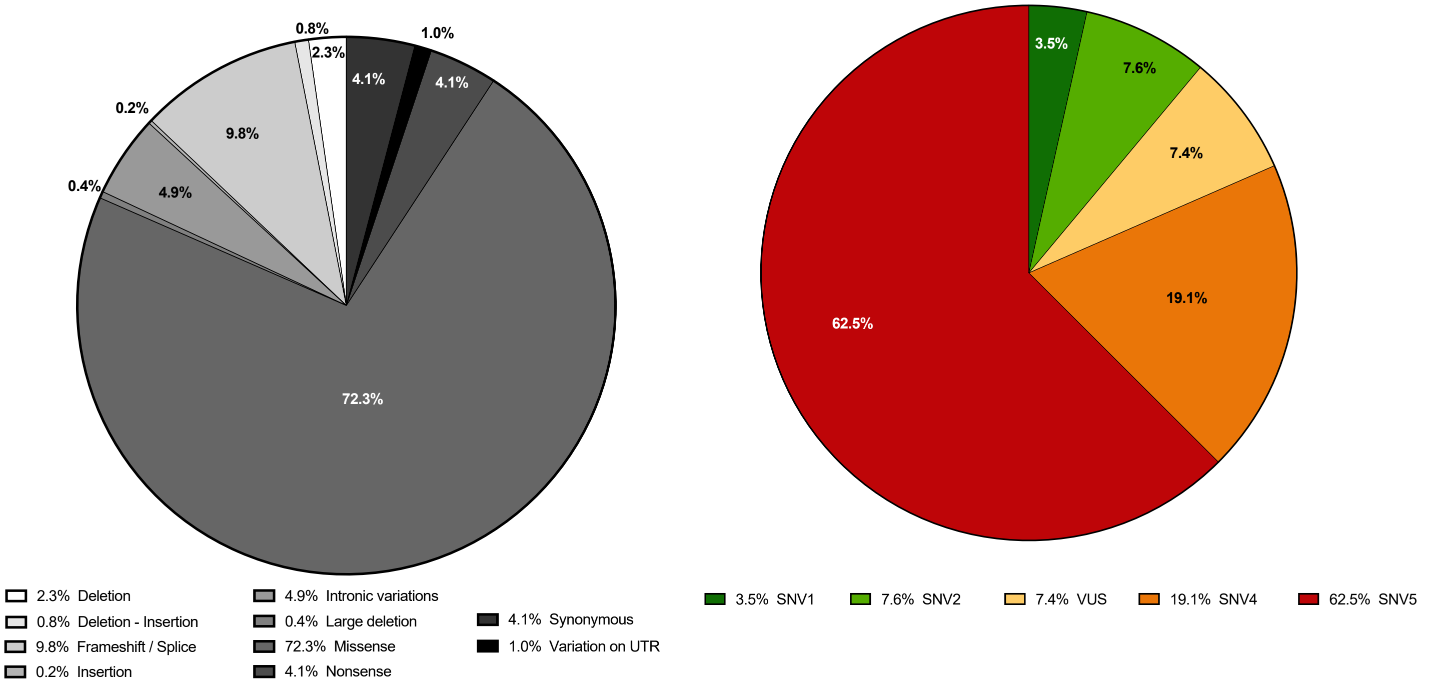

n (total of variations) = 488

B.

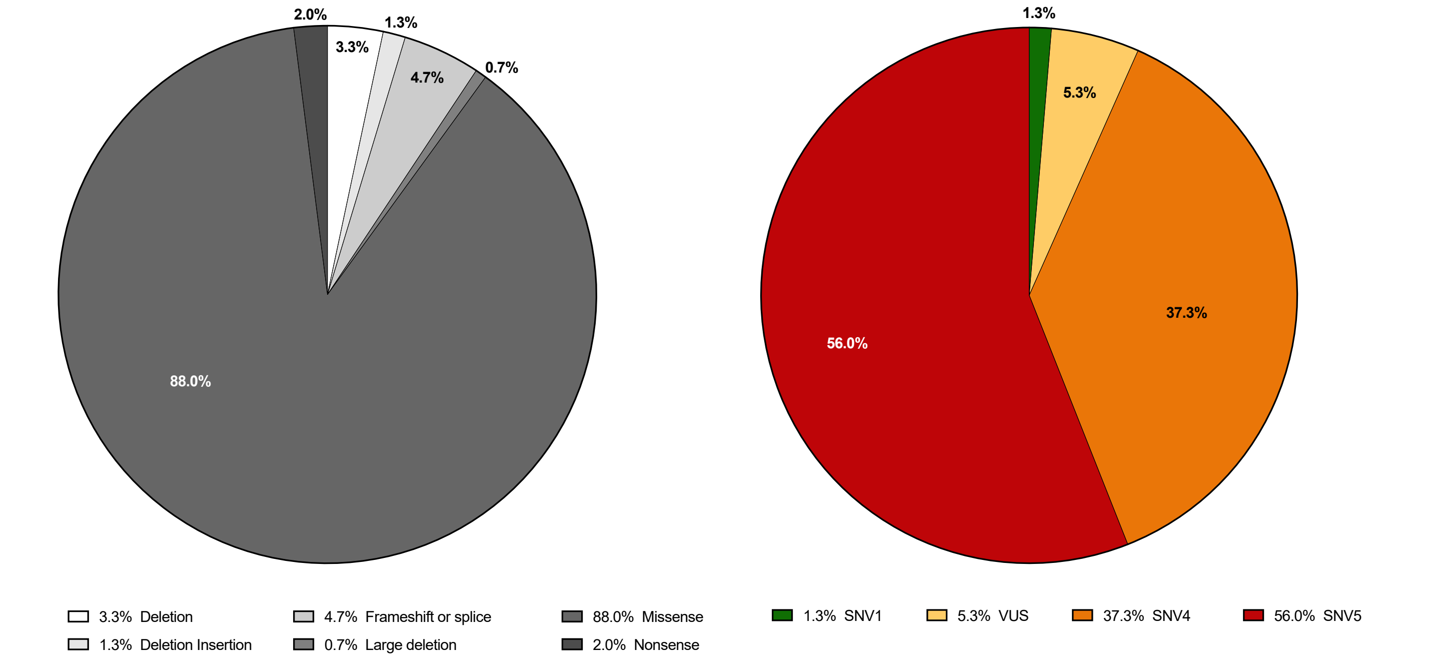

n (total of variations) = 150
