# Supplementary material for: Novel BEST1 Variant Characterization in a Large French Cohort in Light of Updated Bestrophin-1 Structure–Function Correlation
Source: Invest Ophthalmol Vis Sci. 2025 Sep 2;66(12):4. doi: 10.1167/iovs.66.12.4 (PMC12410269; doi:10.1167/iovs.66.12.4)
Supplement: Supplement 2 [file iovs-66-12-4_s002.pdf]

Cytosolic N termini domain (aa 1-31)

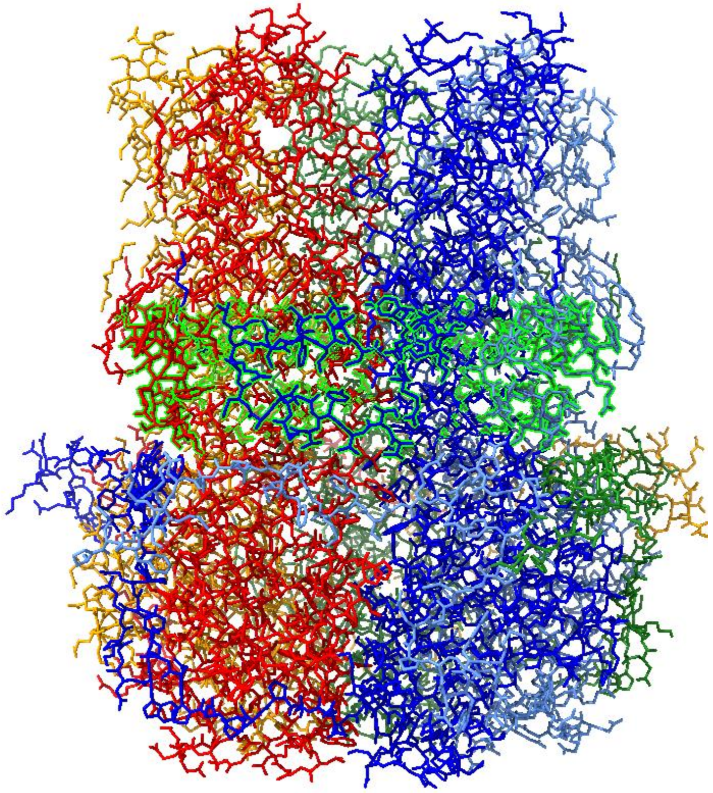

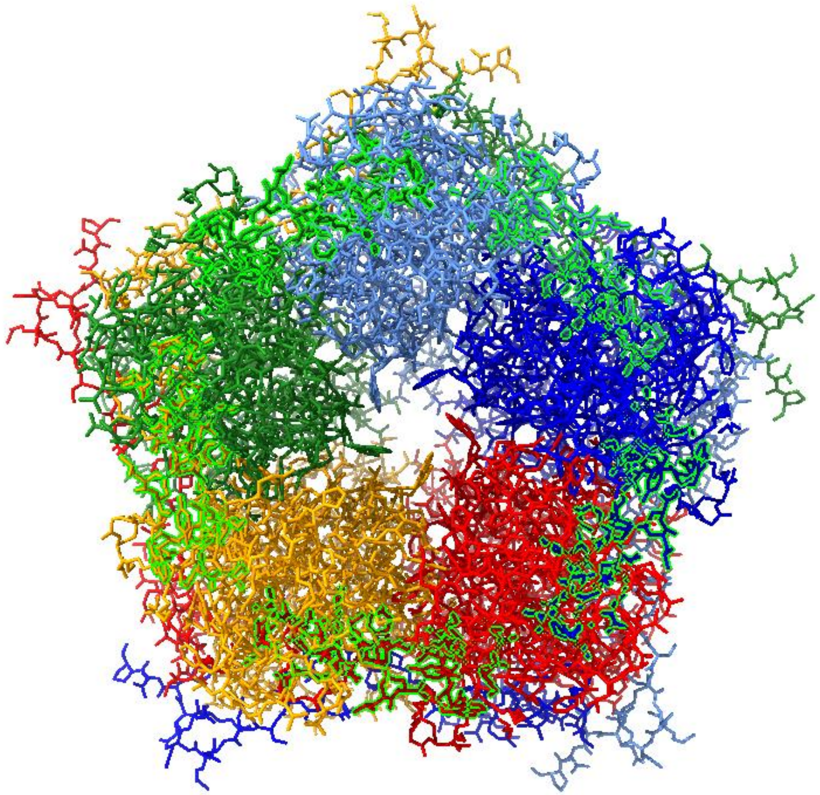

```
1 MTITYTSQVANARLGSFSRLLLCWRGSIYKLLYGE
36 FLIFLLCYYIIRFIYRLALTEEQQLMFEKLTLYCD
71 SYIQLIPISFVLGFYVTLVVTRWWNQYENLPWPDR
106 LMSLVSGFVEGKDEQGRLLRRTLIRYANLGNVLIL
141 RSVSTAVYKRFPSAQHLVQAGFMTPAEHKQLEKLS
176 LPHNMFWVPWVWFANLSMKAWLGGRIRDPILLQSL
211 LNEMNTLRTQCGHLYAYDWISIPLVYTQVVTVAVY
246 SFFLTCLVGRQFLNPAKAYPGHELDLVVPVFTFLQ
281 FFFYVGWLKVAEQLINPFGEDDDDFETNWIVDRNL
316 QVSLLAVDEMHQDLPRMEPDMYWNKPEPQPPYTAA
351 SAQFRRASFMGSTFNISLNKEEMEFQPNQEDEEDA
386 HAGIIGRFLGLQSHDHHPPRANSRTKLLWPKRESL
421 LHEGLPKNHKAAKQNVRGQEDNKAWKLKAVDAFKS
456 APLYQRPGYYSAPQTPLSPTPMFFPLEPSAPSKLH
491 SVTGIDTKDKSLKTVSSGAKKSFELLSESDGALME
526 HPEVSQVRRKTVEFNLTDMPEIPENHLKEPLEQSP
561 TNIHTTLKDHMDPYWALENRDEAHS
```

TM1 domain (aa 32-51)

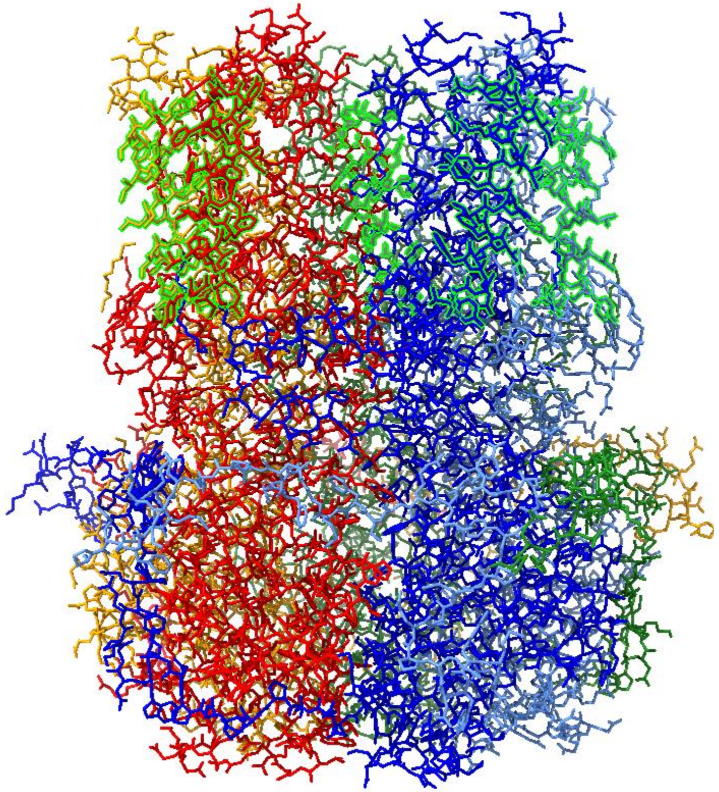

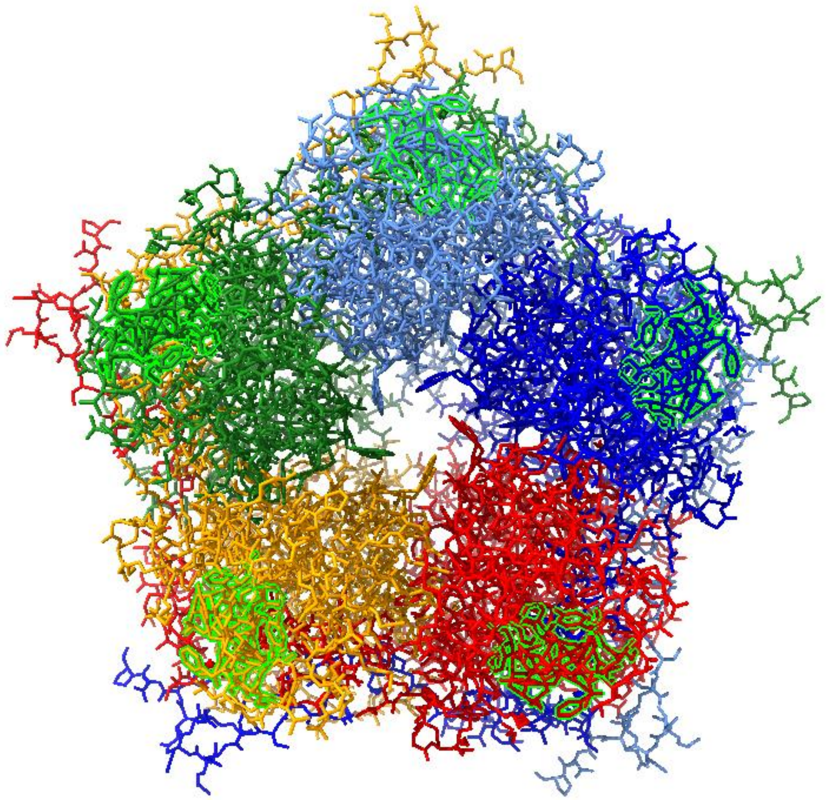

1 MTITYTSQVANARLGSFSRLLLCWRGSIYKLLYGE
36 FLIFLLCYYIIRFIYRLALTEEQQLMFEKLTLYCD
71 SYIQLIPISFVLGFYVTLVVTRWWNQYENLPWPDR
106 LMSLVSGFVEGKDEQGRLLRRTLIRYANLGNVLIL
141 RSVSTAVYKRFPSAQHLVQAGFMTPAEHKQLEKLS
176 LPHNMFWVPWVWFANLSMKAWLGGRIRDPILLQSL
211 LNEMNTLRTQCGHLYAYDWISIPLVYTQVVTVAVY
246 SFFLTCLVGRQFLNPAKAYPGHELDLVVPVFTFLQ
281 FFFYVGWLKVAEQLINPFGEDDDDFETNWIVDRNL
316 QVSLLAVDEMHQDLPRMEPDMYWNKPEPQPPYTAA
351 SAQFRRASFMGSTFNISLNKEEMEFQPNQEDEEDA
386 HAGIIGRFLGLQSHDHHPPRANSRTKLLWPKRESL
421 LHEGLPKNHKAAKQNVRGQEDNKAWKLKAVDAFKS
456 APLYQRPGYYSAPQTPLSPTPMFFPLEPSAPSKLH
491 SVTGIDTKDKSLKTVSSGAKKSFELLSESDGALME
526 HPEVSQVRRKTVEFNLTDMPEIPENHLKEPLEQSP
561 TNIHTTLKDHMDPYWALENRDEAHS

Extracellular loop TM1-TM2 domain (aa 52-60)

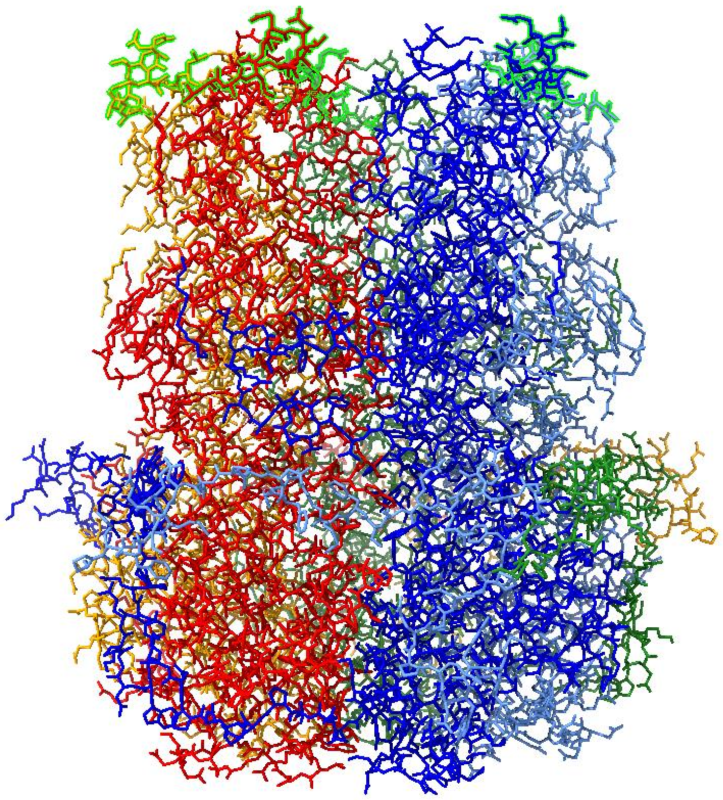

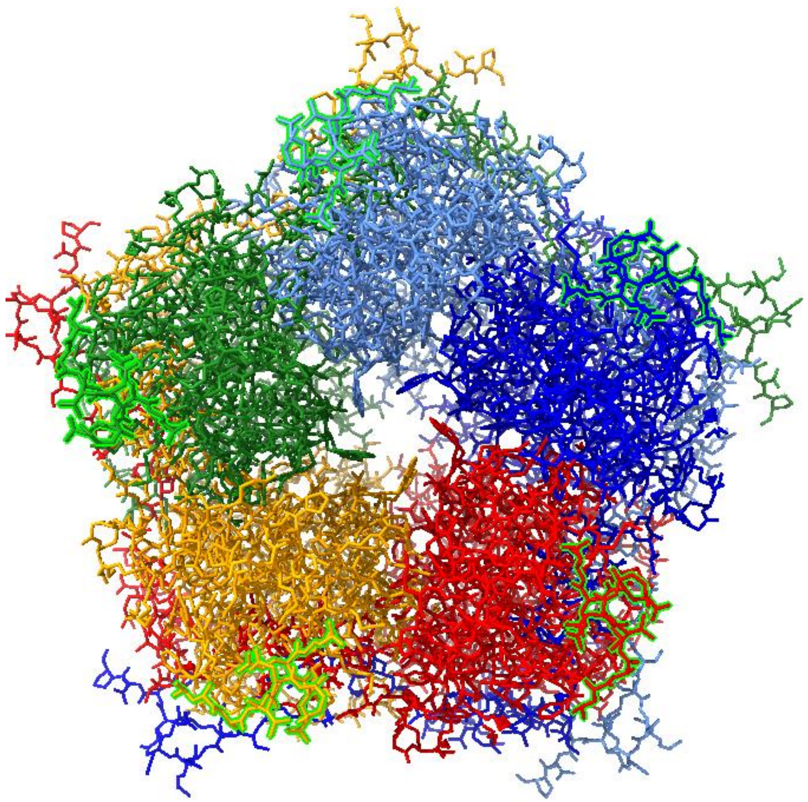

```
1   MTITYTSQVANARLGSFSRLLLCWRGSIYKLLYGE
36  FLIFLLCYYIIRFIYRLALTEEQQLMFEKLTLYCD
71  SYIQLIPISFVLGFYVTLVVTRWWNQYENLPWPDR
106 LMSLVSGFVEGKDEQGRLLRRTLIRYANLGNVLIL
141 RSVSTAVYKRFPSAQHLVQAGFMTPAEHKQLEKLS
176 LPHNMFWVPWVWFANLSMKAWLGGRIRDPILLQSL
211 LNEMNTLRTQCGHLYAYDWISIPLVYTQVVTVAVY
246 SFFLTCLVGRQFLNPAKAYPGHELDLVVPVFTFLQ
281 FFFYVGWLKVAEQLINPFGEDDDDFETNWIVDRNL
316 QVSLLAVDEMHQDLPRMEPDMYWNKPEPQPPYTAA
351 SAQFRRASFMGSTFNISLNKEEMEFQPNQEDEEDA
386 HAGIIGRFLGLQSHDHHPPRANSRTKLLWPKRESL
421 LHEGLPKNHKAAKQNVRGQEDNKAWKLKAVDAFKS
456 APLYQRPGYYSAPQTPLSPTPMFFPLEPSAPSKLH
491 SVTGIDTKDKSLKTVSSGAKKSFELLSESDGALME
526 HPEVSQVRRKTVEFNLTDMPEIPENHLKEPLEQSP
561 TNIHTTLKDHMDPYWALENRDEAHS
```

TM2 domain (aa 61-82)

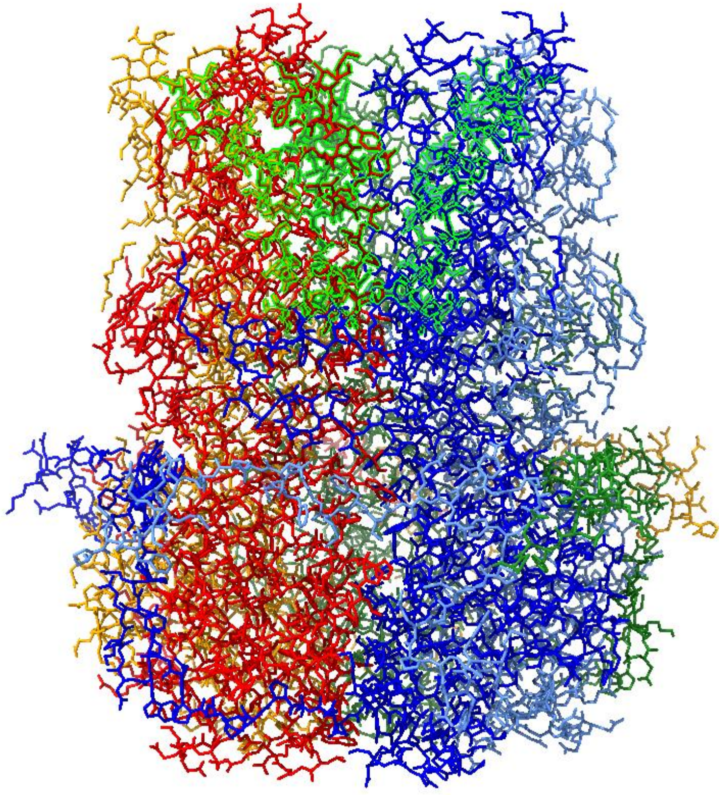

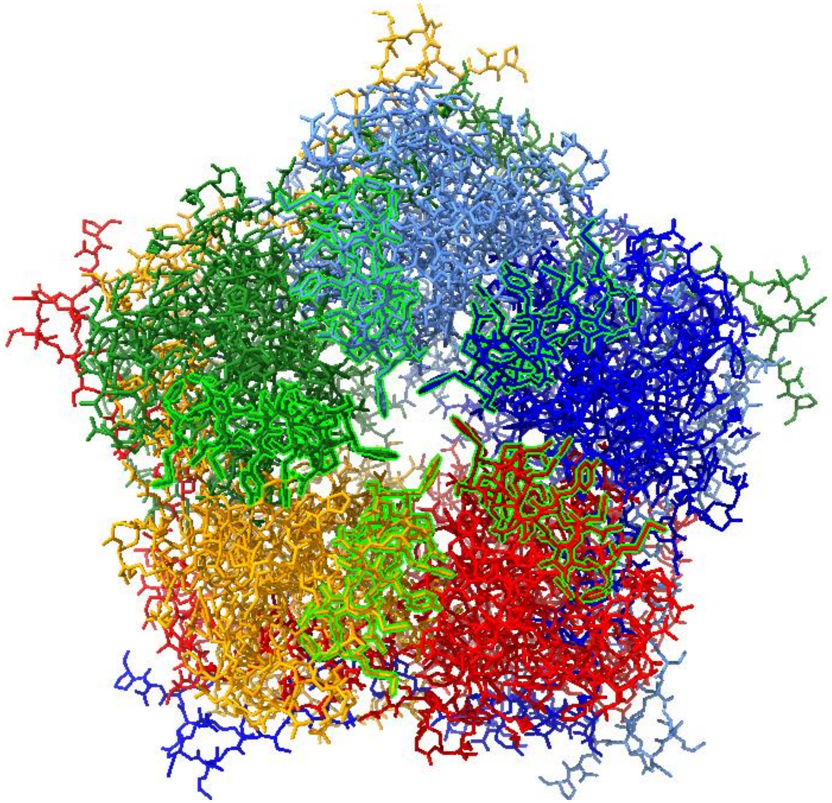

```
1   MTITYTSQVANARLGSFSRLLLCWRGSIYKLLYGE
36  FLIFLLCYYIIRFIYRLALTEEQQLMFEKLTLYCD
71  SYIQLIPISFVLGFYVTLVVTRWWNQYENLPWPDR
106 LMSLVSGFVEGKDEQGRLLRRTLIRYANLGNVLIL
141 RSVSTAVYKRFPSAQHLVQAGFMTPAEHKQLEKLS
176 LPHNMFWVPWVWFANLSMKAWLGGRIRDPILLQSL
211 LNEMNTLRTQCGHLYAYDWISIPLVYTQVVTVAVY
246 SFFLTCLVGRQFLNPAKAYPGHELDLVVPVFTFLQ
281 FFFYVGWLKVAEQLINPFGEDDDDFETNWIVDRNL
316 QVSLLAVDEMHQDLPRMEPDMYWNKPEPQPPYTAA
351 SAQFRRASFMGSTFNISLNKEEMEFQPNQEDEEDA
386 HAGIIGRFLGLQSHDHHPPRANSRTKLLWPKRESL
421 LHEGLPKNHKAAKQNVRGQEDNKAWKLKAVDAFKS
456 APLYQRPGYYSAPQTPLSPTPMFFPLEPSAPSKLH
491 SVTGIDTKDKSLKTVSSGAKKSFELLSESDGALME
526 HPEVSQVRRKTVEFNLTDMPEIPENHLKEPLEQSP
561 TNIHTTLKDHMDPYWALENRDEAHS
```

Cytosolic loop TM2-TM3 domain (aa 83-237)

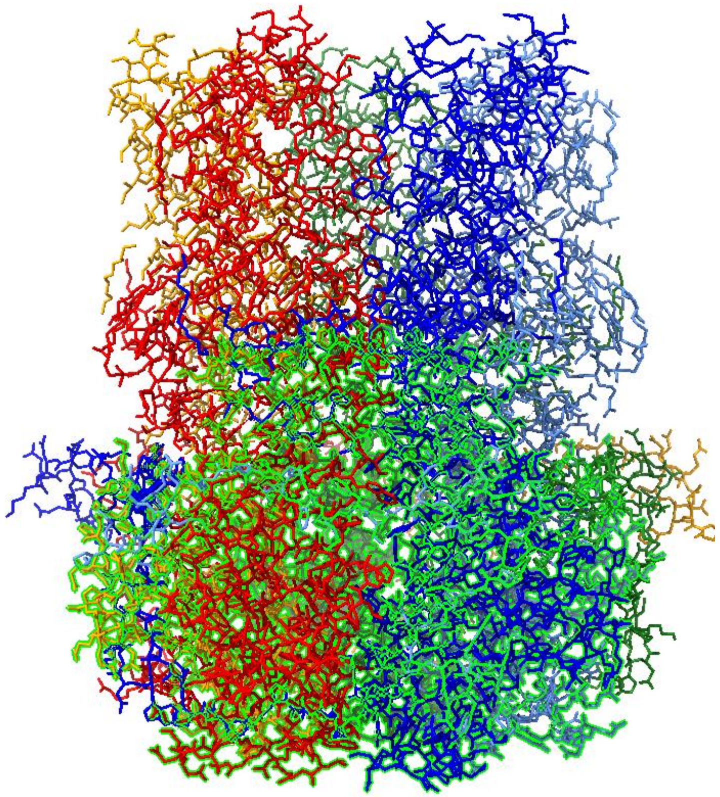

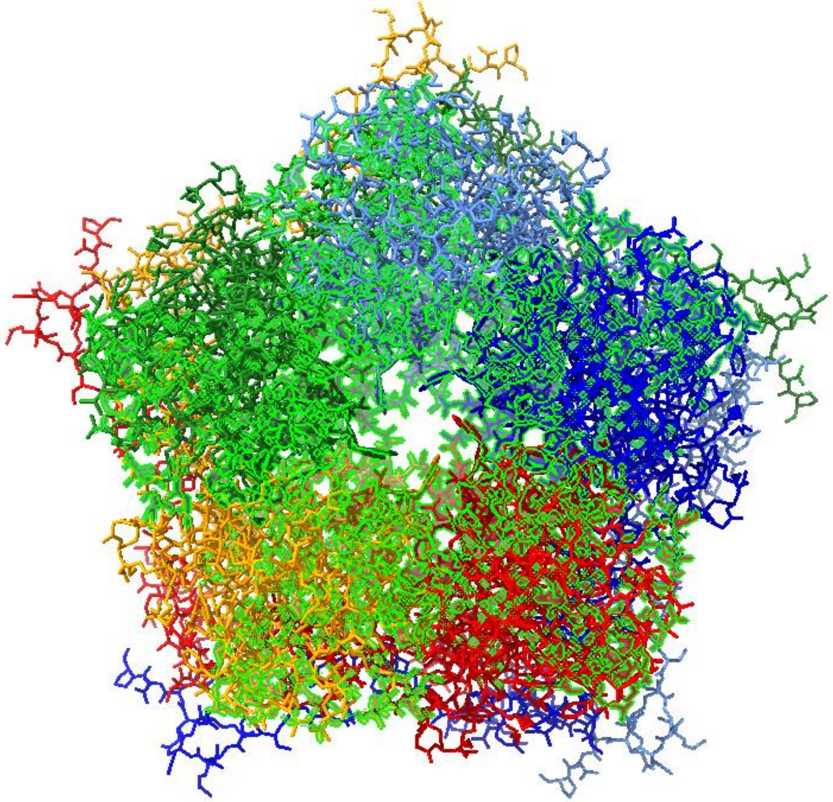

```
  1 MTITYTSQVANARLGSFSRLLLCWRGSIYKLLYGE
 36 FLIFLLCYYIIRFIYRLALTEEQQLMFEKLTLYCD
 71 SYIQLIPISFVLGFYVTLVVTRWWNQYENLPWPDR
106 LMSLVSGFVEGKDEQGRLLRRTLIRYANLGNVLIL
141 RSVSTAVYKRFPSAQHLVQAGFMTPAEHKQLEKLS
176 LPHNMFWVPWVWFANLSMKAWLGGRIRDPILLQSL
211 LNEMNTLRTQCGHLYAYDWISIPLVYTQVVTVAVY
246 SFFLTCLVGRQFLNPAKAYPGHELDLVVPVFTFLQ
281 FFFYVGWLKVAEQLINPFGEDDDDFETNWIVDRNL
316 QVSLLAVDEMHQDLPRMEPDMYWNKPEPQPPYTAA
351 SAQFRRASFMGSTFNISLNKEEMEFQPNQEDEEDA
386 HAGIIGRFLGLQSHDHHPPRANSRTKLLWPKRESL
421 LHEGLPKNHKAAKQNVRGQEDNKAWKLKAVDAFKS
456 APLYQRPGYYSAPQTPLSPTPMFFPLEPSAPSKLH
491 SVTGIDTKDKSLKTVSSGAKKSFELLSESDGALME
526 HPEVSQVRRKTVEFNLTDMPEIPENHLKEPLEQSP
561 TNIHTTLKDHMDPYWALENRDEAHS
```

TM3 domain (aa 238-255)

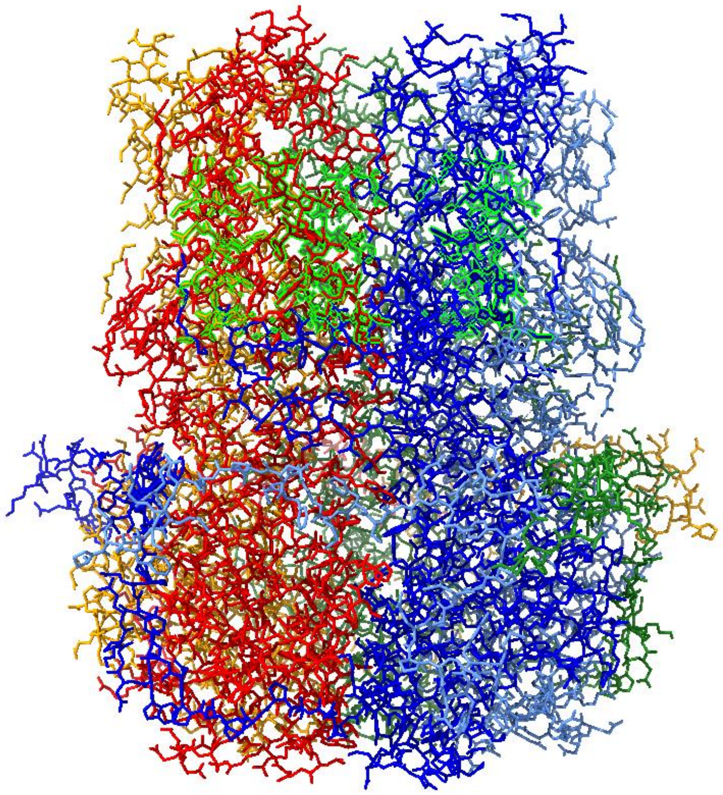

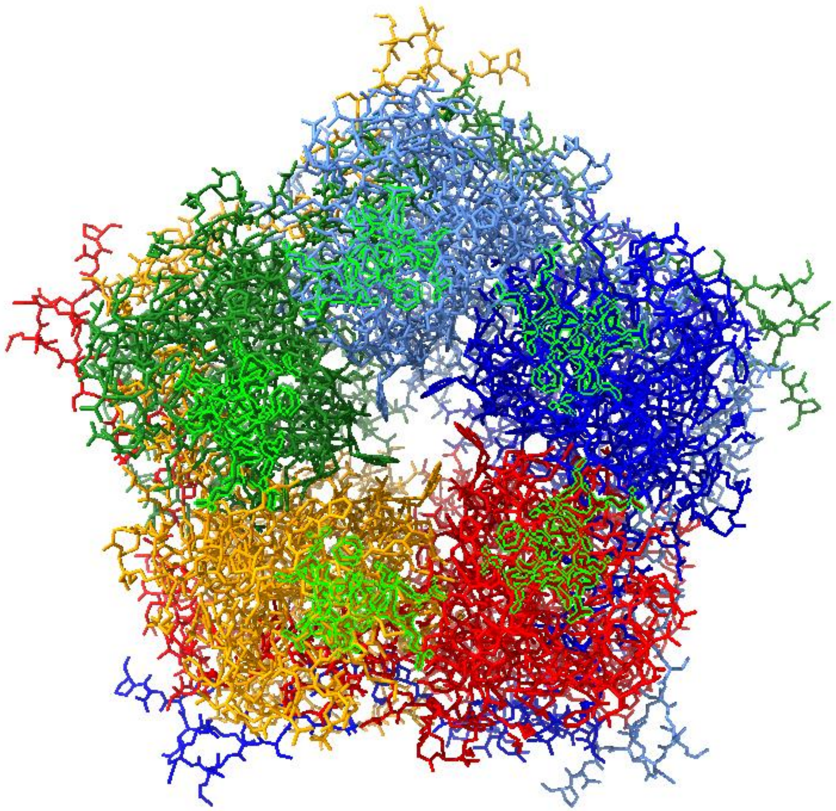

```
1   MTITYTSQVANARLGSFSRLLLCWRGSIYKLLYGE
36  FLIFLLCYYIIRFIYRLALTEEQQLMFEKLTLYCD
71  SYIQLIPISFVLGFYVTLVVTRWWNQYENLPWPDR
106 LMSLVSGFVEGKDEQGRLLRRTLIRYANLGNVLIL
141 RSVSTAVYKRFPSAQHLVQAGFMTPAEHKQLEKLS
176 LPHNMFWVPWVWFANLSMKAWLGGRIRDPILLQSL
211 LNEMNTLRTQCGHLYAYDWISIPLVYTQVVTVAVY
246 SFFLTCLVGRQFLNPAKAYPGHELDLVVPVFTFLQ
281 FFFYVGWLKVAEQLINPFGEDDDDFETNWIVDRNL
316 QVSLLAVDEMHQDLPRMEPDMYWNKPEPQPPYTAA
351 SAQFRRASFMGSTFNISLNKEEMEFQPNQEDEEDA
386 HAGIIGRFLGLQSHDHHPPRANSRTKLLWPKRESL
421 LHEGLPKNHKAAKQNVRGQEDNKAWKLKAVDAFKS
456 APLYQRPGYYSAPQTPLSPTPMFFPLEPSAPSKLH
491 SVTGIDTKDKSLKTVSSGAKKSFELLSESDGALME
526 HPEVSQVRRKTVEFNLTDMPEIPENHLKEPLEQSP
561 TNIHTTLKDHMDPYWALENRDEAHS
```

Extracellular loop TM3-TM4 domain (aa 256-274)

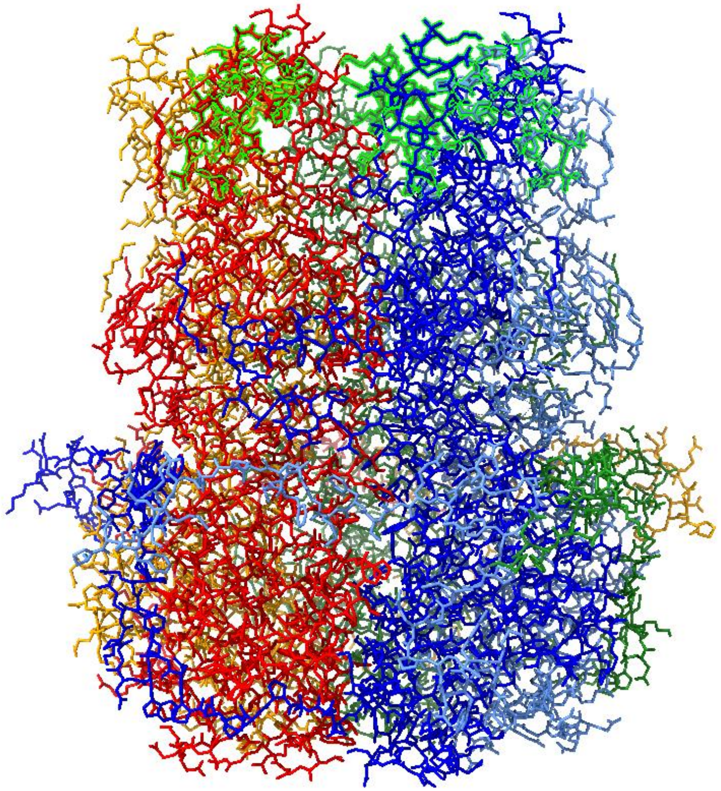

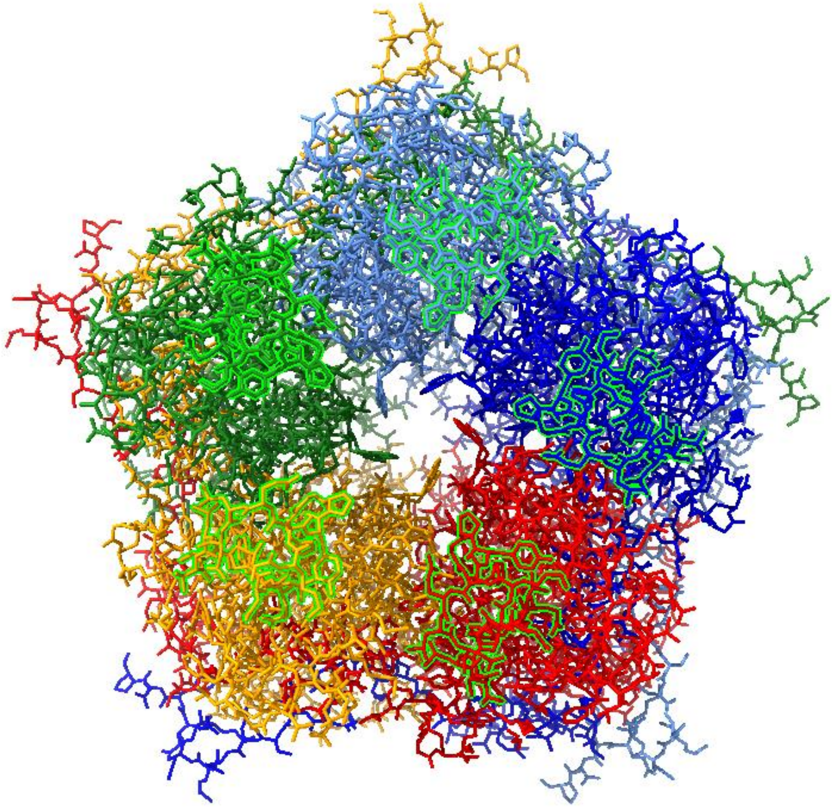

1 MTITYTSQVANARLGSFSRLLLCWRGSIYKLLYGE
36 FLIFLLCYYIIRFIYRLALTEEQQLMFEKLTLYCD
71 SYIQLIPISFVLGFYVTLVVTRWWNQYENLPWPDR
106 LMSLVSGFVEGKDEQGRLLRRTLIRYANLGNVLIL
141 RSVSTAVYKRFPSAQHLVQAGFMTPAEHKQLEKLS
176 LPHNMFWVPWVWFANLSMKAWLGGRIRDPILLQSL
211 LNEMNTLRTQCGHLYAYDWISIPLVYTQVVTVAVY
246 SFFLTCLVGRQFLNPAKAYPGHELDLVVPVFTFLQ
281 FFFYVGWLKVAEQLINPFGEDDDDFETNWIVDRNL
316 QVSLLAVDEMHQDLPRMEPDMYWNKPEPQPPYTAA
351 SAQFRRASFMGSTFNISLNKEEMEFQPNQEDEEDA
386 HAGIIGRFLGLQSHDHHPPRANSRTKLLWPKRESL
421 LHEGLPKNHKAAKQNVRGQEDNKAWKLKAVDAFKS
456 APLYQRPGYYSAPQTPLSPTPMFFPLEPSAPSKLH
491 SVTGIDTKDKSLKTVSSGAKKSFELLSESDGALME
526 HPEVSQVRRKTVEFNLTDMPEIPENHLKEPLEQSP
561 TNIHTTLKDHMDPYWALENRDEAHS

TM4 domain (aa 275-288)

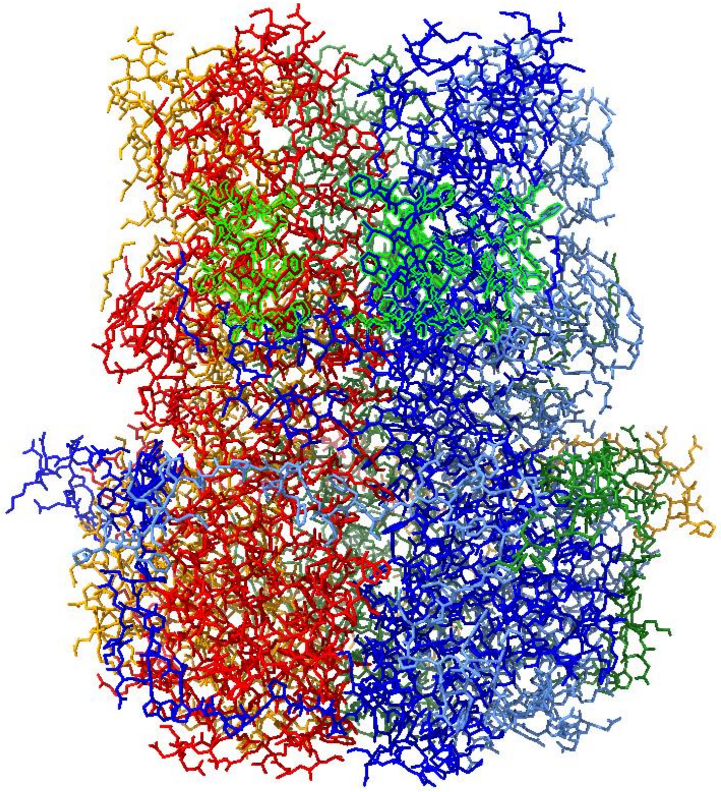

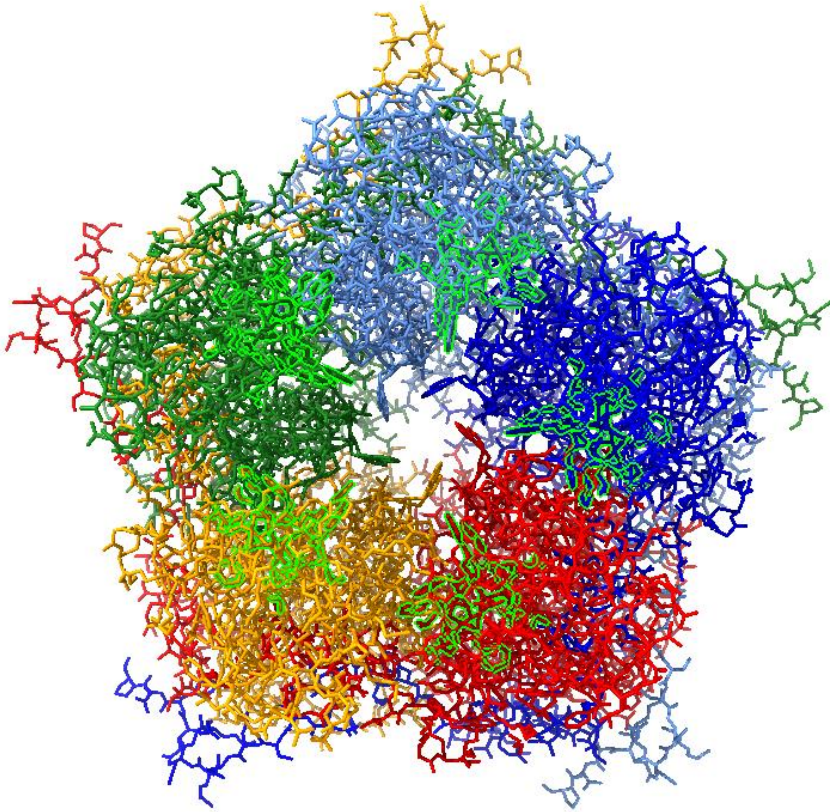

```
1   MTITYTSQVANARLGSFSRLLLCWRGSIYKLLYGE
36  FLIFLLCYYIIRFIYRLALTEEQQLMFEKLTLYCD
71  SYIQLIPISFVLGFYVTLVVTRWWNQYENLPWPDR
106 LMSLVSGFVEGKDEQGRLLRRTLIRYANLGNVLIL
141 RSVSTAVYKRFPSAQHLVQAGFMTPAEHKQLEKLS
176 LPHNMFWVPWVWFANLSMKAWLGGRIRDPILLQSL
211 LNEMNTLRTQCGHLYAYDWISIPLVYTQVVTVAVY
246 SFFLTCLVGRQFLNPAKAYPGHELDLVVPVFTFLQ
281 FFFYVGWLKVAEQLINPFGEDDDDFETNWIVDRNL
316 QVSLLAVDEMHQDLPRMEPDMYWNKPEPQPPYTAA
351 SAQFRRASFMGSTFNISLNKEEMEFQPNQEDEEDA
386 HAGIIGRFLGLQSHDHHPPRANSRTKLLWPKRESL
421 LHEGLPKNHKAAKQNVRGQEDNKAWKLKAVDAFKS
456 APLYQRPGYYSAPQTPLSPTPMFFPLEPSAPSKLH
491 SVTGIDTKDKSLKTVSSGAKKSFELLSESDGALME
526 HPEVSQVRRKTVEFNLTDMPEIPENHLKEPLEQSP
561 TNIHTTLKDHMDPYWALENRDEAHS
```

Cytosolic C termini domain (aa 289-377)

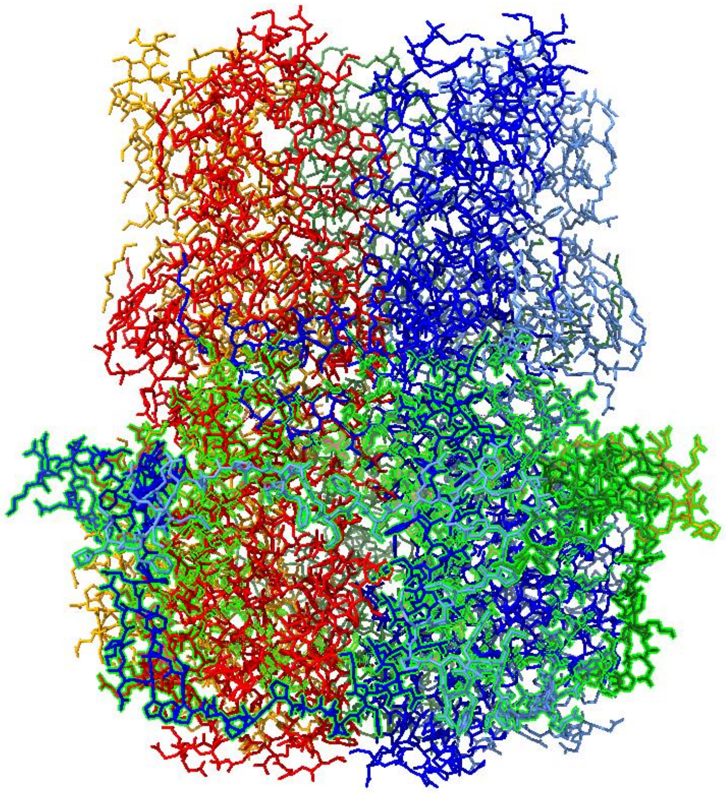

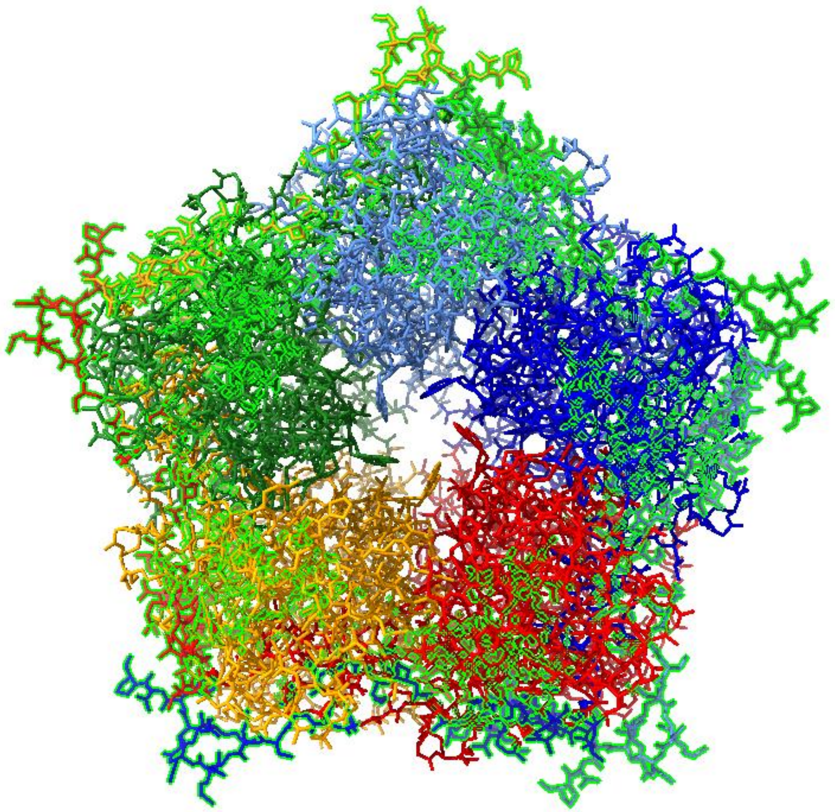

```
  1 MTITYTSQVANARLGSFSRLLLCWRGSIYKLLYGE
 36 FLIFLLCYYIIRFIYRLALTEEQQLMFEKLTLYCD
 71 SYIQLIPISFVLGFYVTLVVTRWWNQYENLPWPDR
106 LMSLVSGFVEGKDEQGRLLRRTLIRYANLGNVLIL
141 RSVSTAVYKRFPSAQHLVQAGFMTPAEHKQLEKLS
176 LPHNMFWVPWVWFANLSMKAWLGGRIRDPILLQSL
211 LNEMNTLRTQCGHLYAYDWISIPLVYTQVVTVAVY
246 SFFLTCLVGRQFLNPAKAYPGHELDLVVPVFTFLQ
281 FFFYVGWLKVAEQLINPFGEDDDDFETNWIVDRNL
316 QVSLLAVDEMHQDLPRMEPDMYWNKPEPQPPYTAA
351 SAQFRRASFMGSTFNISLNKEEMEFQPNQEDEEDA
386 HAGIIGRFLGLQSHDHHPPRANSRTKLLWPKRESL
421 LHEGLPKNHKAAKQNVRGQEDNKAWKLKAVDAFKS
456 APLYQRPGYYSAPQTPLSPTPMFFPLEPSAPSKLH
491 SVTGIDTKDKSLKTVSSGAKKSFELLSESDGALME
526 HPEVSQVRRKTVEFNLTDMPEIPENHLKEPLEQSP
561 TNIHTTLKDHMDPYWALENRDEAHS
```

Supplementary Figure S2: 3D location of BEST1 domains. All nine BEST1 domains are highlighted based on UCSF ChimeraX software.
