# Supplementary material for: Novel BEST1 Variant Characterization in a Large French Cohort in Light of Updated Bestrophin-1 Structure–Function Correlation
Source: Invest Ophthalmol Vis Sci. 2025 Sep 2;66(12):4. doi: 10.1167/iovs.66.12.4 (PMC12410269; doi:10.1167/iovs.66.12.4)
Supplement: Supplement 3 [file iovs-66-12-4_s003.pdf]

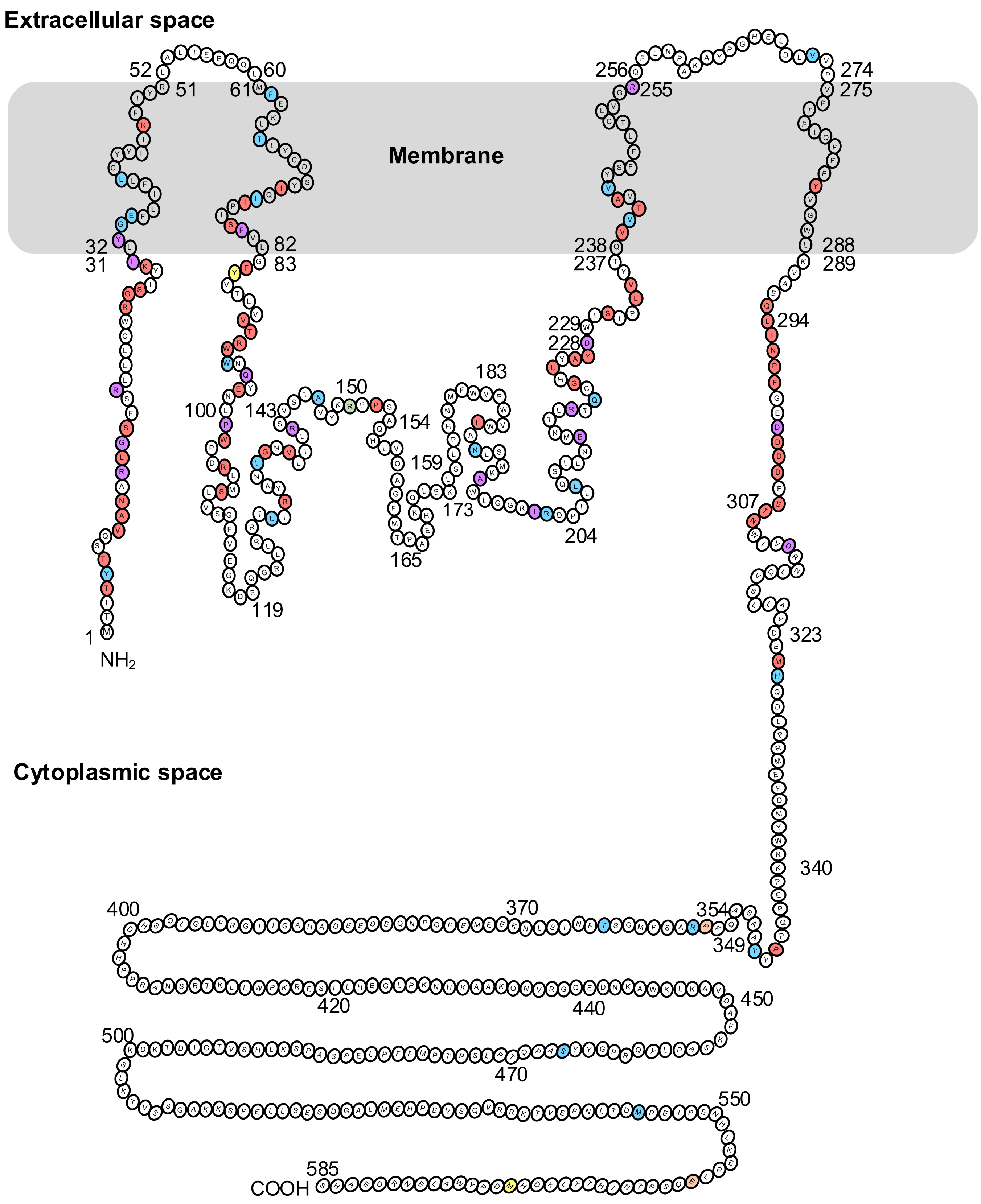

- Adult-onset vitelliform macular dystrophy only
- Autosomal Dominant Vitreoretinochoroidopathy only
- Autosomal recessive bestrophinopathy only
- Best vitelliform macular dystrophy only
- Retinitis pigmentosa only
- Multiple phenotypes

Supplementary Figure S3: French *BEST1* variants location. This drawing represents the protein organization of BEST1 channel in the plasma membrane and position of the amino acid residues affected by the french *BEST1* variants. Colors indicate the associated phenotypes. Domains organization and helices length have been drawed based on Uniprot and Alphafold predictions.
