# Supplementary material for: Novel BEST1 Variant Characterization in a Large French Cohort in Light of Updated Bestrophin-1 Structure–Function Correlation
Source: Invest Ophthalmol Vis Sci. 2025 Sep 2;66(12):4. doi: 10.1167/iovs.66.12.4 (PMC12410269; doi:10.1167/iovs.66.12.4)
Supplement: Supplement 4 [file iovs-66-12-4_s004.pdf]

## French VUS variants :

- c.324C>A p.(Ser108Arg)
- c.403G>A p.(Gly135Ser)
- c.938G>A p.(Arg313Lys)
- c.1063C>T p.(Arg355Cys)
- c.1087A>C p.(Thr363Pro)
- c.1397G>C p.(Ser466Thr)
- c.1632G>A p.(Met544Ile)
- c.1669G>A p.(Glu557Lys)

**Predicted Stability Change ($\triangle\triangle G^{Stability}$)**
**-0.46 kcal/mol**
**(Destabilising)**

# p.(Ser108Arg)

*Wild-type*

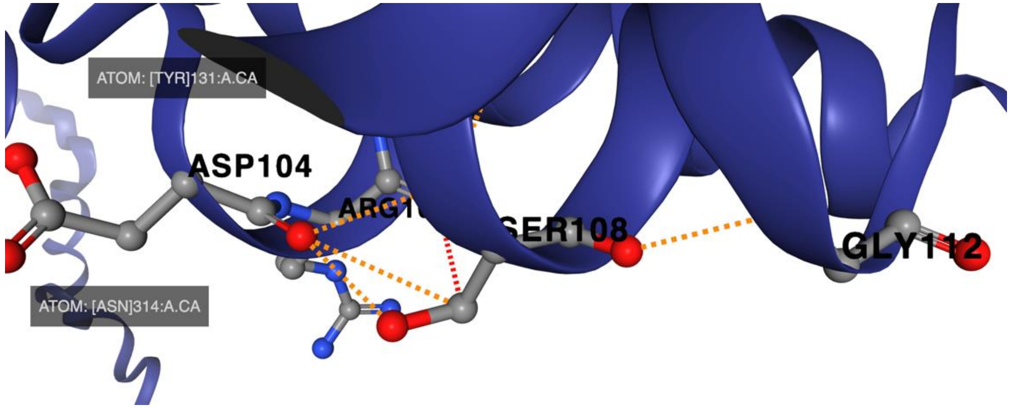


*Mutant*

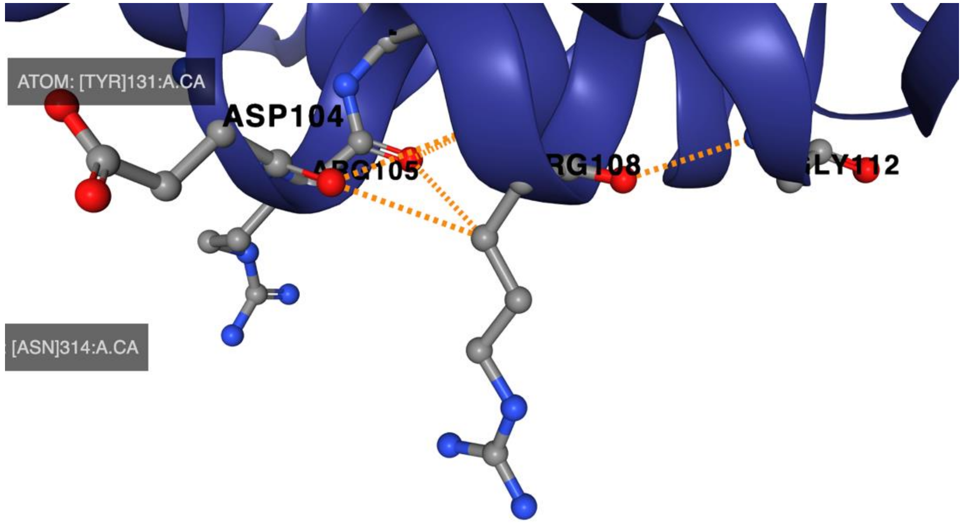


Hydrogen Bond    Polar    Carbonyl
Hydrophobic    Van der Waals

## p.(Gly135Ser)

**Predicted Stability Change ($\Delta\Delta G^{Stability}$)**
**-1.35 kcal/mol**
**(Destabilising)**

*Wild-type*

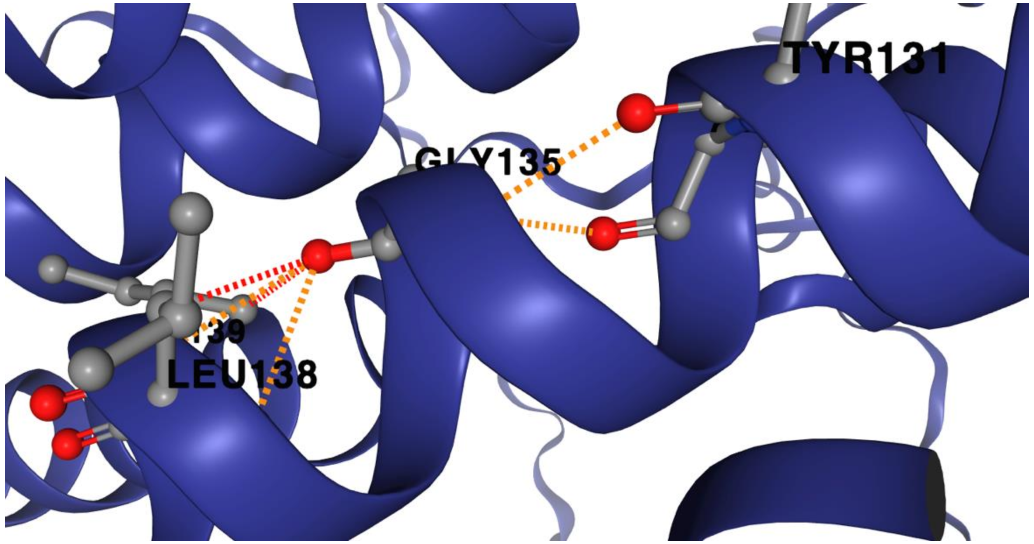


*Mutant*

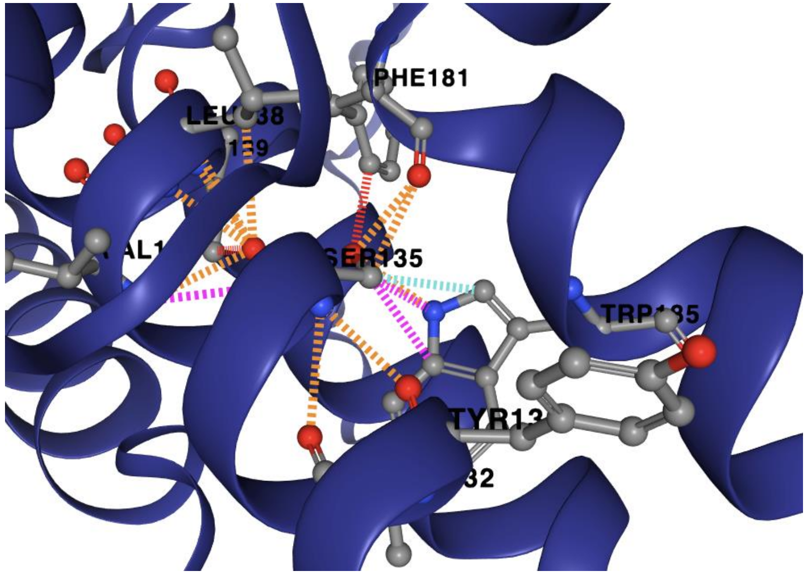


Hydrogen Bond
Polar
Carbonyl
Hydrophobic
Van der Waals

## p.(Arg313Lys)

**Predicted Stability Change (△△G^Stability^)**

**-1.61 kcal/mol**
**(Destabilising)**

*Wild-type*

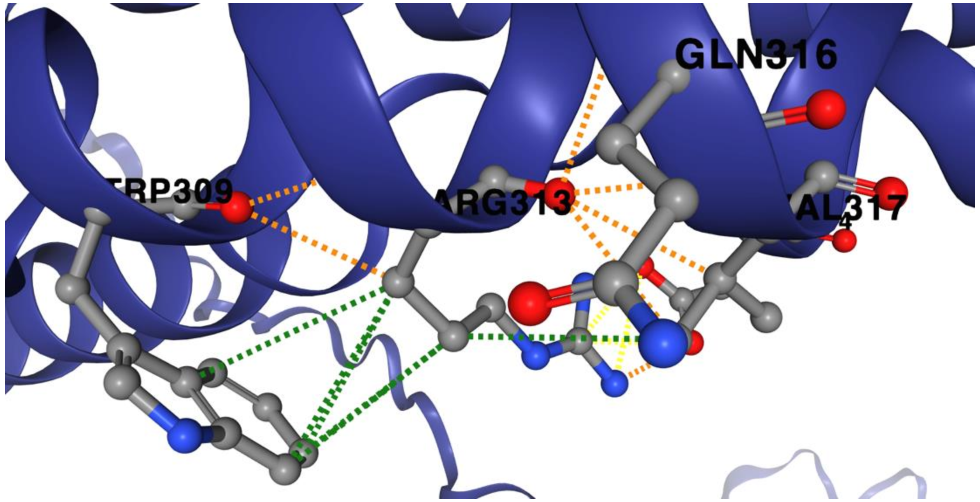


*Mutant*

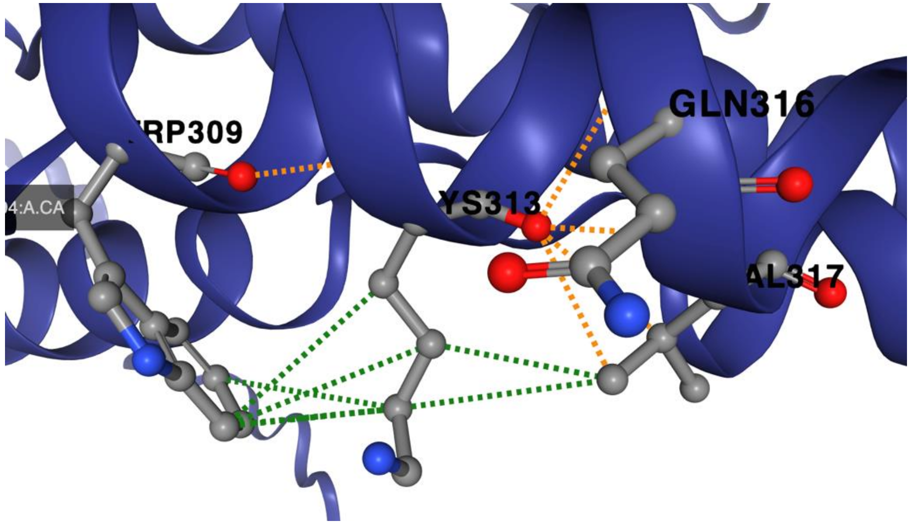


Hydrogen Bond
Hydrophobic
Polar
Van der Waals
Carbonyl

**Predicted Stability Change ($\Delta\Delta G^{Stability}$)**

**0.38 kcal/mol**
**(Stabilising)**

# p.(Arg355Cys)

*Wild-type*

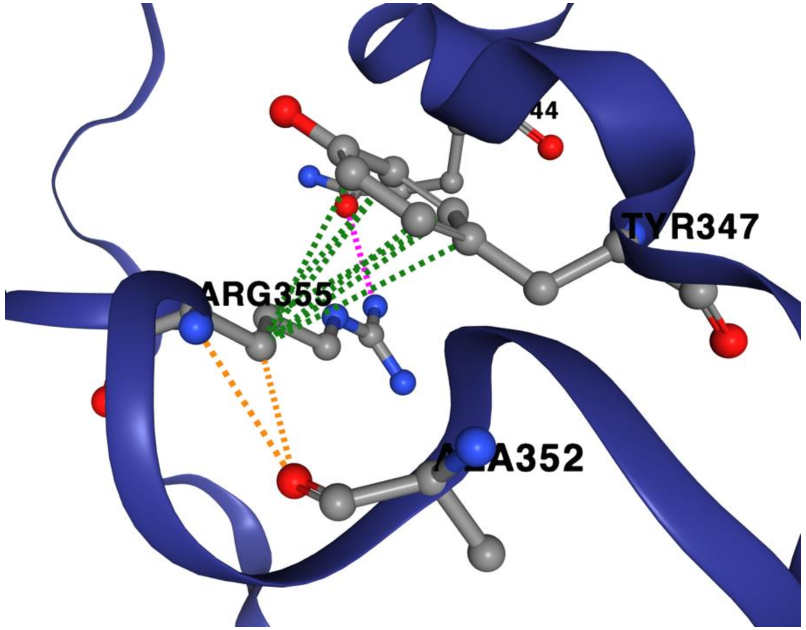


*Mutant*

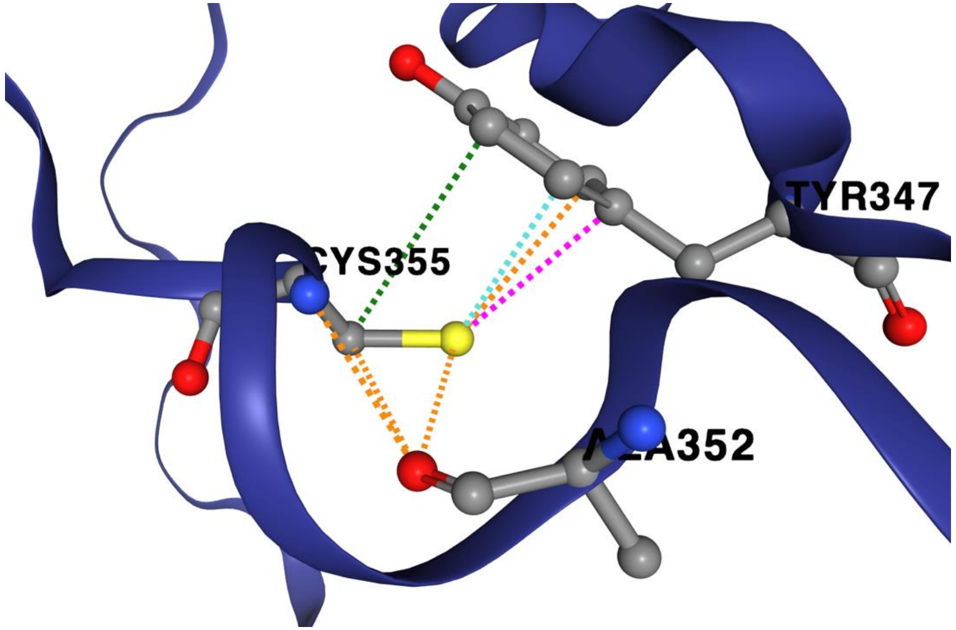


Hydrogen Bond
Hydrophobic
Polar
Van der Waals
Carbonyl

**Predicted Stability Change ($\triangle\triangle G^{Stability}$)**

**0.14 kcal/mol**

**(Stabilising)**

# p.(Thr363Pro)

*Wild-type* *Mutant*

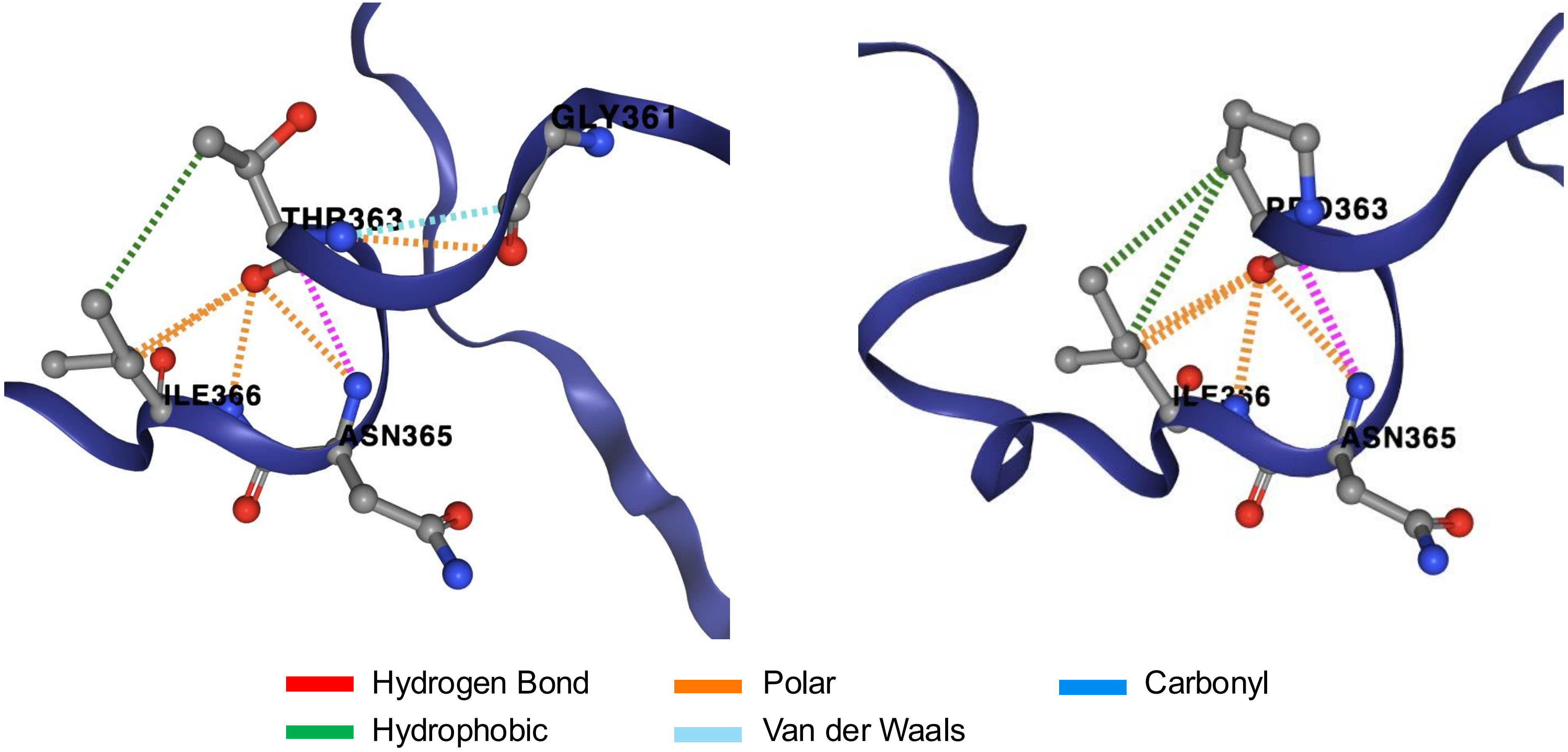

# p.(Ser466Thr)

**Predicted Stability Change ($\triangle\triangle G^{Stability}$)**
**-0.07 kcal/mol**
**(Destabilising)**

*Wild-type*

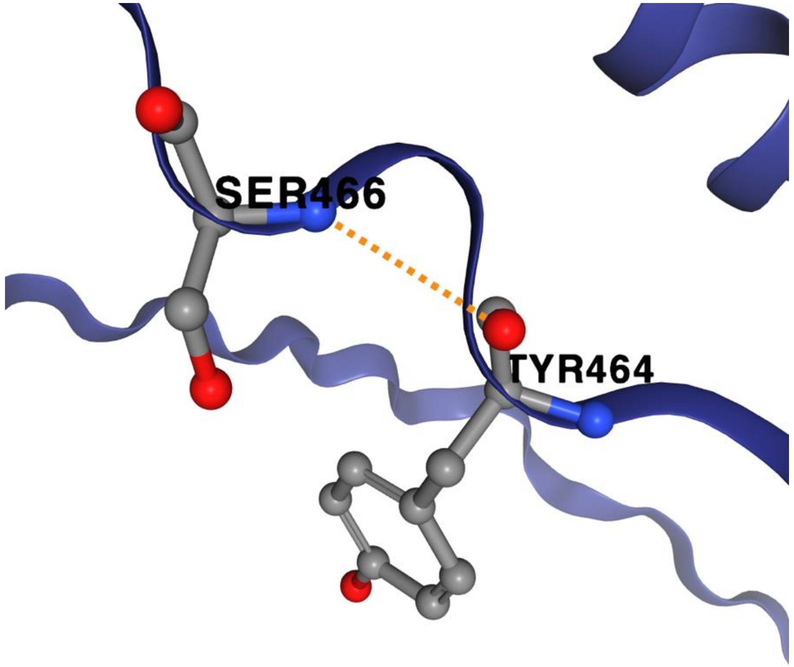


*Mutant*

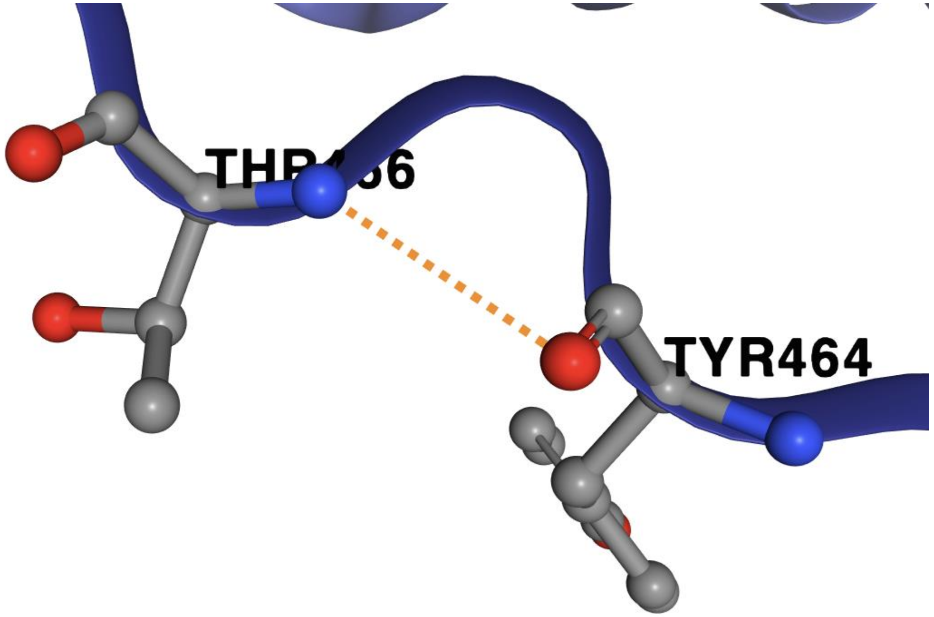


Hydrogen Bond
Hydrophobic
Polar
Van der Waals
Carbonyl

## p.(Met544Ile)

**Predicted Stability Change ($\triangle\triangle G^{Stability}$)**

**-0.1 kcal/mol**
**(Destabilising)**

*Wild-type*

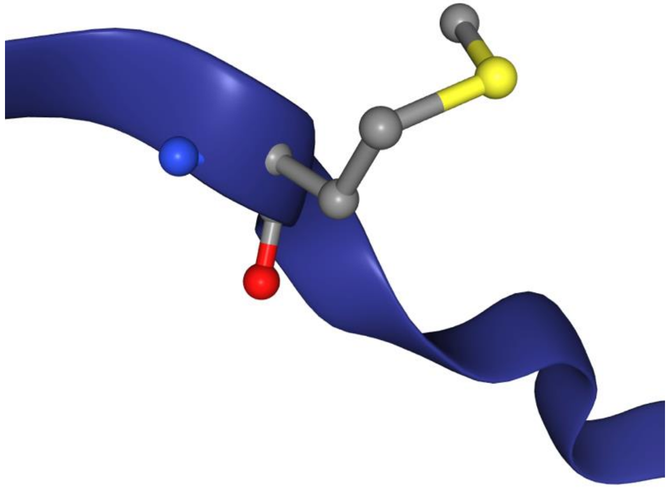

*Mutant*

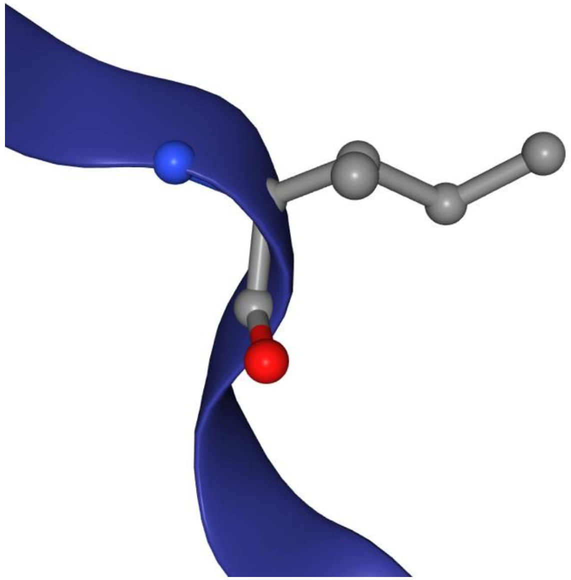

Hydrogen Bond
Hydrophobic
Polar
Van der Waals
Carbonyl

**Predicted Stability Change ($\triangle\triangle G^{Stability}$)**
**0.2 kcal/mol**
**(Stabilising)**

# p.(Glu557Lys)

*Wild-type*

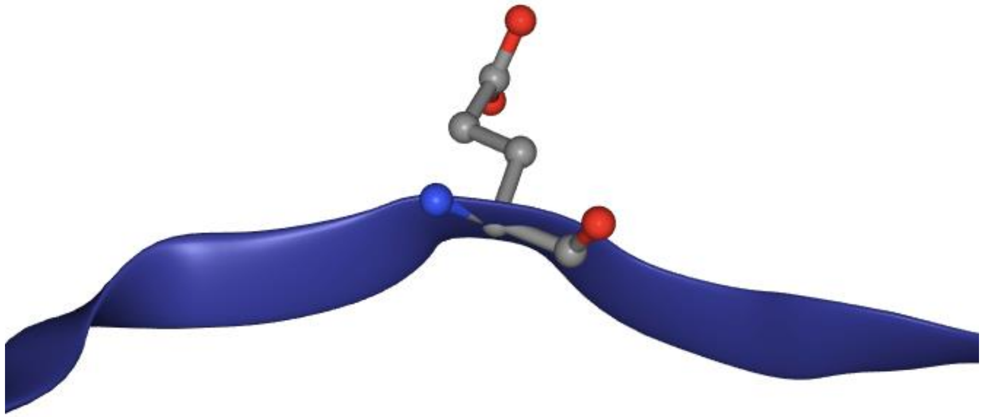

*Mutant*

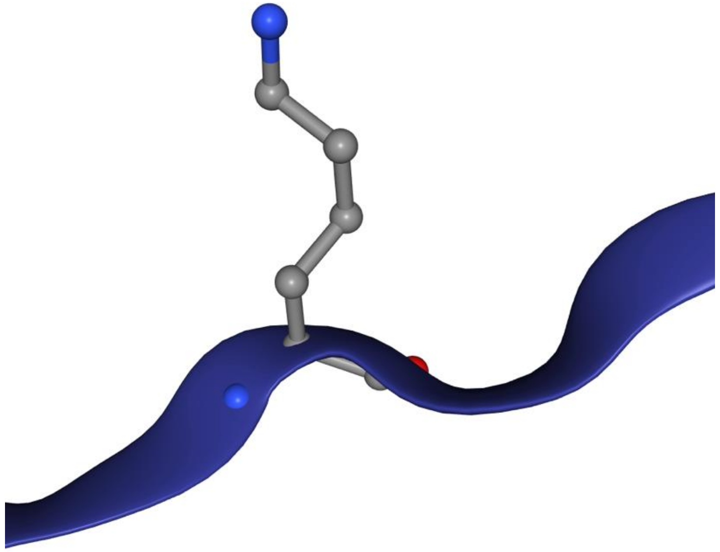

Hydrogen Bond
Hydrophobic
Polar
Van der Waals
Carbonyl

**Supplementary Figure S4: 3D Structure of eight French variants of unknown significance (VUS).** Amino acid interactions in wild-type and mutant versions are depicted (based on DynaMut2 software). Predicted stability changes are indicated in ΔΔG (kcal/mol). ΔG represents the effect of the variant on protein stability in terms of Gibbs free energy of unfolding. ΔΔG is the difference in unfolding free energy between two proteins, the wild-type and the mutant.
