# Supplementary material for: Novel BEST1 Variant Characterization in a Large French Cohort in Light of Updated Bestrophin-1 Structure–Function Correlation
Source: Invest Ophthalmol Vis Sci. 2025 Sep 2;66(12):4. doi: 10.1167/iovs.66.12.4 (PMC12410269; doi:10.1167/iovs.66.12.4)
Supplement: Supplement 5 [file iovs-66-12-4_s005.pdf]

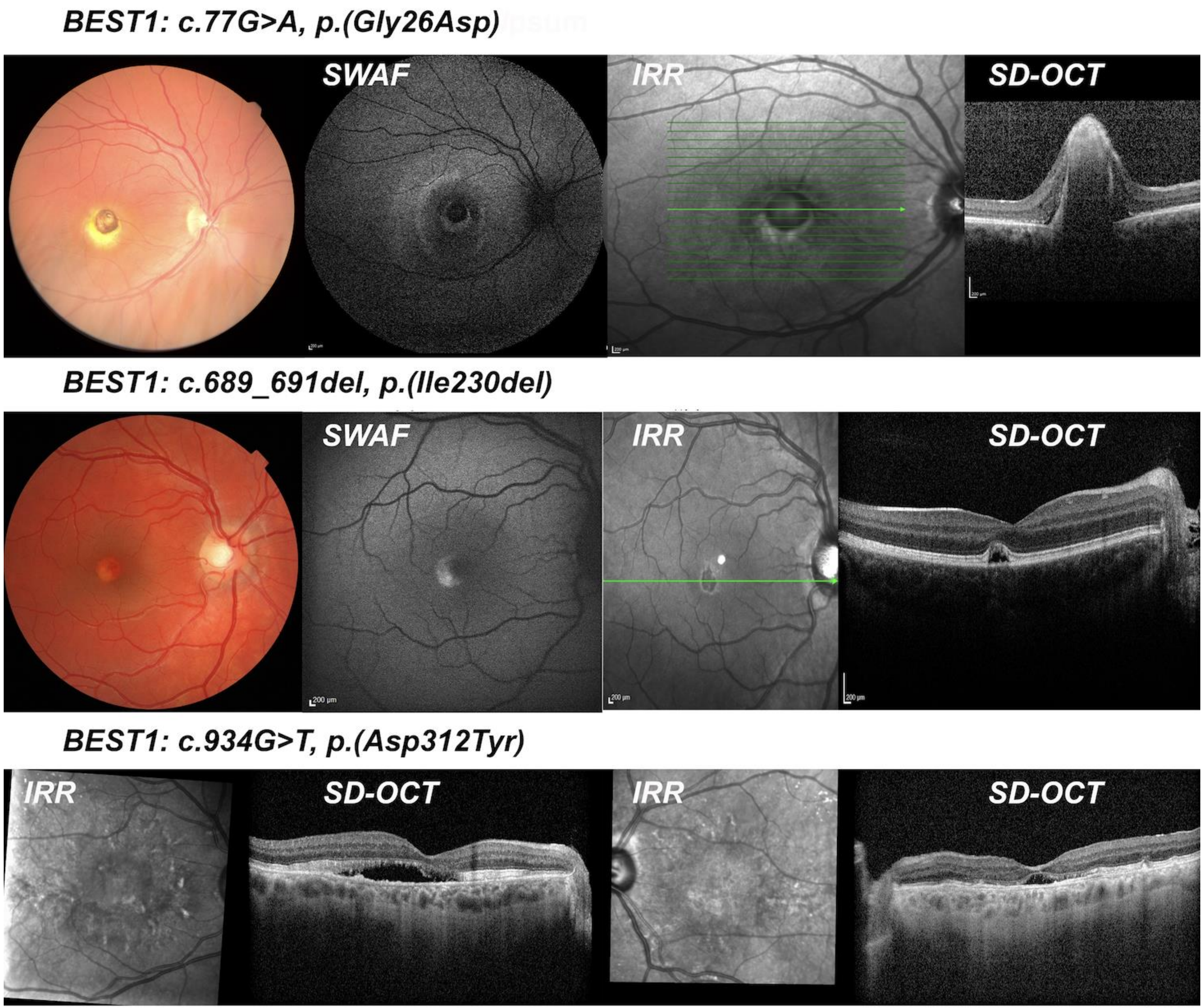
BEST1: c.77G>A, p.(Gly26Asp)
SWAF
IRR
SD-OCT
BEST1: c.689_691del, p.(Ile230del)
SWAF
IRR
SD-OCT
BEST1: c.934G>T, p.(Asp312Tyr)
IRR
SD-OCT
IRR
SD-OCT

Supplementary Figure S5: Clinical examination of patients carrying new French *BEST1* variants

Fundus, short wavelength autofluorescence (SWAF), infrared reflective (IRR) fundus images and spectral domain optical coherence tomography (HD-OCT) are represented.
