# Supplementary material for: Novel BEST1 Variant Characterization in a Large French Cohort in Light of Updated Bestrophin-1 Structure–Function Correlation
Source: Invest Ophthalmol Vis Sci. 2025 Sep 2;66(12):4. doi: 10.1167/iovs.66.12.4 (PMC12410269; doi:10.1167/iovs.66.12.4)
Supplement: Supplement 6 [file iovs-66-12-4_s006.pdf]

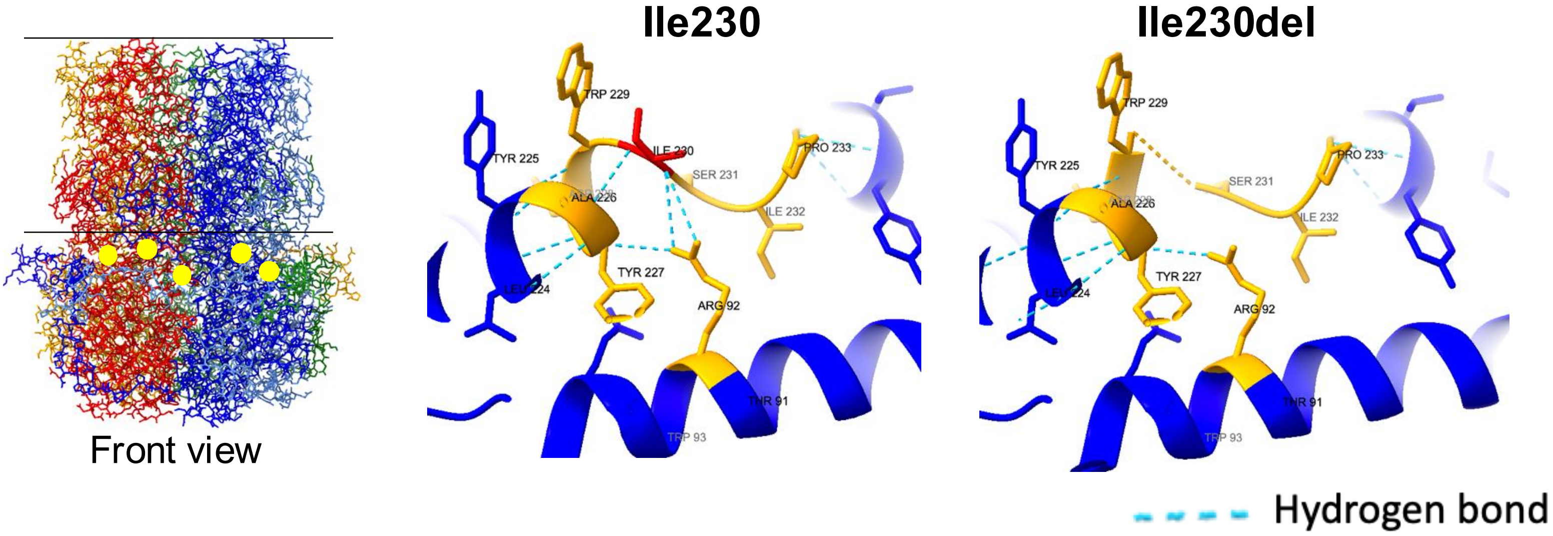

Supplementary Figure S6: Novel Ile230del French variant and 3D Structure. On the left, location of the variant in the protein with front view. On the right, amino acid interactions in normal and mutant versions (based on UCSF ChimeraX software). Yellow dots represent the amino acid location in the 3D structure.
