# Supplementary material for: Novel BEST1 Variant Characterization in a Large French Cohort in Light of Updated Bestrophin-1 Structure–Function Correlation
Source: Invest Ophthalmol Vis Sci. 2025 Sep 2;66(12):4. doi: 10.1167/iovs.66.12.4 (PMC12410269; doi:10.1167/iovs.66.12.4)
Supplement: Supplement 7 [file iovs-66-12-4_s007.pdf]

A. Best disease. *BEST1*: c.218T>A, p.(Ile73Asn)

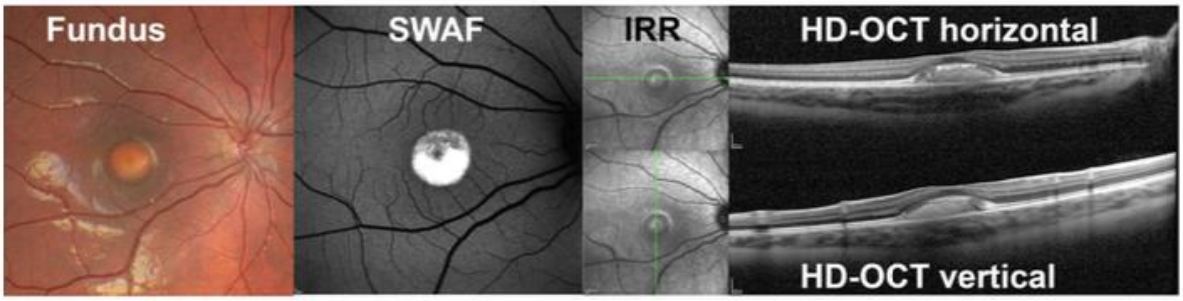

B. Adult vitelliform macular dystrophy. *BEST1*: c.934G>A, p.(Asp312Asn)

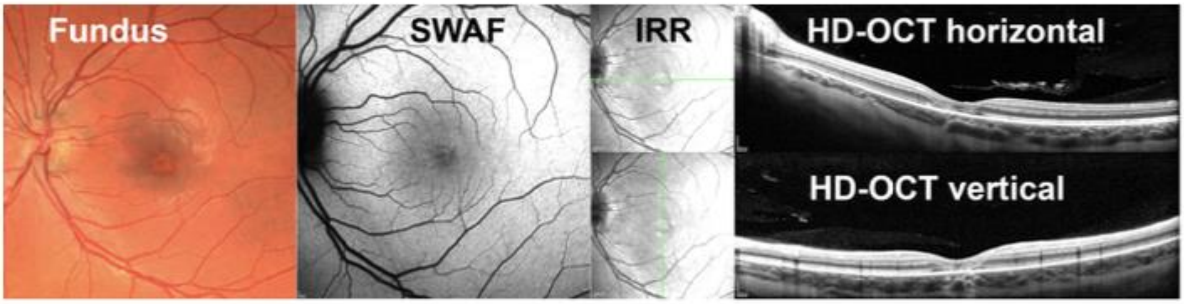

C. Autosomal recessive bestrophinopathy.
BEST1: c.570C>G, p.(Asn190Lys)/c.621T>C, p.(Ile201Thr)

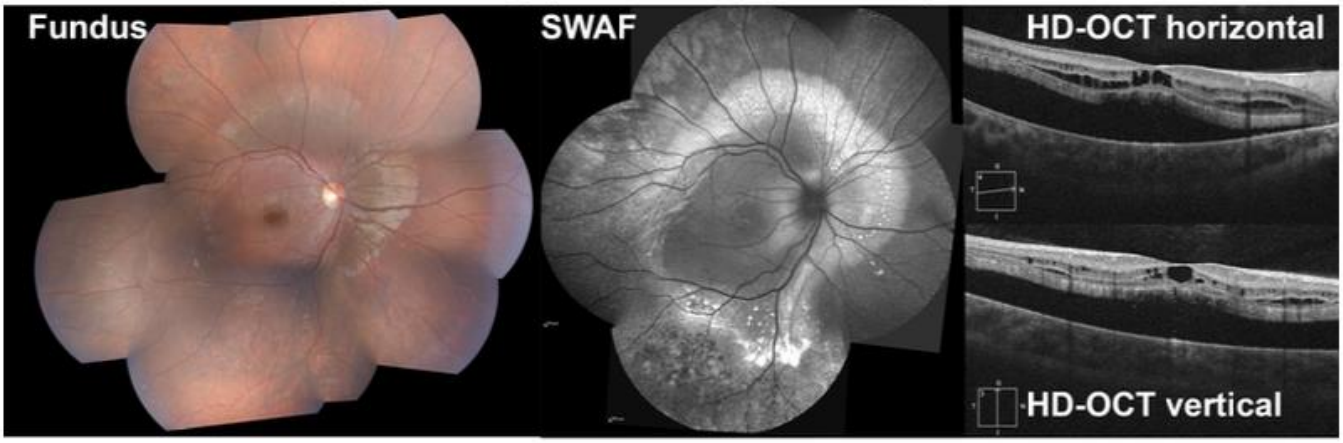

D. ADVIRC. *BEST1*: c.256G>A, p.(Val86Met)

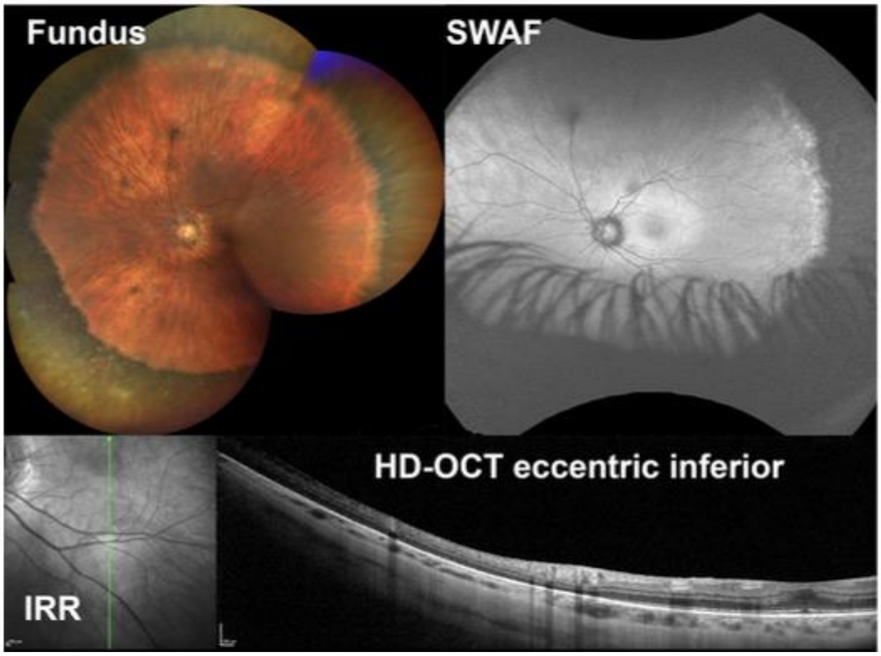

Supplementary Figure S7:Genotype-phenotype correlation.

A. Best disease. Egg-yolk macular lesion which appears intensely hyperautofluorescent on SWAF. Dense subretinal deposit on HD-OCT.
B. Adult vitelliform macular dystrophy. Small fovea-centered atrophic lesion
C. Autosomal recessive betrophinopathy. Large serous retinal detachment in the posterior pole. On SWAF, the borders of this detachement are hyperautofluorescent and there are additional hyperautofluoresent spots surrounding the central lesion. On HD-OCT, flat retinal detachment in posterior pole and intraretinal microkysts.
D. Autosomal-dominant vitreo-retino-choroidopathy. 360° demarcation zone in the peripheral retina. On SWAF, retina beyond demarcation zone is hypoautofluorescent. On HD-OCT, loss of outer retinal layers in peripheral retina.
