# Supplementary material for: Novel BEST1 Variant Characterization in a Large French Cohort in Light of Updated Bestrophin-1 Structure–Function Correlation
Source: Invest Ophthalmol Vis Sci. 2025 Sep 2;66(12):4. doi: 10.1167/iovs.66.12.4 (PMC12410269; doi:10.1167/iovs.66.12.4)
Supplement: Supplement 8 [file iovs-66-12-4_s008.docx]

**Supplementary Table S1: Structural organization of BEST1 channel**

Extracellular, cytosolic and transmembrane domains are presented, with residues boundaries, and location of the different conformations (helix, turn, beta stand). Functional regions with key roles amino acids are shown.

Sources Uniprot and Alphafold.

| **Domains** | **Amino acids** | **Helix conformations** | **Beta strand conformations** | **Turn conformations** | **Amino acids involved** | **Functional domain** |
| --- | --- | --- | --- | --- | --- | --- |
| Cytosolic N-termini | 1-31 | 6-9  16-19  20-22 |  |  | Ala10 | Ca^2+^ clasp site |
| **TM1** | 32-51 | 28-52 |  |  |  |  |
| Extracellular loop TM1-TM2 | 52-60 | 56-71 |  |  |  | Outer entryway |
| **TM2** | 61-82 | 76-98 |  | 72-75 | Ile76  Phe80 | Neck |
| Cytosolic loop TM2-TM3 | 83-237 | 104-113  119-143  145-150  154-159  165-174  183-197  204-229 | 200-203 |  | Phe84  Ile205 | Cytosolic aperture |
| **TM3** | 238-255 | 234-254 |  |  |  |  |
| Extracellular loop TM3-TM4 | 256-274 | 260-262 |  |  |  |  |
| **TM4** | 275-288 | 275-294 |  |  |  |  |
| Cytosolic C-termini | 289-585 | 307-323  349-354 | 299-301  374-376 | 324-327  336-339  362-365  370-373 | Asp293 Pro297  Glu300 Asp301 Asp302 Asp303 Asp304  Glu300-Glu306 | Ca^2+^ clasp site  Conserved carboxylate-rich loop |
