# Supplementary material for: Novel BEST1 Variant Characterization in a Large French Cohort in Light of Updated Bestrophin-1 Structure–Function Correlation
Source: Invest Ophthalmol Vis Sci. 2025 Sep 2;66(12):4. doi: 10.1167/iovs.66.12.4 (PMC12410269; doi:10.1167/iovs.66.12.4)
Supplement: Supplement 10 [file iovs-66-12-4_s010.docx]

**Supplementary Table S3: Distribution of variants type**

**A) LOVD cohort**

| **Variant type** | **Number of unique variants (%)** | **Number of patients (%)** |
| --- | --- | --- |
| Deletion (in frame) | 11 (2.3) | 81 (3.7) |
| Deletion - Insertion (in frame) | 4 (0.8) | 6 (0.3) |
| Frameshift (deletion/insertion/duplication) or splice | 48 (9.8) | 100 (4.5) |
| Insertion (in frame) | 1 (0.2) | 2 (0.1) |
| Intronic deletion - insertion | 1 (0.2) | 1 (0.05) |
| Intronic duplication | 2 (0.4) | 3 (0.1) |
| Intronic insertion | 1 (0.2) | 1 (0.05) |
| Intronic substitution | 20 (4.1) | 23 (1.0) |
| Large deletion | 2 (0.4) | 3 (0.1) |
| Missense | 353 (72.3) | 1876 (85.0) |
| Nonsense | 20 (4.1) | 52 (2.4) |
| Synonymous | 20 (4.1) | 54 (2.4) |
| Variation on UTR = 5'- or 3'-UTR | 5 (1.0) | 6 (0.3) |
| **Total** | **488** | **2208** |

**B) French cohort.** ^1^, one deletion in *trans* with a nonsense for one patient; ^2^, two deletions in *trans* with a missense for three patients; ^3^, two frameshift deletions in *trans* with a missense for two patients; ^4^, one frameshift deletion in *cis* with a missense; ^5^, in *trans* with a missense for each patient; ^6^, two nonsenses in *trans* with a missense; ^7^, one *in trans* with a splice variant, two *in trans* with a missense

| **Variant type** | **Number of unique variants (%)** | | | **Number of patients (%)** |
| --- | --- | --- | --- | --- |
| Deletion ^(1) (2)^ (in frame) | | 5 (3.3) | 25 (5.6) | |
| Deletion-Insertion (in frame) | | 2 (1.3) | 4 (0.9) | |
| Frameshift (deletion ^(3) (4)^ or duplication ^(5)^) or splice ^(6)^ | | 7 (4.7) | 13 (2.9) | |
| Large deletion ^(5)^ | | 1 (0.7) | 2 (0.4) | |
| Missense | | 132 (88.0) | 401 (89.1) | |
| Nonsense ^(7)^ | | 3 (2.0) | 5 (1.1) | |
| **Total** | | **150** | **450** | |
