# Supplementary material for: Novel BEST1 Variant Characterization in a Large French Cohort in Light of Updated Bestrophin-1 Structure–Function Correlation
Source: Invest Ophthalmol Vis Sci. 2025 Sep 2;66(12):4. doi: 10.1167/iovs.66.12.4 (PMC12410269; doi:10.1167/iovs.66.12.4)
Supplement: Supplement 11 [file iovs-66-12-4_s011.docx]

**Supplementary Table S4: Distribution of variants by protein domain**

**A) LOVD cohort**

| **Protein domain** | **Number of variants (%)** | **Number of patients (%)** |
| --- | --- | --- |
| N-terminal | 61 (12.5) | 320 (14.5) |
| Cytosolic N-termini + TM1 | 2 (0.4) | 3 (0.1) |
| TM1 | 17 (3.5) | 66 (3.0) |
| TM1-TM2 extracellular loop | 7 (1.4) | 8 (0.4) |
| TM2 | 31 (6.4) | 80 (3.6) |
| TM2-TM3 cytosolic loop | 176 (36.1) | 924 (41.8) |
| TM3 | 21 (4.3) | 160 (7.2) |
| TM3-TM4 extracellular loop | 8 (1.6) | 19 (0.9) |
| TM4 | 12 (2.5) | 43 (1.9) |
| C-terminal | 153 (31.4) | 585 (26.5) |
| **Total** | **488** | **2208** |

**B) French cohort.** *Nota bene: 33 patients are comprised in two protein domain lines because there are heterozygous compounds with two variations in trans.*

| **Protein domain** | | **Number of variants (%)** | **Number of patients (%)** |
| --- | --- | --- | --- |
| N-terminal | 25 (16.7) | | 79 (17.5) |
| Cytosolic N-termini + TM1 | 1 (0.7) | | 2 (0.4) |
| TM1 | 7 (4.7) | | 12 (2.7) |
| TM2 | 11 (7.3) | | 27 (6.0) |
| TM2-TM3 cytosolic loop | 57 (38.0) | | 210 (46.7) |
| TM3 | 6 (4.0) | | 12 (2.7) |
| TM3-TM4 extracellular loop | 1 (0.7) | | 1 (0.2) |
| TM4 | 1 (0.7) | | 4 (0.9) |
| C-terminal | 41 (27.3) | | 103 (22.9) |
| **Total** | **150** | | **450** |

**C) Schematic representation of the distribution in both cohorts** (LOVD on the left and French on the right)
