# Supplementary material for: Novel BEST1 Variant Characterization in a Large French Cohort in Light of Updated Bestrophin-1 Structure–Function Correlation
Source: Invest Ophthalmol Vis Sci. 2025 Sep 2;66(12):4. doi: 10.1167/iovs.66.12.4 (PMC12410269; doi:10.1167/iovs.66.12.4)
Supplement: Supplement 12 [file iovs-66-12-4_s012.docx]

**Supplementary Table S5: Distribution of variant types by main domain and by phenotype**

**A) LOVD cohort.** *p*-value was calculated using a Chi-square test (missense or synonymous substitution vs truncated). Total number of variants: n=488

|  | **TM2-TM3 loop (%)** | **C-Ter (%)** | **N-Ter (%)** | **TM3 (%)** | ***p-value*** |
| --- | --- | --- | --- | --- | --- |
| **Missense or synonymous substitution** | **143 (81.3)** | **106 (69.3)** | **54 (88.5)** | **12 (57.1)** | 0.0011 |
| **Deletion** | 5 (2.8) | 4 (2.6) | 0 (0,0) | 0 (0.0) |  |
| **Deletion - insertion** | 3 (1.7) | 1 (0.7) | 0 (0,0) | 0 (0.0) |  |
| **Frameshift (deletion or duplication or insertion)** | 8 (4.5) | 12 (7.8) | 1 (1.6) | 3 (14.3) |  |
| **Insertion** | 1 (0.6) | 0 (0.0) | 0 (0.0) | 0 (0.0) |  |
| **Intronic or UTR variation** | 4 (2.3) | 17 (11.1) | 0 (0.0) | 5 (23.8) |  |
| **Splice** | 4 (2.3) | 7 (4.6) | 3 (4.9) | 1 (4.8) |  |
| **Stop** | 8 (4.5) | 6 (3.9) | 3 (4.9) | 0 (0.0) |  |
| **Total truncated** | **33 (18.7)** | **47 (30.7)** | **7 (11.5)** | **9 (42.3)** |  |
| **Total** | **176 (36.1)** | **153 (31.3)** | **61 (12.5)** | **21 (4.3)** |  |

|  | **TM2-TM3 loop (%)** | **C-Ter (%)** | **N-Ter (%)** | **TM3 (%)** |
| --- | --- | --- | --- | --- |
| **Phenotypes** |  |  |  |  |
| **ADVIRC** | 4 (2.5) | 1 (0.8) | 0 (0.0) | 1 (6.3) |
| **AMD** | 1 (0.6) | 3 (2.4) | 1 (1.7) | 0 (0.0) |
| **ARB** | 69 (42.3) | 37 (30.1) | 13 (22.0) | 9 (56.3) |
| **AVMD** | 2 (1.2) | 2 (1.6) | 0 (0.0) | 0 (0.0) |
| **Butterfly-shaped pattern dystrophy** | 0 (0.0) | 1 (0.8) | 0 (0.0) | 0 (0.0) |
| **BVMD** | 68 (41.7) | 62 (50.4) | 43 (72.9) | 5 (31.3) |
| **Multifocal vitelliform dystrophy** | 1 (0.6) | 0 (0.0) | 0 (0.0) | 0 (0.0) |
| **Multiple phenotypes** | 14 (8.6) | 12 (9.8) | 2 (3.4) | 1 (6.2) |
| **RP** | 4 (2.5) | 5 (4.1) | 0 (0.0) | 0 (0.0) |
| **Total** | **163 (33.4)** | **123 (25.2)** | **59 (12.1)** | **16 (3.3)** |

*Without counting unknown phenotypes*

**B) French cohort.** *p*-value was calculated using a Fisher’s exact test (missense vs truncated). Total number of variants: n=150

|  | **TM2-TM3 loop (%)** | **C-Ter (%)** | **N-Ter (%)** | **TM3 (%)** | ***p-value*** |
| --- | --- | --- | --- | --- | --- |
| **Missense** | **50 (87.7)** | **34 (82.9)** | **24 (96.0)** | **6 (100.0)** | 0.4286 |
| **Deletion** | 3 (5.3) | 1 (2.4) | 0 (0,0) | 0 (0.0) |  |
| **Deletion - insertion** | 1 (1.8) | 1 (2.4) | 0 (0,0) | 0 (0.0) |  |
| **Frameshift (deletion or duplication or insertion)** | 0 (0.0) | 4 (9.8) | 0 (0,0) | 0 (0.0) |  |
| **Splice** | 2 (3.6) | 1 (2.4) | 0 (0,0) | 0 (0.0) |  |
| **Stop** | 1 (1.8) | 0 (0.0) | 1 (4.0) | 0 (0.0) |  |
| **Total truncated** | **7 (12.3)** | **7 (17.1)** | **1 (4.0)** | **0 (0.0)** |  |
| **Total** | **57 (38.0)** | **41 (27.3)** | **25 (16.7)** | **6 (4.0)** |  |

|  | **TM2-TM3 loop (%)** | **C-Ter (%)** | **N-Ter (%)** | **TM3 (%)** |
| --- | --- | --- | --- | --- |
| **Phenotypes** |  |  |  |  |
| **ADVIRC** | 1 (1.7) | 1 (2.4) | 0 (0.0) | 0 (0.0) |
| **ARB** | 16 (28.1) | 10 (24.4) | 3 (12.0) | 2 (33.3) |
| **AVMD** | 1 (1.7) | 1 (2.4) | 0 (0,0) | 0 (0,0) |
| **BVMD** | 31 (54.4) | 24 (58.5) | 19 (76.0) | 4 (66.7) |
| **MD** | 0 (0.0) | 0 (0.0) | 1 (4.0) | 0 (0.0) |
| **Multiple phenotypes** | 7 (12.3) | 4 (9.8) | 2 (8.0) | 0 (0.0) |
| **RD** | 1 (1.7) | 0 (0.0) | 0 (0,0) | 0 (0,0) |
| **RP** | 0 (0.0) | 1 (2.4) | 0 (0,0) | 0 (0,0) |
| **Total** | **57 (38.0)** | **41(27.3)** | **25 (16.7)** | **6 (4.0)** |

*Without counting unknown phenotypes*
