# Supplementary material for: Novel BEST1 Variant Characterization in a Large French Cohort in Light of Updated Bestrophin-1 Structure–Function Correlation
Source: Invest Ophthalmol Vis Sci. 2025 Sep 2;66(12):4. doi: 10.1167/iovs.66.12.4 (PMC12410269; doi:10.1167/iovs.66.12.4)
Supplement: Supplement 13 [file iovs-66-12-4_s013.docx]

**Supplementary Table S6: Distribution of variant types by phenotype**

**A) LOVD cohort** (Total number of variants: n=488)

|  | **ADVIRC (%)** | **AMD or MD (%)** | **ARB**  **(%)** | **AVMD**  **(%)** | **BVMD**  **(%)** | **Multiple phenotypes**  **(%)** | **RP**  **(%)** | **Other or unknown phenotypes (%)** |
| --- | --- | --- | --- | --- | --- | --- | --- | --- |
| **Missense or synonymous substitution** | 6 (100.0) | 5 (71.4) | 91 (56.2) | 4 (66.6) | 196 (96.1) | 27 (84.4) | 6 (66.7) | 38 (61.2) |
| **Deletion** | 0 (0.0) | 0 (0.0) | 8 (4.ç) | 0 (0.0) | 2 (1.0) | 1 (3.1) | 0 (0.0) | 0 (0.0) |
| **Deletion - insertion** | 0 (0.0) | 0 (0.0) | 2 (1.2) | 1 (16.7) | 1 (0.5) | 0 (0.0) | 0 (0.0) | 0 (0.0) |
| **Frameshift (deletion or duplication or insertion)** | 0 (0.0) | 0 (0.0) | 25 (15.4) | 1 (16.7) | 2 (1.0) | 3 (9.4) | 1 (1.1) | 1 (1.6) |
| **Intronic or UTR variation** | 0 (0.0) | 1 (14.3) | 2 (1.2) | 0 () | 2 (1.0) | 0 (0.0) | 2 (2.2) | 22 (35.5) |
| **Insertion (in frame)** | 0 (0.0) | 0 (0.0) | 0 (0.0) | 0 () | 0 (0.0) | 0 (0.0) | 0 (0.0) | 1 (1.6) |
| **Splice** | 0 (0.0) | 0 (0.0) | 14 (8.6) | 0 (0.0) | 1 (0.5) | 0 (0.0) | 0 (0.0) | 0 (0.0) |
| **Stop** | 0 (0.0) | 1 (14.3) | 18 (11.1) | 0 (0.0) | 0 (0.0) | 1 (3.1) | 0 (0.0) | 0 (0.0) |
| **Total truncated** | 0 (0.0) | 2 (28.6) | 71 (43.8) | 2 (33.3) | 8 (3.9) | 5 (15.6) | 3 (33.3) | 24 (38.7) |
| **Total** | **6 (1.2)** | **7 (1.4)** | **162 (33.2)** | **6 (1.2)** | **204 (41.8)** | **32 (6.6)** | **9 (1.8)** | **62 (12.7)** |

*Other phenotypes include butterfly-shaped pattern dystrophy, multifocal vitelliform dystrophy and polymorphism.*

|  | **AVMD and BVMD – n patients** | **ARB – n patients** | ***p-*value** |
| --- | --- | --- | --- |
| **Missense or synonymous substitutions** | 200 | 91 | <0.0001 |
| **Truncated variants** | 10 | 71 |  |

*p*-value was calculated using a Chi-square test.

**B) French cohort** (Total number of variants: n=150)

|  | **ADVIRC (%)** | **AMD or MD (%)** | **ARB (%)** | **AVMD (%)** | **BVMD (%)** | **Multiple phenotypes (%)** | **RP or RD (%)** |
| --- | --- | --- | --- | --- | --- | --- | --- |
| **Missense** | 1 (50.0) | 1 (100) | 29 (70.7) | 1 (50.0) | 84 (95.5) | 13 (100) | 3 (100) |
| **Deletion** | 0 (0.0) | 0 (0.0) | 2 (5.0) | 0 (0.0) | 3 (3.4) | 0 (0.0) | 0 (0.0) |
| **Deletion - insertion** | 0 (0.0) | 0 (0.0) | 1 (2.5) | 0 (0.0) | 1 (1.1) | 0 (0.0) | 0 (0.0) |
| **Stop** | 0 (0.0) | 0 (0.0) | 3 (7.5) | 0 (0.0) | 0 (0.0) | 0 (0.0) | 0 (0.0) |
| **Splice** | 1 (50.0) | 0 (0.0) | 2 (5.0) | 0 (0.0) | 0 (0.0) | 0 (0.0) | 0 (0.0) |
| **Frameshift** | 0 (0.0) | 0 (0.0) | 4 (10.0) | 1 (50.0) | 0 (0.0) | 0 (0.0) | 0 (0.0) |
| **Total truncated** | 1 (50.0) | 0 (0.0) | 12 (29.3) | 1 (50.0) | 4 (4.5) | 0 (0.0) | 0 (0.0) |
| **Total** | **2 (1.3)** | **1 (0.7)** | **41 (27.3)** | **2 (1.3)** | **88 (58.7)** | **13 (8.7)** | **3 (2.0)** |

**C. Comparison of phenotypes distribution between LOVD and French cohorts**Other or unknown phenotypes were not considered because there are no patients in the French cohort with this clinical presentation. *p*-value was calculated using a Fisher’s exact test.

|  | **LOVD – n patients** | **French cohort – n patients** | ***p-*value** |
| --- | --- | --- | --- |
| **ADVIRC** | 6 | 2 | 0.2901 |
| **AMD or MD** | 7 | 1 |  |
| **ARB** | 162 | 41 |  |
| **AVMD** | 6 | 2 |  |
| **BVMD** | 204 | 88 |  |
| **Multiple phenotypes** | 32 | 13 |  |
| **RP or RD** | 9 | 3 |  |
