# Supplementary material for: Novel BEST1 Variant Characterization in a Large French Cohort in Light of Updated Bestrophin-1 Structure–Function Correlation
Source: Invest Ophthalmol Vis Sci. 2025 Sep 2;66(12):4. doi: 10.1167/iovs.66.12.4 (PMC12410269; doi:10.1167/iovs.66.12.4)
Supplement: Supplement 15 [file iovs-66-12-4_s015.docx]

| **Supplementary Table S8: Conservation of regions of interest in bestrophin paralogs and orthologs**  Ca^2+^ clasp region is highlighted in yellow, novel French variants in pinkish and non-conserved residues in blue. | 290 | 291 | 292 | 293 | 294 | 295 | 296 | **297** | 298 | 299 | **300** | **301** | **302** | **303** | **304** | 305 | 306 | 307 | 308 | 309 | 310 | 311 | 312 | 313 | 314 | 315 | 316 |
| --- | --- | --- | --- | --- | --- | --- | --- | --- | --- | --- | --- | --- | --- | --- | --- | --- | --- | --- | --- | --- | --- | --- | --- | --- | --- | --- | --- |
| **Human** |  |  |  |  |  |  |  |  |  |  |  |  |  |  |  |  |  |  |  |  |  |  |  |  |  |  |  |
| hBest1 | V | A | E | Q | L | I | N | **P** | F | G | **E** | **D** | **D** | **D** | **D** | F | E | T | N | W | I | V | D | R | N | L | Q |
| hBest2 | V | A | E | Q | L | I | N | **P** | F | G | **E** | **D** | **D** | **D** | **D** | F | E | T | N | F | L | I | D | R | N | F | Q |
| hBest3 | V | A | E | Q | L | I | N | **P** | F | G | **E** | **D** | **D** | **D** | **D** | F | E | T | N | W | C | I | D | R | N | L | Q |
| **Chimp** |  |  |  |  |  |  |  |  |  |  |  |  |  |  |  |  |  |  |  |  |  |  |  |  |  |  |  |
| hBest1 | V | A | E | Q | L | I | N | **P** | F | G | **E** | **D** | **D** | **D** | **D** | F | E | T | N | W | I | V | D | R | N | L | Q |
| hBest2 | V | A | E | Q | L | I | N | **P** | F | G | **E** | **D** | **D** | **D** | **D** | F | E | T | N | F | L | I | D | R | N | F | Q |
| hBest3 | V | A | E | Q | L | I | N | **P** | F | G | **E** | **D** | **D** | **D** | **D** | F | E | T | N | W | C | I | D | R | N | L | Q |
| **Rat** |  |  |  |  |  |  |  |  |  |  |  |  |  |  |  |  |  |  |  |  |  |  |  |  |  |  |  |
| hBest1 | V | A | E | Q | L | I | N | **P** | F | G | **E** | **D** | **D** | **D** | **D** | F | E | T | N | W | I | **I** | D | R | N | L | Q |
| hBest2 | V | A | E | Q | L | I | N | **P** | F | G | **E** | **D** | **D** | **D** | **D** | F | E | T | N | F | L | I | D | R | N | F | Q |
| hBest3 | V | A | E | Q | L | I | N | **P** | F | G | **E** | **D** | **D** | **D** | **D** | F | E | T | N | W | C | I | D | R | N | L | Q |
| **Mouse** |  |  |  |  |  |  |  |  |  |  |  |  |  |  |  |  |  |  |  |  |  |  |  |  |  |  |  |
| hBest1 | V | A | E | Q | L | I | N | **P** | F | G | **E** | **D** | **D** | **D** | **D** | F | E | T | N | W | I | **I** | D | R | N | L | Q |
| hBest2 | V | A | E | Q | L | I | N | **P** | F | G | **E** | **D** | **D** | **D** | **D** | F | E | T | N | F | L | I | D | R | N | F | Q |
| hBest3 | V | A | E | Q | L | I | N | **P** | F | G | **E** | **D** | **D** | **D** | **D** | F | E | T | N | W | C | I | D | R | N | L | Q |
| **Dog** |  |  |  |  |  |  |  |  |  |  |  |  |  |  |  |  |  |  |  |  |  |  |  |  |  |  |  |
| hBest1 | V | A | E | Q | L | I | N | **P** | F | G | **E** | **D** | **D** | **D** | **D** | F | E | T | N | W | I | V | D | R | S | L | Q |
| hBest2 | V | A | E | Q | L | I | N | **P** | F | G | **E** | **D** | **D** | **D** | **D** | F | E | T | N | F | L | I | D | R | N | F | Q |
| hBest3 | V | A | E | Q | L | I | N | **P** | F | G | **E** | **D** | **D** | **D** | **D** | F | E | T | N | W | C | I | D | R | N | L | Q |
| **Chicken** |  |  |  |  |  |  |  |  |  |  |  |  |  |  |  |  |  |  |  |  |  |  |  |  |  |  |  |
| hBest1 | V | A | E | Q | L | I | N | **P** | F | G | **E** | **D** | **D** | **D** | **D** | F | E | T | N | W | L | **I** | D | R | N | L | Q |
| hBest2 | V | A | E | Q | L | I | N | **P** | F | G | **E** | **D** | **D** | **D** | **D** | F | E | T | N | T | L | I | D | R | N | F | Q |
| hBest3 | V | A | E | Q | L | I | N | **P** | F | G | **E** | **D** | **D** | **D** | **D** | F | E | T | N | W | C | I | D | R | N | L | Q |
| **Frog** |  |  |  |  |  |  |  |  |  |  |  |  |  |  |  |  |  |  |  |  |  |  |  |  |  |  |  |
| hBest1 | V | A | E | Q | L | I | N | **P** | F | G | **Q** | **D** | **D** | **D** | **D** | F | E | T | N | W | L | **I** | D | R | N | L | Q |
| hBest2 | V | A | E | Q | L | I | N | **P** | F | G | **E** | **D** | **D** | **D** | **D** | F | E | I | N | F | L | I | D | R | N | F | Q |
| hBest3 | V | A | E | Q | L | I | N | **P** | F | G | **E** | **D** | **D** | **D** | **D** | F | E | T | N | W | C | I | D | R | N | L | Q |
| **Tetraodon** |  |  |  |  |  |  |  |  |  |  |  |  |  |  |  |  |  |  |  |  |  |  |  |  |  |  |  |
| hBest1 | V | A | E | Q | L | I | N | **P** | F | G | **E** | **D** | **D** | **D** | **D** | F | E | T | N | W | L | V | D | R | N | L | Q |
| hBest2 | V | A | E | Q | L | I | N | **P** | F | G | **E** | **D** | **D** | **D** | **D** | F | E | T | N | W | L | I | D | R | N | F | Q |
| hBest3 | V | A | E | Q | L | I | N | **P** | F | G | **E** | **D** | **D** | **D** | **D** | F | E | A | N | W | I | I | D | R | N | L | Q |
| **Zebrafish** |  |  |  |  |  |  |  |  |  |  |  |  |  |  |  |  |  |  |  |  |  |  |  |  |  |  |  |
| hBest1 | V | A | E | Q | L | I | N | **P** | F | G | **E** | **D** | **D** | **D** | **D** | F | E | T | N | W | L | V | D | R | N | L | Q |
| hBest2 | V | A | E | Q | L | I | N | **P** | F | G | **E** | D | D | D | D | F | E | T | N | W | L | I | D | R | N | F | Q |
| hBest3 |  |  |  |  |  |  |  |  |  |  |  |  |  |  |  |  |  |  |  |  |  |  |  |  |  |  |  |
| **Fruitfly** |  |  |  |  |  |  |  |  |  |  |  |  |  |  |  |  |  |  |  |  |  |  |  |  |  |  |  |
| hBest1 | V | A | E | S | L | I | N | **P** | F | G | **E** | **D** | **D** | **D** | **D** | F | E | V | N | W | M | V | D | R | N | L | Q |
| hBest2 | V | A | E | S | L | I | N | **P** | F | G | **E** | **D** | **D** | **D** | **D** | F | E | V | N | W | M | V | D | R | N | L | Q |
| hBest3 | V | A | E | S | L | I | N | **P** | F | G | **E** | **D** | **D** | **D** | **D** | F | E | V | N | W | M | V | D | R | N | L | Q |
| ***C.elegans*** |  |  |  |  |  |  |  |  |  |  |  |  |  |  |  |  |  |  |  |  |  |  |  |  |  |  |  |
| hBest1 | V | S | E | A | L | L | N | **P** | L | G | **E** | **D** | **D** | **D** | **D** | F | E | V | N | F | L | I | D | R | N | I | Y |
| hBest2 | V | A | E | I | L | L | N | **P** | M | G | **E** | **D** | **D** | **D** | **D** | F | E | L | N | N | I | I | D | K | N | F | Y |
| hBest3 | V | S | E | A | L | L | N | **P** | L | G | **E** | **D** | **D** | **D** | **D** | F | E | V | N | F | L | I | D | R | N | I | Y |
|  | 16 | 17 | 18 | 19 | 20 | 21 | 22 | 23 | 24 | 25 | **26** | 27 | 28 | 29 | 30 | 31 | 32 | 33 | 34 | 35 | 36 |  |  |  |  |  |  |
| **Human** |  |  |  |  |  |  |  |  |  |  |  |  |  |  |  |  |  |  |  |  |  |  |  |  |  |  |  |
| hBest1 | S | F | S | R | L | L | L | C | W | R | **G** | S | I | Y | K | L | L | Y | G | E | F |  |  |  |  |  |  |
| hBest2 | G | F | S | Q | L | L | L | L | W | R | **G** | S | I | Y | K | L | L | W | R | E | L |  |  |  |  |  |  |
| hBest3 | G | F | H | R | L | L | L | K | W | R | **G** | S | I | Y | K | L | L | Y | R | E | F |  |  |  |  |  |  |
| **Chimp** |  |  |  |  |  |  |  |  |  |  |  |  |  |  |  |  |  |  |  |  |  |  |  |  |  |  |  |
| hBest1 | S | F | S | R | L | L | L | C | W | R | **G** | S | I | Y | K | L | L | Y | G | E | F |  |  |  |  |  |  |
| hBest2 | G | F | S | Q | L | L | L | L | W | R | **G** | S | I | Y | K | L | L | W | R | E | L |  |  |  |  |  |  |
| hBest3 | G | F | H | R | L | L | L | K | W | R | **G** | S | I | Y | K | L | L | Y | R | E | F |  |  |  |  |  |  |
| **Rat** |  |  |  |  |  |  |  |  |  |  |  |  |  |  |  |  |  |  |  |  |  |  |  |  |  |  |  |
| hBest1 | S | F | S | C | L | L | L | R | W | R | **G** | S | I | Y | K | L | L | Y | G | E | F |  |  |  |  |  |  |
| hBest2 | G | F | S | Q | L | L | L | L | W | R | **G** | S | I | Y | K | L | L | W | R | E | L |  |  |  |  |  |  |
| hBest3 | G | F | H | R | L | L | L | K | W | R | **G** | S | I | Y | K | L | L | Y | R | E | F |  |  |  |  |  |  |
| **Mouse** |  |  |  |  |  |  |  |  |  |  |  |  |  |  |  |  |  |  |  |  |  |  |  |  |  |  |  |
| hBest1 | S | F | S | S | L | L | L | C | W | R | **G** | S | I | Y | K | L | L | Y | G | E | F |  |  |  |  |  |  |
| hBest2 | G | F | S | Q | L | L | L | L | W | R | **G** | S | I | Y | K | L | L | W | R | E | L |  |  |  |  |  |  |
| hBest3 | G | F | H | R | L | L | L | K | W | R | **G** | S | I | Y | K | L | L | Y | R | E | F |  |  |  |  |  |  |
| **Dog** |  |  |  |  |  |  |  |  |  |  |  |  |  |  |  |  |  |  |  |  |  |  |  |  |  |  |  |
| hBest1 | S | F | S | R | L | L | L | C | W | R | **G** | S | I | Y | K | L | L | Y | G | E | F |  |  |  |  |  |  |
| hBest2 | G | F | S | Q | L | L | L | L | W | R | **G** | S | I | Y | K | L | L | W | R | E | L |  |  |  |  |  |  |
| hBest3 | G | F | H | R | L | L | L | K | W | R | **G** | S | I | Y | K | L | L | Y | R | E | F |  |  |  |  |  |  |
| **Chicken** |  |  |  |  |  |  |  |  |  |  |  |  |  |  |  |  |  |  |  |  |  |  |  |  |  |  |  |
| hBest1 | T | F | S | Q | L | L | L | Q | W | K | **G** | S | I | Y | K | L | L | Y | S | E | F |  |  |  |  |  |  |
| hBest2 |  |  |  |  |  |  |  |  |  |  |  |  |  |  |  |  |  |  |  |  |  |  |  |  |  |  |  |
| hBest3 | G | F | H | R | L | L | L | K | W | K | **G** | S | I | Y | K | L | L | Y | R | E | F |  |  |  |  |  |  |
| **Frog** |  |  |  |  |  |  |  |  |  |  |  |  |  |  |  |  |  |  |  |  |  |  |  |  |  |  |  |
| hBest1 | T | F | S | R | L | L | L | R | W | R | **G** | S | I | Y | K | L | L | Y | R | E | F |  |  |  |  |  |  |
| hBest2 |  |  |  |  |  |  |  |  |  |  |  |  |  |  |  |  |  |  |  |  |  |  |  |  |  |  |  |
| hBest3 | G | F | H | R | L | L | L | K | W | R | **G** | S | I | Y | K | L | L | Y | R | E | F |  |  |  |  |  |  |
| **Tetraodon** |  |  |  |  |  |  |  |  |  |  |  |  |  |  |  |  |  |  |  |  |  |  |  |  |  |  |  |
| hBest1 | T | F | F | H | L | L | L | R | W | R | **G** | S | I | Y | K | L | L | Y | R | E | L |  |  |  |  |  |  |
| hBest2 | S | F | S | K | L | L | L | A | W | K | **G** | S | I | Y | R | L | L | Y | K | E | F |  |  |  |  |  |  |
| hBest3 | G | F | H | R | L | L | L | K | W | R | **G** | S | I | Y | K | L | L | Y | R | E | F |  |  |  |  |  |  |
| **Zebrafish** |  |  |  |  |  |  |  |  |  |  |  |  |  |  |  |  |  |  |  |  |  |  |  |  |  |  |  |
| hBest1 | T | F | Y | R | L | L | L | R | W | K | **G** | S | I | Y | K | L | L | Y | R | E | L |  |  |  |  |  |  |
| hBest2 | G | F | S | K | L | L | L | A | W | K | **G** | S | I | Y | K | V | L | Y | K | E | F |  |  |  |  |  |  |
| hBest3 | G | F | H | R | L | L | L | K | W | R | **G** | S | I | Y | K | L | L | Y | R | E | F |  |  |  |  |  |  |
| **Fruitfly** |  |  |  |  |  |  |  |  |  |  |  |  |  |  |  |  |  |  |  |  |  |  |  |  |  |  |  |
| hBest1 | C | F | L | K | L | L | L | R | W | R | **G** | S | I | Y | K | L | V | W | L | D | L |  |  |  |  |  |  |
| hBest2 | C | F | L | K | L | L | L | R | W | R | **G** | S | I | Y | K | L | V | W | L | D | L |  |  |  |  |  |  |
| hBest3 | G | F | L | K | L | L | L | R | W | R | **G** | S | I | Y | K | L | V | W | L | D | L |  |  |  |  |  |  |
| ***C.elegans*** |  |  |  |  |  |  |  |  |  |  |  |  |  |  |  |  |  |  |  |  |  |  |  |  |  |  |  |
| hBest1 | N | F | F | K | I | L | F | R | W | K | **G** | S | V | W | K | S | I | W | K | E | L |  |  |  |  |  |  |
| hBest2 | N | F | V | S | I | M | S | Y | W | H | **G** | S | L | V | K | S | I | W | K | E | Y |  |  |  |  |  |  |
| hBest3 | N | F | F | K | I | L | F | R | W | K | **G** | S | V | W | K | S | I | W | K | E | L |  |  |  |  |  |  |
|  | 83 | 84 | 85 | 86 | 87 | 88 | 89 | **90** | 91 | 92 | 93 | 94 | 95 | 96 | 97 | 98 | 99 | 100 |  |  |  |  |  |  |  |  |  |
| **Human** |  |  |  |  |  |  |  |  |  |  |  |  |  |  |  |  |  |  |  |  |  |  |  |  |  |  |  |
| hBest1 | G | F | Y | V | T | L | V | **V** | T | R | W | W | N | Q | Y | E | N | L |  |  |  |  |  |  |  |  |  |
| hBest2 | G | F | Y | V | T | L | V | **V** | N | R | W | W | S | Q | Y | L | C | M |  |  |  |  |  |  |  |  |  |
| hBest3 | G | F | Y | V | T | L | V | **V** | N | R | W | W | N | Q | F | V | N | L |  |  |  |  |  |  |  |  |  |
| **Chimp** |  |  |  |  |  |  |  |  |  |  |  |  |  |  |  |  |  |  |  |  |  |  |  |  |  |  |  |
| hBest1 | G | F | Y | V | T | L | V | **V** | T | R | W | W | N | Q | Y | E | N | L |  |  |  |  |  |  |  |  |  |
| hBest2 | G | F | Y | V | T | L | V | **V** | N | R | W | W | S | Q | Y | L | C | M |  |  |  |  |  |  |  |  |  |
| hBest3 | G | F | Y | V | T | L | V | **V** | N | R | W | W | N | Q | F | V | N | L |  |  |  |  |  |  |  |  |  |
| **Rat** |  |  |  |  |  |  |  |  |  |  |  |  |  |  |  |  |  |  |  |  |  |  |  |  |  |  |  |
| hBest1 | G | F | Y | V | T | L | V | **V** | S | R | W | W | N | Q | Y | E | N | L |  |  |  |  |  |  |  |  |  |
| hBest2 | G | F | Y | V | T | L | V | **V** | H | R | W | W | N | Q | Y | L | C | M |  |  |  |  |  |  |  |  |  |
| hBest3 | G | F | Y | V | T | L | V | **V** | N | R | W | W | N | Q | F | V | N | L |  |  |  |  |  |  |  |  |  |
| **Mouse** |  |  |  |  |  |  |  |  |  |  |  |  |  |  |  |  |  |  |  |  |  |  |  |  |  |  |  |
| hBest1 | G | F | Y | V | T | L | V | **V** | S | R | W | W | S | Q | Y | E | N | L |  |  |  |  |  |  |  |  |  |
| hBest2 | G | F | Y | V | T | L | V | **V** | H | R | W | W | N | Q | Y | L | C | M |  |  |  |  |  |  |  |  |  |
| hBest3 | G | F | Y | V | T | L | V | **V** | N | R | W | W | N | Q | F | V | N | L |  |  |  |  |  |  |  |  |  |
| **Dog** |  |  |  |  |  |  |  |  |  |  |  |  |  |  |  |  |  |  |  |  |  |  |  |  |  |  |  |
| hBest1 | G | F | Y | V | T | L | V | **V** | T | R | W | W | N | Q | Y | E | N | L |  |  |  |  |  |  |  |  |  |
| hBest2 | G | F | Y | V | T | L | V | **V** | H | R | W | W | N | Q | Y | L | C | M |  |  |  |  |  |  |  |  |  |
| hBest3 | G | F | Y | V | T | L | V | **V** | N | R | W | W | N | Q | F | V | N | L |  |  |  |  |  |  |  |  |  |
| **Chicken** |  |  |  |  |  |  |  |  |  |  |  |  |  |  |  |  |  |  |  |  |  |  |  |  |  |  |  |
| hBest1 | G | F | Y | V | T | L | V | **V** | S | R | W | W | A | Q | Y | E | S | I |  |  |  |  |  |  |  |  |  |
| hBest2 |  |  |  |  |  |  |  |  |  |  |  |  |  |  |  |  |  |  |  |  |  |  |  |  |  |  |  |
| hBest3 | G | F | Y | V | T | L | V | **V** | N | R | W | W | N | Q | F | V | N | L |  |  |  |  |  |  |  |  |  |
| **Frog** |  |  |  |  |  |  |  |  |  |  |  |  |  |  |  |  |  |  |  |  |  |  |  |  |  |  |  |
| hBest1 | G | F | Y | V | T | L | V | **V** | S | R | W | W | G | Q | Y | E | S | V |  |  |  |  |  |  |  |  |  |
| hBest2 | G | F | Y | V | N | L | V | **V** | N | R | W | W | N | Q | Y | L | S | L |  |  |  |  |  |  |  |  |  |
| hBest3 | G | F | Y | V | T | L | V | **V** | N | R | W | W | N | Q | F | V | N | L |  |  |  |  |  |  |  |  |  |
| **Tetraodon** |  |  |  |  |  |  |  |  |  |  |  |  |  |  |  |  |  |  |  |  |  |  |  |  |  |  |  |
| hBest1 | G | F | Y | V | T | L | V | **V** | S | R | W | W | G | Q | F | E | N | V |  |  |  |  |  |  |  |  |  |
| hBest2 | G | F | Y | V | T | L | V | **V** | N | R | W | W | S | Q | Y | T | S | I |  |  |  |  |  |  |  |  |  |
| hBest3 | G | F | Y | V | T | L | V | **V** | N | R | W | W | N | Q | F | V | N | L |  |  |  |  |  |  |  |  |  |
| **Zebrafish** |  |  |  |  |  |  |  |  |  |  |  |  |  |  |  |  |  |  |  |  |  |  |  |  |  |  |  |
| hBest1 | G | F | Y | V | T | L | V | **V** | S | R | W | W | G | Q | F | E | S | V |  |  |  |  |  |  |  |  |  |
| hBest2 | G | F | Y | V | T | L | V | **V** | N | R | W | W | S | Q | Y | T | S | I |  |  |  |  |  |  |  |  |  |
| hBest3 |  |  |  |  |  |  |  |  |  |  |  |  |  |  |  |  |  |  |  |  |  |  |  |  |  |  |  |
| **Fruitfly** |  |  |  |  |  |  |  |  |  |  |  |  |  |  |  |  |  |  |  |  |  |  |  |  |  |  |  |
| hBest1 | G | F | Y | V | S | I | V | **M** | T | R | W | W | N | Q | Y | T | S | I |  |  |  |  |  |  |  |  |  |
| hBest2 | G | F | Y | V | S | I | V | **M** | T | R | W | W | N | Q | Y | T | S | I |  |  |  |  |  |  |  |  |  |
| hBest3 | G | F | Y | V | S | I | V | **M** | T | R | W | W | N | Q | Y | T | S | I |  |  |  |  |  |  |  |  |  |
| ***C.elegans*** |  |  |  |  |  |  |  |  |  |  |  |  |  |  |  |  |  |  |  |  |  |  |  |  |  |  |  |
| hBest1 | T | F | F | V | T | T | I | **V** | A | R | W | N | K | I | F | D | N | M |  |  |  |  |  |  |  |  |  |
| hBest2 | G | F | F | V | T | T | V | **I** | D | R | W | R | K | A | F | Q | N | I |  |  |  |  |  |  |  |  |  |
| hBest3 | T | F | F | V | T | T | I | **V** | A | R | W | N | K | I | F | D | N | M |  |  |  |  |  |  |  |  |  |
|  | 127 | 128 | 129 | 130 | 131 | 132 | 133 | 134 | 135 | 136 | **137** | 138 | 139 | 140 | 141 | 142 | 143 | 144 | 145 | 146 | 147 |  |  |  |  |  |  |
| **Human** |  |  |  |  |  |  |  |  |  |  |  |  |  |  |  |  |  |  |  |  |  |  |  |  |  |  |  |
| hBest1 | T | L | I | R | Y | A | N | L | G | N | **V** | L | I | L | R | S | V | S | T | A | V |  |  |  |  |  |  |
| hBest2 | T | L | M | R | Y | A | G | L | S | A | **V** | L | I | L | R | S | V | S | T | A | V |  |  |  |  |  |  |
| hBest3 | T | L | M | R | Y | V | N | L | T | S | **L** | L | I | F | R | S | V | S | T | A | V |  |  |  |  |  |  |
| **Chimp** |  |  |  |  |  |  |  |  |  |  |  |  |  |  |  |  |  |  |  |  |  |  |  |  |  |  |  |
| hBest1 | T | L | I | R | Y | A | N | L | G | N | **V** | L | I | L | R | S | V | S | T | A | V |  |  |  |  |  |  |
| hBest2 | T | L | M | R | Y | A | G | L | S | A | **V** | L | I | L | R | S | V | S | T | A | V |  |  |  |  |  |  |
| hBest3 | T | L | M | R | Y | V | N | L | T | S | **L** | L | I | F | R | S | V | S | T | A | V |  |  |  |  |  |  |
| **Rat** |  |  |  |  |  |  |  |  |  |  |  |  |  |  |  |  |  |  |  |  |  |  |  |  |  |  |  |
| hBest1 | T | L | I | R | Y | A | I | L | G | Q | **V** | L | I | L | R | S | I | S | T | S | V |  |  |  |  |  |  |
| hBest2 | T | L | M | R | Y | A | G | L | S | A | **V** | L | I | L | R | S | V | S | T | A | V |  |  |  |  |  |  |
| hBest3 | T | L | M | R | Y | V | N | L | T | S | **L** | L | I | F | R | S | V | S | T | A | V |  |  |  |  |  |  |
| **Mouse** |  |  |  |  |  |  |  |  |  |  |  |  |  |  |  |  |  |  |  |  |  |  |  |  |  |  |  |
| hBest1 | T | L | I | R | Y | A | I | L | G | Q | **V** | L | I | L | R | S | I | S | T | S | V |  |  |  |  |  |  |
| hBest2 | T | L | M | R | Y | A | G | L | S | A | **V** | L | I | L | R | S | V | S | T | A | V |  |  |  |  |  |  |
| hBest3 | T | L | M | R | Y | V | N | L | T | S | **L** | L | I | F | R | S | V | S | T | A | V |  |  |  |  |  |  |
| **Dog** |  |  |  |  |  |  |  |  |  |  |  |  |  |  |  |  |  |  |  |  |  |  |  |  |  |  |  |
| hBest1 | T | L | I | R | Y | A | N | L | G | N | **V** | L | I | L | R | S | V | S | A | A | V |  |  |  |  |  |  |
| hBest2 | T | L | M | R | Y | A | G | L | S | A | **V** | L | I | L | R | S | V | S | T | A | V |  |  |  |  |  |  |
| hBest3 | T | L | M | R | Y | V | N | L | T | S | **L** | L | I | F | R | S | V | S | T | A | V |  |  |  |  |  |  |
| **Chicken** |  |  |  |  |  |  |  |  |  |  |  |  |  |  |  |  |  |  |  |  |  |  |  |  |  |  |  |
| hBest1 | T | L | M | R | Y | S | N | L | C | S | **V** | L | I | L | R | S | V | S | T | A | V |  |  |  |  |  |  |
| hBest2 |  |  |  |  |  |  |  |  |  |  |  |  |  |  |  |  |  |  |  |  |  |  |  |  |  |  |  |
| hBest3 | T | L | M | R | Y | V | N | L | T | S | **L** | L | I | F | R | S | V | S | T | A | V |  |  |  |  |  |  |
| **Frog** |  |  |  |  |  |  |  |  |  |  |  |  |  |  |  |  |  |  |  |  |  |  |  |  |  |  |  |
| hBest1 | T | L | M | R | Y | A | N | L | T | G | **L** | L | I | L | R | S | V | S | T | A | V |  |  |  |  |  |  |
| hBest2 | T | L | M | R | Y | C | S | L | S | G | **L** | L | I | L | R | S | V | S | T | A | A |  |  |  |  |  |  |
| hBest3 | T | L | M | R | Y | V | N | L | T | S | **L** | L | I | F | R | S | V | S | T | A | V |  |  |  |  |  |  |
| **Tetraodon** |  |  |  |  |  |  |  |  |  |  |  |  |  |  |  |  |  |  |  |  |  |  |  |  |  |  |  |
| hBest1 | T | L | M | R | Y | A | N | L | S | G | **V** | L | I | Y | R | S | V | S | T | A | V |  |  |  |  |  |  |
| hBest2 | T | M | M | R | Y | A | S | L | S | A | **L** | L | I | L | R | S | V | S | T | A | V |  |  |  |  |  |  |
| hBest3 | T | L | V | R | Y | V | N | L | T | S | **L** | L | I | F | R | S | V | S | T | A | V |  |  |  |  |  |  |
| **Zebrafish** |  |  |  |  |  |  |  |  |  |  |  |  |  |  |  |  |  |  |  |  |  |  |  |  |  |  |  |
| hBest1 | S | L | M | R | Y | A | N | L | S | G | **I** | L | I | Y | R | S | V | S | T | A | V |  |  |  |  |  |  |
| hBest2 | T | L | M | R | Y | S | S | L | S | A | **V** | L | I | L | R | S | V | S | T | A | V |  |  |  |  |  |  |
| hBest3 |  |  |  |  |  |  |  |  |  |  |  |  |  |  |  |  |  |  |  |  |  |  |  |  |  |  |  |
| **Fruitfly** |  |  |  |  |  |  |  |  |  |  |  |  |  |  |  |  |  |  |  |  |  |  |  |  |  |  |  |
| hBest1 | T | I | M | R | Y | V | C | L | C | L | **T** | M | V | L | A | N | V | S | P | R | V |  |  |  |  |  |  |
| hBest2 | T | I | M | R | Y | V | C | L | C | L | **T** | M | V | L | A | N | V | S | P | R | V |  |  |  |  |  |  |
| hBest3 | T | I | M | R | Y | V | C | L | C | L | **T** | M | V | L | A | N | V | S | P | R | V |  |  |  |  |  |  |
| ***C.elegans*** |  |  |  |  |  |  |  |  |  |  |  |  |  |  |  |  |  |  |  |  |  |  |  |  |  |  |  |
| hBest1 | T | I | I | R | Y | L | V | A | S | Q | **V** | L | V | L | R | T | I | S | M | R | T |  |  |  |  |  |  |
| hBest2 | T | I | I | R | Y | L | V | L | S | Q | **I** | L | L | F | R | E | I | S | T | K | V |  |  |  |  |  |  |
| hBest3 | T | I | I | R | Y | L | V | A | S | Q | **V** | L | V | L | R | T | I | S | M | R | T |  |  |  |  |  |  |
|  | 118 | 119 | 220 | 221 | 222 | 223 | 224 | 225 | 226 | **227** | 228 | 229 | **230** | 231 | 232 | 233 | 234 | 235 | 236 | 237 | 238 |  |  |  |  |  |  |
| **Human** |  |  |  |  |  |  |  |  |  |  |  |  |  |  |  |  |  |  |  |  |  |  |  |  |  |  |  |
| hBest1 | R | T | Q | C | G | H | L | Y | A | **Y** | D | W | **I** | S | I | P | L | V | Y | T | Q |  |  |  |  |  |  |
| hBest2 | R | G | K | C | G | M | L | F | H | **Y** | D | W | **I** | S | V | P | L | V | Y | T | Q |  |  |  |  |  |  |
| hBest3 | R | S | W | C | S | L | L | F | G | **Y** | D | W | **V** | G | I | P | L | V | Y | T | Q |  |  |  |  |  |  |
| **Chimp** |  |  |  |  |  |  |  |  |  |  |  |  |  |  |  |  |  |  |  |  |  |  |  |  |  |  |  |
| hBest1 | R | T | Q | C | G | H | L | Y | A | **Y** | D | W | **I** | S | I | P | L | V | Y | T | Q |  |  |  |  |  |  |
| hBest2 | R | G | K | C | G | M | L | F | H | **Y** | D | W | **I** | S | V | P | L | V | Y | T | Q |  |  |  |  |  |  |
| hBest3 | R | S | W | C | S | L | L | F | G | **Y** | D | W | **V** | G | I | P | L | V | Y | T | Q |  |  |  |  |  |  |
| **Rat** |  |  |  |  |  |  |  |  |  |  |  |  |  |  |  |  |  |  |  |  |  |  |  |  |  |  |  |
| hBest1 | R | T | Q | C | G | H | L | Y | A | **Y** | D | W | **I** | N | I | P | L | V | Y | T | Q |  |  |  |  |  |  |
| hBest2 | R | S | K | C | G | M | L | F | H | **Y** | D | W | **I** | S | I | P | L | V | Y | T | Q |  |  |  |  |  |  |
| hBest3 | R | S | W | C | S | L | L | F | G | **Y** | D | W | **V** | G | I | P | L | V | Y | T | Q |  |  |  |  |  |  |
| **Mouse** |  |  |  |  |  |  |  |  |  |  |  |  |  |  |  |  |  |  |  |  |  |  |  |  |  |  |  |
| hBest1 | R | T | Q | C | G | Q | L | Y | A | **Y** | D | W | **I** | S | I | P | L | V | Y | T | Q |  |  |  |  |  |  |
| hBest2 | R | S | K | C | G | M | L | F | H | **Y** | D | W | **I** | S | I | P | L | V | Y | T | Q |  |  |  |  |  |  |
| hBest3 | R | S | W | C | S | L | L | F | G | **Y** | D | W | **V** | G | I | P | L | V | Y | T | Q |  |  |  |  |  |  |
| **Dog** |  |  |  |  |  |  |  |  |  |  |  |  |  |  |  |  |  |  |  |  |  |  |  |  |  |  |  |
| hBest1 | R | T | Q | C | G | H | L | Y | A | **Y** | D | W | **I** | S | I | P | L | V | Y | T | Q |  |  |  |  |  |  |
| hBest2 | R | G | K | C | G | M | L | F | H | **Y** | D | W | **I** | S | V | P | L | V | Y | T | Q |  |  |  |  |  |  |
| hBest3 | R | S | W | C | S | L | L | F | G | **Y** | D | W | **V** | G | I | P | L | V | Y | T | Q |  |  |  |  |  |  |
| **Chicken** |  |  |  |  |  |  |  |  |  |  |  |  |  |  |  |  |  |  |  |  |  |  |  |  |  |  |  |
| hBest1 | R | S | Q | C | G | R | L | Y | G | **Y** | D | W | **I** | S | I | P | L | V | Y | T | Q |  |  |  |  |  |  |
| hBest2 | R | A | N | C | S | L | L | F | H | **Y** | D | W | **I** | S | V | P | L | V | Y | T | Q |  |  |  |  |  |  |
| hBest3 | R | S | W | C | S | L | L | F | G | **Y** | D | W | **V** | G | I | P | L | V | Y | T | Q |  |  |  |  |  |  |
| **Frog** |  |  |  |  |  |  |  |  |  |  |  |  |  |  |  |  |  |  |  |  |  |  |  |  |  |  |  |
| hBest1 | R | T | Q | C | G | R | L | Y | G | **Y** | D | W | **I** | S | V | P | L | V | Y | T | Q |  |  |  |  |  |  |
| hBest2 | R | G | N | C | G | M | L | F | H | **Y** | D | W | **I** | S | V | P | L | V | Y | T | Q |  |  |  |  |  |  |
| hBest3 | R | S | W | C | S | L | L | F | G | **Y** | D | W | **V** | G | I | P | L | V | Y | T | Q |  |  |  |  |  |  |
| **Tetraodon** |  |  |  |  |  |  |  |  |  |  |  |  |  |  |  |  |  |  |  |  |  |  |  |  |  |  |  |
| hBest1 | R | A | K | C | M | K | L | Y | G | **Y** | D | W | **I** | S | L | P | L | V | Y | T | Q |  |  |  |  |  |  |
| hBest2 | R | G | K | C | S | M | L | F | H | **Y** | D | M | **I** | S | V | P | L | V | Y | T | Q |  |  |  |  |  |  |
| hBest3 | R | T | S | C | S | T | L | F | G | **Y** | D | W | **V** | G | V | P | L | V | Y | T | Q |  |  |  |  |  |  |
| **Zebrafish** |  |  |  |  |  |  |  |  |  |  |  |  |  |  |  |  |  |  |  |  |  |  |  |  |  |  |  |
| hBest1 | R | S | Q | C | M | R | L | Y | G | **Y** | D | W | **I** | S | L | P | L | V | Y | T | Q |  |  |  |  |  |  |
| hBest2 | R | G | K | C | S | M | L | F | H | **Y** | D | M | **I** | S | V | P | L | V | Y | T | Q |  |  |  |  |  |  |
| hBest3 |  |  |  |  |  |  |  |  |  |  |  |  |  |  |  |  |  |  |  |  |  |  |  |  |  |  |  |
| **Fruitfly** |  |  |  |  |  |  |  |  |  |  |  |  |  |  |  |  |  |  |  |  |  |  |  |  |  |  |  |
| hBest1 | R | G | Q | C | G | L | L | I | S | **Y** | D | T | **I** | S | V | P | L | V | Y | T | Q |  |  |  |  |  |  |
| hBest2 | R | G | Q | C | G | L | L | I | S | **Y** | D | T | **I** | S | V | P | L | V | Y | T | Q |  |  |  |  |  |  |
| hBest3 | R | G | Q | C | G | L | L | I | S | **Y** | D | T | **I** | S | V | P | L | V | Y | T | Q |  |  |  |  |  |  |
| ***C.elegans*** |  |  |  |  |  |  |  |  |  |  |  |  |  |  |  |  |  |  |  |  |  |  |  |  |  |  |  |
| hBest1 | Q | A | S | I | S | V | V | K | N | **A** | D | W | **V** | P | I | P | L | A | Y | P | Q |  |  |  |  |  |  |
| hBest2 | Q | V | K | L | S | L | L | R | N | **G** | D | F | **I** | P | I | P | L | A | Y | P | Q |  |  |  |  |  |  |
| hBest3 | Q | A | S | I | S | V | V | K | N | **A** | D | W | **V** | P | I | P | L | A | Y | P | Q |  |  |  |  |  |  |
