# Supplementary material for: Novel BEST1 Variant Characterization in a Large French Cohort in Light of Updated Bestrophin-1 Structure–Function Correlation
Source: Invest Ophthalmol Vis Sci. 2025 Sep 2;66(12):4. doi: 10.1167/iovs.66.12.4 (PMC12410269; doi:10.1167/iovs.66.12.4)
Supplement: Supplement 17 [file iovs-66-12-4_s017.docx]

**Novel *BEST1*-variants characterization in a large French cohort in light of updated Bestrophin-1 structure function correlation**

Bitan J^1^, Poncet AF^1^, Lecigne C^1^, Devos A^1^, Meunier I^2,3^, Zanlonghi X^4^, Grunewald O^1^, Smirnov V^1,5*^, Dhaenens CM^1*^

* Co-author

***Supplementary Results***

***Update on the general organization and function of the BEST1 channel***

BEST1 is a plasma membrane-localized integral membrane protein^1^, with four transmembrane helices (TM1 to TM4) and intracellular N- and C-terminal regions, the latter of which is a large cytosolic domain, that is highly conserved among eukaryotic species and composed of approximately 296 amino acids (aa 289-585) ^2,3^. TM1 and TM2 as well as TM3 and TM4 are connected by a short extracellular interhelix loop whereas the intracellular connection between TM2 and TM3 is long and consists of seven helices (see structure organization synthesis in Supplementary Table 1).

BEST1 functions as a calcium-activated chloride channel ^4–11^. The channel is a homopentamer containing a unique central pore for ion conductance ^2,3^ (Figure 1).

From the extracellular entry pathway, ions crossing the pore first encounter the neck. This hydrophobic gate is formed by three highly conserved hydrophobic residues, Ile76, Phe80 and Phe84. These residues are located between the end of the second transmembrane alpha helix (TM) and the start of the next cytosolic loop from each subunit. They point towards the central axis of the channel. These residues are a hot spot of disease variations ^12^. Their role is to selectively exclude the cations but allow small anions to pass through. The neck is a gate that controls chloride permeation in a Ca^2+^-dependent manner ^13^.

Indeed, each BEST1 protomer presents a calcium-binding site, called the Ca^2+^ clasp ^2^. The five Ca^2+^ clasps form a belt around the pore on the cytosolic side of the channel below the neck region. Ca^2+^ is coordinated with a pyramidal geometry involving residues Pro297, Glu300 and Asp301 to 304, forming a highly conserved calcium-binding site ^13^. Two conserved aspartate residues, 301 and 304, directly coordinate Ca^2+^ in the clasp via their side chains. When they are mutated, the chloride current is abolished ^14^. The Ca^2+^ clasp acts as a sensor of cytoplasmic Ca^2+^, directing the opening movements of the neck, which widens in the presence of Ca^2+^. One of the most studied *BEST1* mutants, p.(Trp93Cys), affects the residue Trp93, which is close to the Ca^2+^ clasp region. The neck region is a key regulator of Ca^2+^-dependent chloride conduction, as demonstrated by mutating the three neck residues to alanine (Ile76Ala/Phe80Ala/Phe84Ala). The "3A" mutated BEST1 channel remains active in conducting Cl^-^ despite the absence of Ca^2+^ ^13,15,16^. These mutants should remove the constriction obstacle^13,15^. For instance, mutating Phe80 with a hydrophobic residue of the same size, p.(Phe80Ile) or p.(Phe80Leu) (a disease mutation) impairs the ion flux through the channel. The positive charge of phenylalanine residues may favor the interaction with negatively charged species.

The neck is followed by a wider cavity, called the "inner cavity", which is highly positively charged and whose purpose is to attract anions from inside the cell. Below the inner cavity, at the cytosolic end of the pore, is the second constriction site, the cytosolic aperture, formed by the Ile205/Gln208/Asn212 residues from each subunit ^17^. The aperture acts as a size-selective filter (involving the hydrophobic residue Ile205) but not as a charge-selective filter. This allows the passage of small ions, such as partially dehydrated chloride ions, while excluding larger molecules, like amino acids ^13^. Mutation of the pore’s cytosolic aperture does not affect the channel’s dependence on Ca^2+^ or its preference for anions over cations. However, variation alters the relative permeabilities of anions. It should be noted that fifteen binding sites for Cl^-^ have been identified in the pore (three on each SU). The final region of interest is the autoinhibitory segment (AS), which encircles the periphery of the cytosolic domain of the BEST channel in the closed state, but detachment of certain areas allows the channel to open. It consists of an anchor (residues 356-367), which remains at least partially bound to the channel periphery, and two AS cooperativity regions (ACR1, residues 346-355; and ACR2, residues 368-378), which dissociate synchronously from the channel periphery to allow neck dilation, in the presence of Ca^2+^. The AS is a critical gating element that works with the Ca^2+^ sensor to control neck closure/opening. In its absence, the channel neck remains in a fully open conformation^17^. It is important to note that no 3D structure has visualized the C-terminal domain beyond amino acid residue 379. Unlike the N-terminal domain, the cytosolic C-terminal domain is less evolutionarily conserved. Studies have shown that calcium-dependent activation of BEST1 channels can be modulated by the C-terminal region, and that deleting it reduces Ca^2+^-dependent current activation. Moreover, the C-terminal domain could regulate the cellular expression and targeting of BEST1 channels to the plasma membrane.^18^

**References**

1. Marmorstein AD, Marmorstein LY, Rayborn M, Wang X, Hollyfield JG, Petrukhin K. Bestrophin, the product of the Best vitelliform macular dystrophy gene (VMD2), localizes to the basolateral plasma membrane of the retinal pigment epithelium. *Proc Natl Acad Sci U S A*. 2000;97(23):12758-12763. doi:10.1073/pnas.220402097

2. Kane Dickson V, Pedi L, Long SB. Structure and insights into the function of a Ca(2+)-activated Cl(-) channel. *Nature*. 2014;516(7530):213-218. doi:10.1038/nature13913

3. Yang T, Liu Q, Kloss B, et al. Structure and selectivity in bestrophin ion channels. *Science*. 2014;346(6207):355-359. doi:10.1126/science.1259723

4. Marmorstein AD, Kinnick TR, Stanton JB, Johnson AA, Lynch RM, Marmorstein LY. Bestrophin-1 influences transepithelial electrical properties and Ca2+ signaling in human retinal pigment epithelium. *Mol Vis*. 2015;21:347-359.

5. Milenkovic A, Brandl C, Milenkovic VM, et al. Bestrophin 1 is indispensable for volume regulation in human retinal pigment epithelium cells. *Proc Natl Acad Sci U S A*. 2015;112(20):E2630-2639. doi:10.1073/pnas.1418840112

6. Singh R, Shen W, Kuai D, et al. iPS cell modeling of Best disease: insights into the pathophysiology of an inherited macular degeneration. *Hum Mol Genet*. 2013;22(3):593-607. doi:10.1093/hmg/dds469

7. Moshfegh Y, Velez G, Li Y, Bassuk AG, Mahajan VB, Tsang SH. BESTROPHIN1 mutations cause defective chloride conductance in patient stem cell-derived RPE. *Hum Mol Genet*. 2016;25(13):2672-2680. doi:10.1093/hmg/ddw126

8. Rosenthal R, Bakall B, Kinnick T, et al. Expression of bestrophin-1, the product of the VMD2 gene, modulates voltage-dependent Ca2+ channels in retinal pigment epithelial cells. *FASEB J*. 2006;20(1):178-180. doi:10.1096/fj.05-4495fje

9. Burgess R, Millar ID, Leroy BP, et al. Biallelic mutation of BEST1 causes a distinct retinopathy in humans. *Am J Hum Genet*. 2008;82(1):19-31. doi:10.1016/j.ajhg.2007.08.004

10. Marmorstein LY, Wu J, McLaughlin P, et al. The light peak of the electroretinogram is dependent on voltage-gated calcium channels and antagonized by bestrophin (best-1). *J Gen Physiol*. 2006;127(5):577-589. doi:10.1085/jgp.200509473

11. Zhang Y, Stanton JB, Wu J, et al. Suppression of Ca2+ signaling in a mouse model of Best disease. *Hum Mol Genet*. 2010;19(6):1108-1118. doi:10.1093/hmg/ddp583

12. Milenkovic VM, Krejcova S, Reichhart N, Wagner A, Strauss O. Interaction of bestrophin-1 and Ca2+ channel β-subunits: identification of new binding domains on the bestrophin-1 C-terminus. *PLoS One*. 2011;6(4):e19364. doi:10.1371/journal.pone.0019364

13. Vaisey G, Miller AN, Long SB. Distinct regions that control ion selectivity and calcium-dependent activation in the bestrophin ion channel. *Proc Natl Acad Sci U S A*. 2016;113(47):E7399-E7408. doi:10.1073/pnas.1614688113

14. Xiao Q, Prussia A, Yu K, Cui Y yuan, Hartzell HC. Regulation of bestrophin Cl channels by calcium: role of the C terminus. *J Gen Physiol*. 2008;132(6):681-692. doi:10.1085/jgp.200810056

15. Ji C, Kittredge A, Hopiavuori A, et al. Dual Ca2+-dependent gates in human Bestrophin1 underlie disease-causing mechanisms of gain-of-function mutations. *Commun Biol*. 2019;2:240. doi:10.1038/s42003-019-0433-3

16. Owji AP, Zhao Q, Ji C, et al. Structural and functional characterization of the bestrophin-2 anion channel. *Nat Struct Mol Biol*. 2020;27(4):382-391. doi:10.1038/s41594-020-0402-z

17. Owji AP, Wang J, Kittredge A, et al. Structures and gating mechanisms of human bestrophin anion channels. *Nat Commun*. 2022;13(1):3836. doi:10.1038/s41467-022-31437-7

18. Kim KW, Hwang J, Kim DH, Park H, Lim HH. Cytosolic domain regulates the calcium sensitivity and surface expression of BEST1 channels in the HEK293 cells. *BMB Rep*. 2023;56(2):172-177. doi:10.5483/BMBRep.2022-0170
