# Supplementary material for: Novel BEST1 Variant Characterization in a Large French Cohort in Light of Updated Bestrophin-1 Structure–Function Correlation
Source: Invest Ophthalmol Vis Sci. 2025 Sep 2;66(12):4. doi: 10.1167/iovs.66.12.4 (PMC12410269; doi:10.1167/iovs.66.12.4)
Supplement: Supplement 18 [file iovs-66-12-4_s018.docx]

**Novel *BEST1*-variants characterization in a large French cohort in light of updated Bestrophin-1 structure function correlation**

Bitan J*^1^, Poncet AF*^1^, Lecigne C^1^, Devos A^1^, Meunier I^2,3^, Zanlonghi X^4^, Grunewald O^1^, Smirnov V^1,5*^, Dhaenens CM^1*^

* Co-author

**Supplementary material and methods**

***Literature search and data collection***

All publications reporting *BEST1* variants in patients with inherited retinal disease (IRD) until December 2024 were uploaded to the Leiden Open Variation Database (LOVD) (https://databases.lovd.nl/shared/genes/BEST1). The following keywords were used for the PubMed search: "BEST1" AND "VMD2" AND "bestrophin". The last LOVD version used was 29^th^ October 2024. The HGMD professional database (version 2024.3) was also used to identify missing variants in the LOVD database. In this study we reported all unique variants from the LOVD database, using the HGVS nomenclature (https://hgvs-nomenclature.org/stable/recommendations). Duplicates were removed and nomenclature errors corrected.

***Patients***

We reported *BEST1* variants identified in the Lille University Hospital Laboratory. These variants were identified in a French cohort of 15,000 patients with inherited retinal dystrophies (IRD) analyzed between 2008 and 2024. They were referred to our laboratory with diagnoses of "Best Macular Dystrophy", "Macular Dystrophy", "Bestrophinopathy", "Retinitis Pigmentosa" or "Retinal Dystrophy". They included only affected probands. All patients carried one or two *BEST1* variants. Written informed consent was obtained from all participants before enrolment in accordance with the Declaration of Helsinki. The DNA collection from the Lille OPH-database received the agreement number DEC22-323 and the use of the Lille data was approved under the Authorization code CNIL DR-2023-061.

***Clinical examination***

Clinical data were retrospectively collected from medical records, it includes: best-corrected visual acuity (BCVA) assessed by the Early Treatment Diabetic Retinopathy Study (ETDRS) chart, refractive error, slit-lamp biomicroscopy, static and kinetic visual fields (VFs), full-field electroretinogram (ffERG) using MonColor® unit (Métrovision, Perenchies, France), high-definition domain optical coherence tomography (SD-OCT) using Spectralis OCT (Heidelberg Engineering, Inc., Heidelberg, Germany), fundus photography, short wavelength autofluorescence (SWAF) and infrared reflectance (IRR) fundus imaging using Heidelberg Retinal Tomograph (Heidelberg Engineering, Inc., Heidelberg, Germany).

***Variants identification in our laboratory***

Genetic diagnosis was performed using multiple techniques: HaloPlex-based next-generation sequencing of 150, 208 or 230 IRD-associated genes on the Ion Torrent/NextSeq and Nova platforms, and Sanger sequencing of the 11 coding exons of the *BEST1* gene. CNVs were detected by MLPA (SALSA^®^ MLPA^®^ Probemix P367, MRC Holland, The Netherlands), or based on NGS data. Segregation analyses or confirmations were conducted using direct Sanger sequencing.

***Variants analysis***

LOVD and French variants have been collected and analyzed exhaustively. A large amount of informations is provided in the form of a multi-tabbed table comprising: cDNA and protein nomenclature, type of variant, mode of inheritance, variant in trans if recessive inheritance, information on patients (number, age, sex, affected or not, history family), number of cases harboring the variant, phenotype, exon, physico-chemical deviation, conservation, intronic position, protein domain, if variant already reported, if yes the first publication is given, number of publications, countries of the population studied, ACMG classification, CADD raw and phred score, MPA score, MPA impact, SPIP risk results, SPIP interpretation, SpliceAI scores, SpliceAI 500 scores, GnomAD exome, GnomAD genome, GnomAD V4 exome, GnomAD V4 genome, DynaMut2 predictions, MetaDome score, other comments. For LOVD variants, all clinical information in the publications has been indicated.

***Protein organization, in silico predictions and variant pathogenicity classification***

Protein organization was defined based on the latest Uniprot version (<https://www.uniprot.org/uniprotkb/O76090/feature-viewer>). The BEST1 channel’s 3D structure and the changes in chemical interactions due to amino acid substitutions were assessed using AlphaFold (https://alphafold.ebi.ac.uk/entry/O76090), UCSF ChimeraX software version: 1.7.1 (2024-01-23) and DynaMut2 software (http://biosig.unimelb.edu.au/dynamut2) using PDB file 8d1k. We used DynaMut2 to obtain graphical representations and stability changes. DynaMu2 predicts the effects of missense mutations on protein stability and dynamics. Predicted stability changes are indicated by the change in Gibbs free energy of unfolding (ΔΔG, kcal/mol), where ΔG represents the effect of the variant on protein stability. ΔΔG is the difference in unfolding free energy between two proteins: the wild-type and the mutant. The ΔΔG values for all variants are provided. Pathogenicity classification was performed according to ACMG criteria ^16^ and using semi-automated classification tools, then manually rechecked for each variant by adjusting the strong/moderate/supporting criteria and adding those that could not be included automatically (notably familial segregation). This classification was performed in parallel by two or three independent experts form the laboratory, and the results were compared. The semi-automated tools used were: Varsome (https://varsome.com/); Hg38 Intervar and Genebe via Mobidetails (https://mobidetails.chu-montpellier.fr/). Changes in chemical interaction observed from the ChimeraX and DynaMut2 softwares, were used to implement the ACMG classification (PM1 and PP3 criteria notably).

***Review of functionally tested BEST1 variants from literature***

All variants reported in human disorders and with effects on trafficking to the RPE plasma membrane, oligomerization, channel activity, and intracellular calcium signaling were collected from the literature.

***Statistical analysis***

Statistical analyses were performed using GraphPad Prism software (version 10.4.1 for macOS; GraphPad Software, San Diego, CA, USA). In the LOVD cohort, the Chi-square test was used to compare the distribution of missense and synonymous variants versus truncating variants in the main protein domains (TM2-TM3 loop, C-terminal, N-terminal and TM3), as well as to assess the potential association between variant type and the clinical phenotype of bestrophinopathies. In the French cohort, due to the small sample size and the presence of expected cell frequencies below 5 in some groups, Fisher's exact test was applied to assess the distribution of variant types in the corresponding protein domains. Fisher's exact test was also used to compare the distribution of clinical phenotypes between the LOVD and French cohorts. All statistical tests were two-tailed, and p-values < 0.05 were considered statistically significant.
